# The transcriptional landscape of age in human peripheral blood

**DOI:** 10.1038/ncomms9570

**Published:** 2015-10-22

**Authors:** Marjolein J. Peters, Roby Joehanes, Luke C. Pilling, Claudia Schurmann, Karen N. Conneely, Joseph Powell, Eva Reinmaa, George L. Sutphin, Alexandra Zhernakova, Katharina Schramm, Yana A. Wilson, Sayuko Kobes, Taru Tukiainen, NABEC/UKBEC Consortium, Michael A. Nalls, Dena G. Hernandez, Mark R. Cookson, Raphael J. Gibbs, John Hardy, Adaikalavan Ramasamy, Alan B. Zonderman, Allissa Dillman, Bryan Traynor, Colin Smith, Dan L. Longo, Daniah Trabzuni, Juan Troncoso, Marcel van der Brug, Michael E. Weale, Richard O'Brien, Robert Johnson, Robert Walker, Ronald H. Zielke, Sampath Arepalli, Mina Ryten, Andrew B. Singleton, Yolande F. Ramos, Harald H. H. Göring, Myriam Fornage, Yongmei Liu, Sina A. Gharib, Barbara E. Stranger, Philip L. De Jager, Abraham Aviv, Daniel Levy, Joanne M. Murabito, Peter J. Munson, Tianxiao Huan, Albert Hofman, André G. Uitterlinden, Fernando Rivadeneira, Jeroen van Rooij, Lisette Stolk, Linda Broer, Michael M. P. J. Verbiest, Mila Jhamai, Pascal Arp, Andres Metspalu, Liina Tserel, Lili Milani, Nilesh J. Samani, Pärt Peterson, Silva Kasela, Veryan Codd, Annette Peters, Cavin K. Ward-Caviness, Christian Herder, Melanie Waldenberger, Michael Roden, Paula Singmann, Sonja Zeilinger, Thomas Illig, Georg Homuth, Hans-Jörgen Grabe, Henry Völzke, Leif Steil, Thomas Kocher, Anna Murray, David Melzer, Hanieh Yaghootkar, Stefania Bandinelli, Eric K. Moses, Jack W. Kent, Joanne E. Curran, Matthew P. Johnson, Sarah Williams-Blangero, Harm-Jan Westra, Allan F. McRae, Jennifer A. Smith, Sharon L. R. Kardia, Iiris Hovatta, Markus Perola, Samuli Ripatti, Veikko Salomaa, Anjali K. Henders, Nicholas G. Martin, Alicia K. Smith, Divya Mehta, Elisabeth B. Binder, K Maria Nylocks, Elizabeth M. Kennedy, Torsten Klengel, Jingzhong Ding, Astrid M. Suchy-Dicey, Daniel A. Enquobahrie, Jennifer Brody, Jerome I. Rotter, Yii-Der I. Chen, Jeanine Houwing-Duistermaat, Margreet Kloppenburg, P. Eline Slagboom, Quinta Helmer, Wouter den Hollander, Shannon Bean, Towfique Raj, Noman Bakhshi, Qiao Ping Wang, Lisa J. Oyston, Bruce M. Psaty, Russell P. Tracy, Grant W. Montgomery, Stephen T. Turner, John Blangero, Ingrid Meulenbelt, Kerry J. Ressler, Jian Yang, Lude Franke, Johannes Kettunen, Peter M. Visscher, G. Gregory Neely, Ron Korstanje, Robert L. Hanson, Holger Prokisch, Luigi Ferrucci, Tonu Esko, Alexander Teumer, Joyce B. J. van Meurs, Andrew D. Johnson

**Affiliations:** 1 Department of Internal Medicine, Erasmus Medical Centre Rotterdam, Rotterdam 3000CA, The Netherlands; 2 The National Heart, Lung, and Blood Institute's and Boston University's Framingham Heart Study, Framingham, Massachusetts 01702, USA; 3 Population Sciences Branch, Division of Intramural Research, National Heart, Lung, and Blood Institute, Bethesda, Maryland 20817, USA; 4 Epidemiology and Public Health, University of Exeter Medical School, Exeter EX4 1DB, UK; 5 Department of Functional Genomics, Interfaculty Institute for Genetics and Functional Genomics, University Medicine Greifswald, Greifswald 17493, Germany; 6 The Charles Bronfman Institute for Personalized Medicine, Genetics of Obesity & Related Metabolic Traits Program, Icahn School of Medicine at Mount Sinai, One Gustave L. Levy Place, New York 10029, USA; 7 Department of Human Genetics, School of Medicine, Emory University, Atlanta, Georgia 30301, USA; 8 Centre for Neurogenetics and Statistical Genomics, Queensland Brain Institute, University of Queensland, St Lucia, Brisbane, Queensland 4000, Australia; 9 The Institute for Molecular Bioscience, University of Queensland, Brisbane, Queensland 4000, Australia; 10 Estonian Genome Center, University of Tartu, Tartu 0794, Estonia; 11 Nathan Shock Center of Excellence in the Basic Biology of Aging, The Jackson Laboratory, Bar Harbor, Maine 04609, USA; 12 Department of Genetics, University Medical Center Groningen, University of Groningen, Groningen 9700RB, The Netherlands; 13 Institute of Human Genetics, Helmholz Zentrum München - German Research Center for Environmental Health, Neuherberg 85764, Germany; 14 Institute of Human Genetics, Technical University Munich, Munich 85540, Germany; 15 Neuroscience Division, Garvan Institute of Medical Research, Australia and Charles Perkins Centre and School of Molecular Bioscience, The University of Sydney, Sydney, New South Wales 2006, Australia; 16 Phoenix Epidemiology and Clinical Research Branch, National Institute of Diabetes and Digestive and Kidney Disease, National Institutes of Health, Phoenix, Arizona 85001, USA; 17 Institute for Molecular Medicine Finland FIMM, University of Helsinki, Helsinki 00131, Finland; 18 Department of Chronic Disease Prevention, National Institute for Health and Welfare, Helsinki 00131, Finland; 19 Department of Molecular Epidemiology, Leiden University Medical Center, Leiden 2300RC, The Netherlands; 20 Department of Genetics, Texas Biomedical Research Institute, San Antonio, Texas 78201, USA; 21 Division of Epidemiology, Human Genetics, and Environmental Sciences, School of Public Health, University of Texas Health Sciences, Center at Houston, Texas 77001, USA; 22 Institute of Molecular Medicine, University of Texas Health Sciences Center at Houston, Houston, Texas 77001, USA; 23 Department of Epidemiology and Prevention, Public Health Sciences, Wake Forest School of Medicine, Winston-Salem, North Carolina 27101, USA; 24 Computational Medicine Core, Center for Lung Biology, University of Washington, Seattle, Washington 98101, USA; 25 Section of Genetic Medicine, Institute for Genomics and Systems Biology, University of Chicago, Chicago, Illinois 60290, USA; 26 Program in Translational NeuroPsychiatric Genomics, Department of Neurology, Brigham and Women's Hospital, Harvard Medical School, Boston, Massachusetts 02108, USA; 27 Center of Human Development and Aging, New Jersey Medical School, Newark 07101, USA; 28 General Internal Medicine Section, Boston University, Boston, Massachusetts 02108, USA; 29 The Mathematical and Statistical Computing Laboratory, Center for Information Technology, National Institutes of Health, Bethesda, Maryland 20817, USA; 30 Department of Epidemiology, Erasmus Medical Center, Rotterdam 3000CA, The Netherlands; 31 Molecular Pathology, Institute of Biomedicine, University of Tartu, Tartu 0794, Estonia; 32 Department of Cardiovascular Sciences, University of Leicester, Leicester LE1, UK; 33 National Institute for Health Research Leicester Cardiovascular Biomedical Research Unit, Glenfield Hospital, Leicester LE1, UK; 34 Institute of Molecular and Cell Biology, Estonian Genome Center, University of Tartu, Tartu 0794, Estonia; 35 Institute of Epidemiologie II, Helmholtz Zentrum Muenchen, German Research Center for Environmental Health, Neuherberg 85764, Germany; 36 Research Unit of Molecular Epidemiology, Helmholtz Zentrum Muenchen, German Research Center for Environmental Health, Neuherberg 85764, Germany; 37 Institute of Clinical Diabetology, German Diabetes Center, Leibniz Center for Diabetes Research at Heinrich Heine University Düsseldorf, Düsseldorf 40593, Germany; 38 Division of Endocrinology and Diabetology, University Hospital Düsseldorf, Heinrich Heine University, Düsseldorf 40593, Germany; 39 Hannover Unified Biobank, Hannover Medical School, Hannover 30519, Germany; 40 Department of Psychiatry and Psychotherapy, Helios Hospital Stralsund, University Medicine Greifswald, Greifswald 17489, Germany; 41 Institute for Community Medicine, University Medicine Greifswald, Greifswald 17489, Germany; 42 Unit of Periodontology, Department of Restorative Dentistry, Periodontology and Endodontology, University Medicine Greifswald, Greifswald 17489, Germany; 43 Genetics of Complex Traits, University of Exeter Medical School, University of Exeter, Exeter EX2 5DW, UK; 44 Geriatric Unit, Azienda Sanitaria di Firenze, Florence 50123, Italy; 45 Centre for Genetic Origins of Health and Disease, The University of Western Australia, and Faculty of Health Sciences, Curtin University, Perth, Western Australia 9011, Australia; 46 Program in Medical and Population Genetics, Broad Institute of MIT and Harvard, Cambridge 02138, USA; 47 Divisions of Genetics and Rheumatology, Department of Medicine, Brigham and Women's Hospital and Harvard Medical School, Boston, Massachusetts 02108, USA; 48 Partners Center for Personalized Genetic Medicine, Boston, Massachusetts 02108, USA; 49 The Queensland Brain Institute, University of Queensland, Brisbane, Queensland 4000, Australia; 50 University of Queensland Diamantina Institute, University of Queensland, Princess Alexandra Hospital, Brisbane, Queensland 4000, Australia; 51 Department of Epidemiology, University of Michigan, Ann Arbor, Michigan 48103, USA; 52 Department of Biosciences, University of Helsinki, Helsinki 00100, Finland; 53 Department of Mental Health and Substance Abuse Services, National Institute for Health and Welfare, Helsinki 00100, Finland; 54 Wellcome Trust Sanger Institute, Hinxton, Cambridge CB4, UK; 55 Department of Public Health, Hjelt Institute, University of Helsinki, Helsinki 00100, Finland; 56 QIMR Berghofer Medical Research Institute, Brisbane, Queensland 4000, Australia; 57 Department of Psychiatry and Behavioral Sciences, Emory University School of Medicine, Atlanta, Georgia 30301, USA; 58 Max-Planck Institute of Psychiatry, Munich 80331, Germany; 59 Department of Internal Medicine, Wake Forest School of Medicine, Winston-Salem, North Carolina 27101, USA; 60 Department of Epidemiology, University of Washington, Seattle, Washington 98101, USA; 61 Cardiovascular Health Research Unit, Department of Medicine, University of Washington, Seattle, Washington 98101, USA; 62 Institute for Translational Genomics and Population Sciences, Los Angeles Biomedical Research Institute at Harbor-UCLA Medical Center, Torrance, California 90501, USA; 63 Department of Medical Statistics, Leiden University Medical Center, Leiden 2300RC, The Netherlands; 64 Department of Rheumatology, Leiden University Medical Center, Leiden 2300RC, The Netherlands; 65 Department of Clinical Epidemiology, Leiden University Medical Center, Leiden 2300RC, The Netherlands; 66 Division of Immunology, Department of Microbiology and Immunobiology, Harvard Medical School, Boston, Massachusetts 02138, USA; 67 Cardiovascular Health Research Unit, Department of Medicine, University of Washington, Seattle, Washington 98195, USA; 68 Cardiovascular Health Research Unit, Department of Epidemiology, University of Washington, Seattle, Washington 98195, USA; 69 Cardiovascular Health Research Unit, Department of Health Services, University of Washington, Seattle, Washington 98195, USA; 70 Group Health Research Institute, Group Health Cooperative, Seattle, Washington 98195, USA; 71 Department of Pathology, University of Vermont College of Medicine, Colchester, Vermont 98195, USA; 72 Division of Nephrology and Hypertension, Department of Medicine, Mayo Clinic, Rochester, Minnesota 55901, USA; 73 Computational Medicine, Institute of Health Sciences, Faculty of Medicine, University of Oulu, Oulu 90570, Finland; 74 Clinical Research Branch, National Institute on Aging, Baltimore, Maryland 21218, USA; 75 Division of Endocrinology, Children's Hospital Boston, Boston, Massachusetts 02108, USA; 76 Department of Genetics, Harvard Medical School, Boston, Massachusetts 02108, USA; 77 Laboratory of Neurogenetics, National Institute on Aging, National Institutes of Health, Bethesda, Maryland 20817, USA.; 78 Reta Lila Weston Institute and Department of Molecular Neuroscience, UCL Institute of Neurology, Queen Square, London WC1N 3BG, UK.; 79 Department of Medical and Molecular Genetics, King's College London, Guy's Hospital, London SE1 9RT, UK.; 80 Research Resources Branch, National Institute on Aging, National Institutes of Health, Bethesda, Maryland 20817, USA.; 81 Department of Neuroscience, Karolinska Institutet, Stockholm 10044, Sweden.; 82 Department of Neuropathology, MRC Sudden Death Brain Bank Project, University of Edinburgh, Edinburgh EH13, UK.; 83 Lymphocyte Cell Biology Unit, Laboratory of Immunology, National Institute on Aging, National Institutes of Health, Baltimore, Maryland 20817, USA.; 84 Department of Genetics, King Faisal Specialist Hospital and Research Centre 11564, Saudi Arabia.; 85 Brain Resource Center, Johns Hopkins University, Baltimore, Maryland 20817, USA.; 86 ITGR Biomarker Discovery Group, Genentech, South San Francisco, California 94101, USA.; 87 NICHD Brain and Tissue Bank for Developmental Disorders, University of Maryland Medical School, Baltimore, Maryland 2117, USA.

## Abstract

Disease incidences increase with age, but the molecular characteristics of ageing that lead to increased disease susceptibility remain inadequately understood. Here we perform a whole-blood gene expression meta-analysis in 14,983 individuals of European ancestry (including replication) and identify 1,497 genes that are differentially expressed with chronological age. The age-associated genes do not harbor more age-associated CpG-methylation sites than other genes, but are instead enriched for the presence of potentially functional CpG-methylation sites in enhancer and insulator regions that associate with both chronological age and gene expression levels. We further used the gene expression profiles to calculate the ‘transcriptomic age' of an individual, and show that differences between transcriptomic age and chronological age are associated with biological features linked to ageing, such as blood pressure, cholesterol levels, fasting glucose, and body mass index. The transcriptomic prediction model adds biological relevance and complements existing epigenetic prediction models, and can be used by others to calculate transcriptomic age in external cohorts.

## Abstract (web summary)

Ageing increases the risk of many diseases. Here the authors compare blood cell transcriptomes of over 14,000 individuals and identify a set of about 1,500 genes that are differently expressed with age, shedding light on transcriptional programs linked to the ageing process and age-associated diseases.

## Graphical abstract

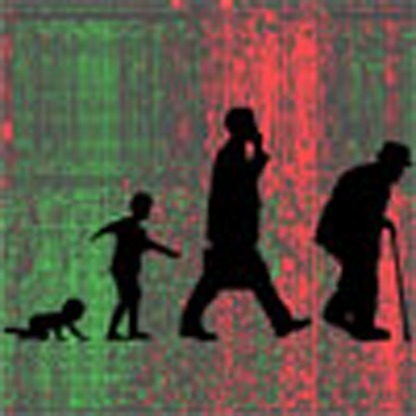

Chronological age is a major risk factor for many common diseases including heart disease, cancer and stroke, three of the leading causes of death. Although chronological age is the most powerful risk factor for most chronic diseases, the underlying molecular mechanisms that lead to generalized disease susceptibility are largely unknown. Genome-wide association studies (GWAS) have identified thousands of single-nucleotide polymorphisms (SNPs) associated with common human diseases and traits^1^,^2^. Despite this success, *APOE*, *FOXO3* and *5q33.3* are the only identified loci consistently associated with longevity^3^,^4^,^5^,^6^,^7^,^8^,^9^,^10^,^11^. Ageing has proven difficult to dissect in part due to its interactions with environmental influences (for example, lifestyle choices, diet and local exposures), other genetic factors, and a large number of age-related diseases^11^, making the individual factors difficult to detect.

Since studies in model organisms have shown that ageing is characterized by many alterations at the molecular, cellular and tissue level^12^, a transcriptome analysis might lend greater insight than a static genetic investigation. Therefore, the aim of this study was to exploit a large-scale population-based strategy to systematically identify genes and pathways differentially expressed as a function of chronological age. In contrast to the relatively invariable genome sequence, the transcriptome is highly dynamic and changes in response to stimuli. Previous gene expression studies in the context of ageing have primarily focused on model organisms^13^,^14^,^15^ or have been confined to specific ageing syndromes such as Hutchinson–Gilford progeria^16^. One report identified age-related expression modules across four separate data sets^17^, while other studies examined age-associated gene expression changes in relatively small cohorts^18^,^19^,^20^,^21^,^22^.

To our knowledge, we perform here the first large-scale meta-analysis of human age-related gene expression profiles with well powered discovery and replication stages. In addition, this is the first large-scale study testing the hypothesis that changes in gene expression with chronological age are epigenetically mediated by changes of methylation levels at specific loci. Finally, we take advantage of our large set of samples to build a transcriptomic predictor of age, and we compare our transcriptomic prediction model with the epigenetic prediction models of Horvath^23^ and Hannum *et al.*^24^.

We identified 1,497 genes that are differentially expressed with chronological age. These genes are enriched for the presence of potentially functional CpG-methylation sites in enhancer and insulator regions. Our transcriptomic age predictor complements the existing epigenetic prediction models, and can be used by others to calculate transcriptomic age in external cohorts.

## Results

### 1,497 genes differentially expressed with chronological age

The discovery stage included six European-ancestry studies (*n*=7,074 samples) with whole-blood gene expression levels for roughly half of the genes in the human genome (*n*=11,908 significantly expressed genes across different platforms). We identified 2,228 genes with age-associated expression in the discovery stage (*P*<4.2E−6) after adjusting for technical variables and confounding factors such as sex, cell counts and cigarette smoking (Supplementary Fig. 1A). The replication stage included 7,909 additional whole-blood samples, in which we replicated association results for 1,497 genes (*P*<2.2E−5). Discovery and replication results were highly correlated (*r*=0.972, Supplementary Fig. 1B) and complete results are shown in Supplementary Data 1. After meta-analysis of discovery and replication stages, the expression levels of 897 genes were negatively associated and 600 genes were positively correlated with chronological age. The top 50 most significantly associated genes are presented in Table 1.

### Transferability of ageing transcriptome signatures

To examine the generalizability of the results of our differential expression meta-analysis, we tested whether the 1,497 identified genes were also differentially expressed in relation to chronological age in other ancestry samples, in brain tissue, and in specific blood sub-cell-types (Supplementary Data 1). In Native Americans (*n*=1,457), 95% of the 1,497 genes were significantly expressed, and 71% (1,005 genes) were associated with chronological age (*P*<0.05). In Hispanic Americans (*n*=1,244), 40% of the 1,497 genes were significantly expressed, and 74% (440 genes) were associated with chronological age in the same direction (*P*<0.05). In African Americans (*n*=359), 99% of the genes were significantly expressed, and 27% (392 genes) were associated with chronological age in the same direction (*P*<0.05) (Supplementary Table 1).

In both types of brain tissue studies (cerebellum and frontal cortex, *n*=394), approximately 58% of the 1,497 genes were significantly expressed. Of these, 19% (163 genes) and 26% (229 genes) were associated with chronological age in the same direction (*P*<0.05) in cerebellum and frontal cortex, respectively (Supplementary Table 2, Supplementary Fig. 2, and Supplementary Table 3). Among the top 50 age-associated genes, three genes were associated with chronological age in all tissues: *SERPINE2*, *LDHB* and *BZW2* (*P*<0.05; Supplementary Data 2).

### Novel and known age-associated genes and pathways

**Figure 1:**
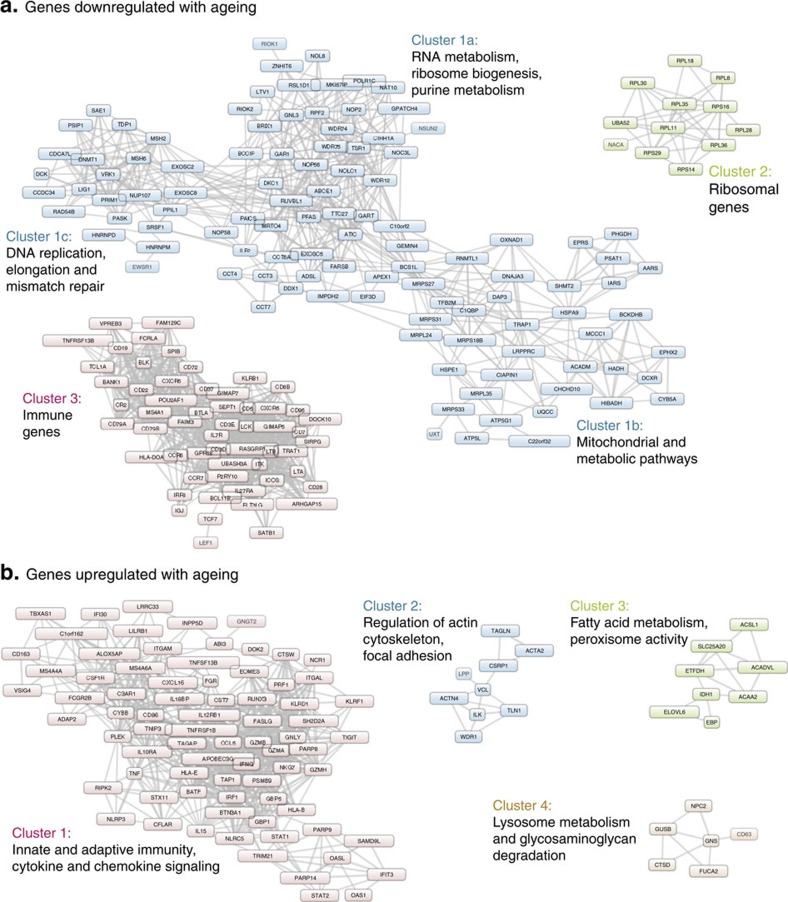
Pathway analysis on the clusters of co-expressed genes. We ran a co-functionality network analysis on 897 downregulated genes with age (negative effect direction) and 600 upregulated genes with age (positive effect direction) using GeneNetwork. With a correlation threshold of 0.7, we selected all clusters bigger than four genes and ran per-cluster pathway analyses using KEGG, Reactome, and GO-terms in WEBGESTALT. Benjamini & Hochberg FDR was used for multiple testing corrections. The significant threshold 0.05 after correction for multiple testing was applied. (**a**) Three clusters of downregulated genes with age and (**b**) four clusters of genes upregulated with age were enriched for functional pathways in KEGG, Reactome, and GO terms; the specific pathways are mentioned next to the (sub)cluster names.

To differentiate between changes caused by cell composition and other biological mechanisms, we clustered genes based on co-expression networks in GeneNetwork (see Methods) and performed pathway analysis on the clusters of co-expressed genes. Among the negatively age-correlated genes, three major clusters were identified (Fig. 1a, Supplementary Data 3A–M). The largest group (cluster #1, 109 genes) consisted of three sub-clusters enriched for: (1a) RNA metabolism functions, ribosome biogenesis and purine metabolism; (1b) multiple mitochondrial and metabolic pathways including 10 mitochondrial ribosomal protein (MRP) genes consistent with earlier ageing studies in mice, *Caenorhabditis elegans*^25^ and *Drosophila melanogaster*^26^,^27^,^28^; and (1c) DNA replication, elongation and repair, and mismatch repair^26^. The second cluster of negatively correlated genes (cluster #2, 57 genes) contained factors related to immunity; including T- and B-cell signalling genes, and genes involved in hematopoiesis. The third tight cluster (cluster #3) included 12 genes, of which 11 encoded cytosolic ribosomal subunits: 7 *RPL-*genes *(RPL8, RPL11, RPL18, RPL28, RPL30, RPL35* and *RPL36*), 3 *RPS*-genes (*RPS14, RPS16* and *RPS29*) and *UBA52* (ribosomal protein L40). The other gene of the cluster (#12) was *NACA,* a nascent polypeptide-associated complex alpha subunit. The protein encoded by the *NACA* gene forms the nascent polypeptide-associated complex (NAC), which binds to nascent proteins as they emerge from the ribosome^29^. Strikingly, the mRNA abundance of many genes encoding ribosomal subunits and mitochondrial ribosomal proteins were significantly associated with chronological age: 34 ribosomal genes were significantly associated, of which 33 were negatively correlated with chronological age (Supplementary Table 4), and 10 MRP genes were significantly negatively correlated with chronological age (Supplementary Table 5).

The positively age-correlated genes revealed four major clusters (Fig. 1b, Supplementary Data 3N–V): cluster#1 (77 genes): innate and adaptive immunity, cluster#2 (9 genes): actin cytoskeleton, focal adhesion, and tight junctions, cluster#3 (8 genes): fatty acid metabolism and peroxisome activity and cluster#4 (6 genes): lysosome metabolism and glycosaminoglycan degradation.

For both brain tissue studies, we checked the number (and %) of overlapping age-associated genes for the different functional clusters: 24 genes (11.7% of the genes expressed in cerebellum) and 33 genes (of the genes expressed in frontal cortex) of all pathway genes (278 genes) were associated with chronological age (Supplementary Tables 6 and 7). In cerebellum, the best replicating pathway was the positively age-correlated cluster #4: lysosome metabolism and glycosaminoglycan degradation. In frontal cortex, the best-replicating pathway was the positively age-correlated cluster #2: actin cytoskeleton, focal adhesion and tight junctions.

### Associations with prior ageing candidate genes

We investigated the intersection between genes significantly associated with chronological age in our study and candidate genes from previous human and animal studies (170 genes, see Supplementary Tables 8 and 9). Thirty-three of the 170 candidate genes were significantly associated with chronological age in our whole blood meta-analysis, including members of the mTOR/FOXO pathways (*FOXO1, VEGFB, EIF4G3, SREBF1, STAT3 and RPS6KB1*)^30^, DNA repair (*ATM*)^31^, and prior multispecies candidates (*LDHB, IGJ, IRF8*and *FCGR1A*). Twenty-eight of the 33 significant age-associated genes (∼85%) have the same expression directionality in our CHARGE meta-analysis as previously reported in a variety of studies in humans and other model organisms.

Premature ageing syndrome genes *ATM* (ataxia-telangiectasia), *DKC* (dyskeratosis congenita) and *WRN* (werner syndrome) all exhibited lower transcript abundance in older individuals, concordant with loss-of-function alterations in disease-related mutations. On the basis of the co-expression analyses, these genes clustered together with genes encoding proteins involved in DNA and RNA metabolism, DNA repair, and purine/pyrimidine metabolism. The Hutchinson–Gilford progeria gene *LMNA* showed higher mRNA levels in the elderly, consistent with earlier findings in muscle^32^, and clustered with actin remodelling genes.

### Methylation association patterns for top age-associated loci

Given the possible role of the methylome in ageing, we investigated whether age-associated methylation accompanied age-associated expression for the 1,497 age-associated genes. We analysed methylation of 135,230 CpG sites (regions of DNA where a cytosine nucleotide occurs next to a guanine nucleotide) in or near (±250 kb) the age-associated genes in whole blood or peripheral blood mononuclear cells (PBMCs) from seven cohorts (*N*=3,073). We chose CpGs in a 250 kb vicinity because earlier studies have shown that methylation can regulate gene expression levels at this distance^33^, and that long-range enhancer activities are present and actively regulate gene expression at a wide scale^34^. We observed significant associations between methylation and chronological age for 31,331 CpG sites, and between expression and methylation for 12,280 CpG sites, based on a conservative Bonferroni threshold (*P*<3.7E−7) (top results for each gene in Supplementary Data 4). In all, 1,248 of the 1,497 age-associated genes (83%) had ≥1 significant mediating CpGs and the number of significant mediating CpGs per gene ranged from 1 to 154 (Supplementary Data 4).

**Figure 2:**
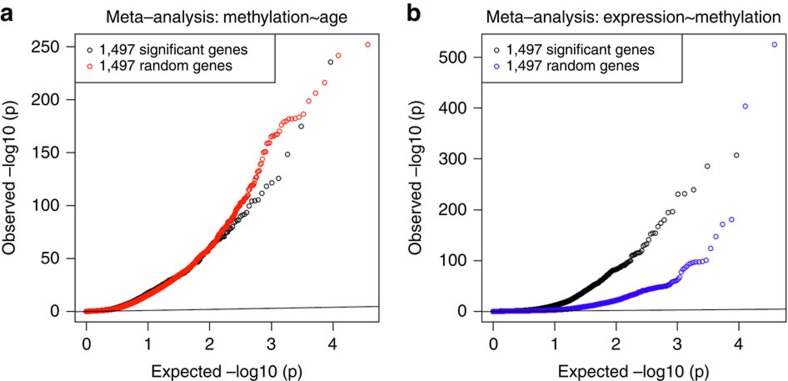
Age-associated genes are enriched for the presence of potentially functional methylation sites. (**a**) Quantile–quantile (QQ) plot of the observed *P*-values (−log10P) for the methylation–age associations. The plot in black shows pvalues from the 1,497 significant age-associated genes, whereas the plot in red shows pvalues for 1,497 random genes. We do not see enrichment for the 1,497 age-associated genes. (**b**) QQ plot of the observed *P*-values (−log10P) for the expression–methylation associations. The plot in black shows *P* values from the 1,497 significant age-associated genes, whereas the plot in blue shows pvalues for 1,497 random genes. The age-associated genes are enriched for CpG methylation sites that associate with gene expression levels.

To test whether the age-associated genes were enriched for nearby CpG methylation sites associated with chronological age or expression, we performed a similar analysis for a set of 1,497 randomly selected genes matched for similar gene length and mean whole blood expression (see Methods and Supplementary Fig. 3A–D). Compared to the set of random genes, age-associated genes had only mild enrichment for CpG methylation sites associated with chronological age (Fig. 2a; odds ratio (OR)=1.04; 95% confidence interval (CI)=1.02–1.06; *P*=7.9E−5), but strong enrichment for CpG methylation sites associated with expression (Fig. 2b; OR=2.68; 95% CI=2.58–2.78; *P*<1E−300). This pattern was consistent across all cohorts (Supplementary Fig. 4) and within subsets of CpG methylation sites annotated to specific biological features (that is, enhancer regions, promoter regions, CpG islands and so on.) (Supplementary Fig. 5), and was robust to the entire range of significance thresholds (see Methods). This is consistent with a scenario where many methylation sites associate with chronological age, but only those with regulatory potential lead to altered transcript expression with chronological age.

We used Sobel tests (see Methods) for all CpG methylation sites to investigate whether the observed patterns could potentially reflect a methylation-mediated relationship between chronological age and transcript levels. In total, 1,248 of the 1,497 age-associated genes (83%) had ≥1 CpG site with a significant Sobel test after Bonferroni adjustment for the number of CpGs tested (Supplementary Data 4). These potentially mediating CpG sites were less likely to reside in CpG islands (OR=0.28; 95% CI=0.26–0.30; *P*<1E−300) or in promoters (OR=0.38; 95%CI=0.36–0.40; *P*<1E−300) and more likely to be located in enhancers (OR=2.29; 95%CI=2.17–2.41; *P*=2.7E−188) and insulators (OR=1.44; 95% CI=1.23–1.67; *P*=6.6E−6), compared with non-mediating CpGs within 250 kb of age-associated genes (Supplementary Fig. 6). This pattern is again consistent with the mediation of age-associated transcripts by age-associated methylation of CpG sites with specific regulatory roles.

### Transcriptomic age prediction as a surrogate biomarker

**Figure 3:**
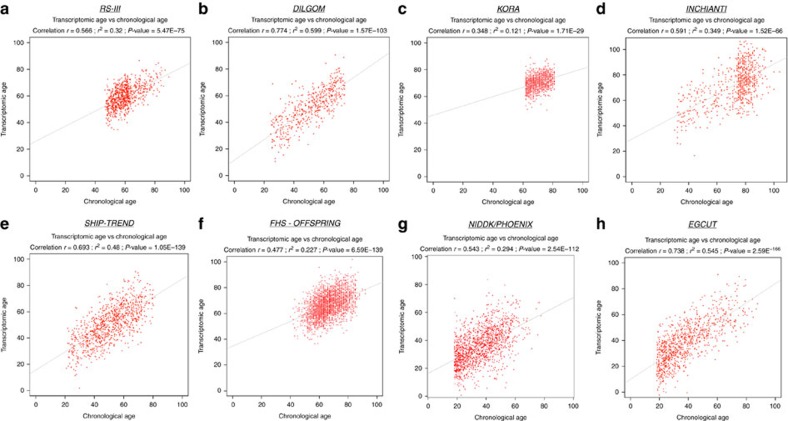
Transcriptomic age versus chronological age. This figure represents the correlations between chronological age (*x* axis) and transcriptomic age (*y* axis) in eight different cohorts: (**a**) RS-III, (**b**) DILGOM, (**c**) KORA, (**d**) InCHIANTI, (**e**) SHIP-TREND, (**f**) FHS-OFFSPRING, (**g**) NIDDK/PHOENIX and (**h**) EGCUT. Transcriptomic age was calculated using a cohort-specific prediction formula and the measured gene expression levels of 11,908 genes. The correlation between chronological age and transcriptomic age was significant in all cohorts (*P*<2E−29).

**Figure 4:**
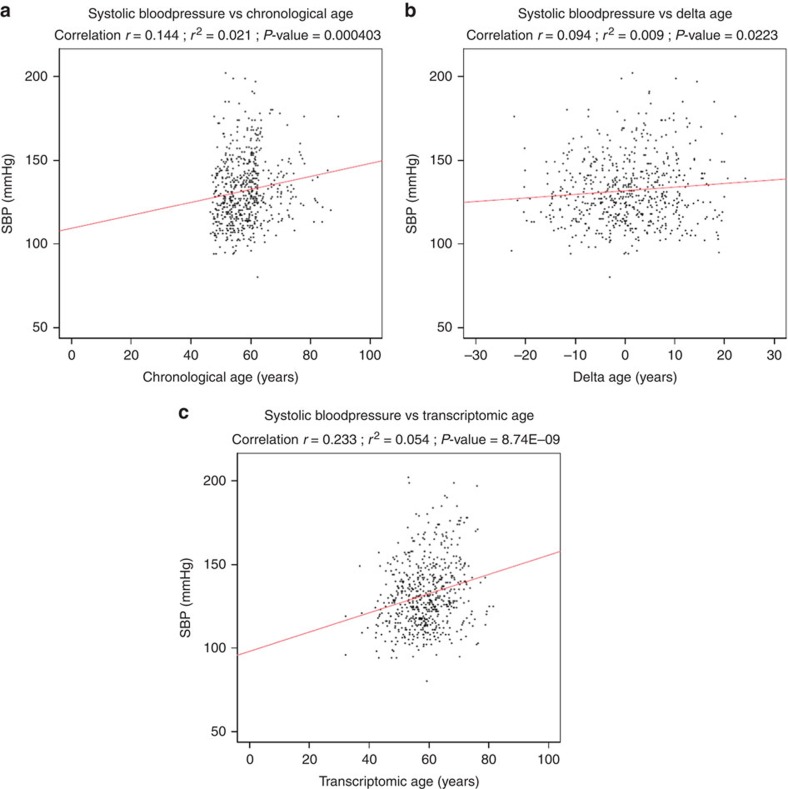
The added value of the transcriptomic predictor. To show the added value of the transcriptomic predictor, we choose one biological ageing phenotype systolic blood pressure (SBP), and plotted its correlation with chronological age (**a**), delta age (**b**) and the transcriptomic age (**c**) in the Rotterdam Study (*n*=597 samples with SBP data available). Delta age represents the difference between chronological age and transcriptomic age. SBP was plotted on the *y* axis, and the age-related values were plotted on the *x* axes. SBP was significantly associated with chronological age (*P*=4.0E−04), but SBP was even stronger associated with transcriptomic age (calculated with a cohort-specific prediction formula based on gene expression levels) (*P*=8.7E−09), Therefore, the transcriptomic predictor adds value over chronological age alone. Other biological ageing phenotypes showed the same pattern.

All 11,908 discovery genes were used to build a predictor for age using a leave-one-out-prediction meta-analysis (see Methods). For each cohort in turn, we left out that cohort as the validation sample and re-ran the discovery meta-analysis on the other cohorts to avoid overlap between the discovery and validation sample (Supplementary Data 5A). The difference between the predicted transcriptomic age and chronological age (delta age) may be a reflection of altered biological age (see Methods). The correlation between chronological age and transcriptomic age was significant in all cohorts (*P*<2E−29; Fig. 3a–h). The average absolute difference between predicted age and chronological age was 7.8 years (*n*=8,847 samples, Supplementary Table 10). A positive delta age, interpreted as reflecting more rapid biological ageing, was consistently associated with higher systolic and diastolic blood pressure, total cholesterol, HDL cholesterol, fasting glucose levels and body mass index (BMI) (Table 2, Supplementary Table 11). All analyses were adjusted for chronological age, and after adjustment for BMI all phenotypes remained associated in the same direction (Table 2, Supplementary Table 12). For systolic blood pressure, the added predictive value of the transcriptomic predictor over chronological age is shown for the Rotterdam Study (Fig. 4a–c). Other phenotypes showed the same pattern.

We compared our transcriptomic predictor with two already published epigenetic predictors of age of Horvath^23^ and Hannum *et al.*^24^ in 1,396 individuals from the KORA study and the Rotterdam Study, all having gene expression levels and methylation data available. The transcriptomic predictor was less strongly correlated with chronological age than the two epigenetic predictors (Supplementary Fig. 7), which can be explained by the different data types used: we used gene expression data instead of DNA methylation data.

Transcriptomic age and epigenetic age (both Hannum and Horvath) were positively correlated, with *r*^2^ values varying between 0.10 and 0.33 (Supplementary Fig. 7). Interestingly, all three age predictors were associated with different ageing phenotypes (Supplementary Tables 13 and 14), that is, the transcriptomic predictor was significantly associated with systolic blood pressure, waist-hip-ratio, and smoking; the epigenetic Horvath predictor was associated with waist-hip-ratio only; and the epigenetic Hannum predictor was associated with fasting glucose, waist-hip-ratio and smoking (all analyses were adjusted for chronological age, sex and BMI). By adding two predictors into one formula (one transcriptomic predictor and one epigenetic predictor), both predictors added value (significant effect) to the phenotype associations, that is, for waist-hip-ratio in KORA (explained variance transcriptomic predictor=0.015%, Horvath predictor=0.005%, Hannum predictor=0.006%; transcriptomic+Horvath=0.017% and transcriptomic+Hannum=0.016%) (Supplementary Tables 15 and 16).

## Discussion

Age-associated changes in gene expression levels point towards altered activity in defined age-related molecular pathways that may play vital roles in the mechanisms of increased susceptibility to ageing diseases. In contrast to earlier, smaller studies^17^,^18^,^19^,^20^,^21^ of human age-related molecular differences, we detected and replicated 1,497 age-associated genes in 14,983 individuals of European ancestry. In addition, many of our associations were generalized across different ancestries and multiple cell and tissue types. Because we had much smaller sample sizes for both brain tissue (*n*=394) and the other ancestry groups (1,244 Hispanic Americans, 1,457 Native Americans, and 359 African Americans), we used a nominal *P*-value threshold (*P*<0.05) in these specific sub-analyses. Larger sample sizes will ultimately be needed to fully understand the transferability of the ageing-transcriptome signatures.

A potential limitation of our study is that we relied on a linear regression model to identify age-associated genes. A linear model assumes constant change over age, which may not be always correct in biological processes that stretch over several decades (adulthood). A recent study demonstrated that a quadratic regression model has a higher statistical fit to cross-sectional gene expression datasets over linear models^35^. Although we chose to apply a linear regression model in our study, we recognize that more complex models could be investigated in future studies.

Our human age-expression and pathway enrichment analysis results were consistent with known ageing mechanisms including dysregulation of transcription and translation, metabolic function, DNA damage accumulation, immune senescence, ribosome biogenesis and mitochondrial decline. Houtkooper *et al.*^25^, McCarroll *et al.*^26^ and Landis *et al.*^27^ highlighted the key role of mitochondria in ageing and longevity in model organisms. Mitochondria regulate a multitude of different metabolic and signalling pathways and also play an important role in programmed cell death^36^. The number of mitochondria decreases and their capacity to produce energy is reduced with chronological age^37^,^38^,^39^. Consistent with these reports, a large number of mitochondrial ribosomal proteins (*MRPL24, MRPL3, MRPL35, MRPL45, MRPS18B, MRPS26, MRPS27, MRPS31, MRPS33* and *MRPS9*) showed lower expression at higher chronological age in our study, supporting the hypothesis that age-dependent mitochondrial dysfunction plays a causal role in human ageing.

The large immune function associated clusters (cluster #2 and cluster #1 of the negatively and positively correlated genes, respectively) reflect immune senescence. The relative abundance of immune cells in whole blood shifts with ageing, with naive T cells decreasing and highly differentiated effector and memory T cells increasing with chronological age^28^,^40^,^41^,^42^,^43^,^44^. Consistent with immune senescence, the mRNA abundance of the chemokine receptor *CCR7* and cell differentiation antigens *CD27* and *CD28* was lower in older individuals (*P*=1.0E−208, *P*=2.8E−162, and *P*=5.8E−59). Notably, these results were consistent in many of the blood sub-cell-types. For example, *CCR7* was lower in older individuals across multiple cell types including CD4+ cells (*P*=1.0E−08), CD8+ cells (*P*=3.0E−15), CD14+ cells /monocytes (*P*=8.5E−3), and PBMCs (*P*=3.0E−3). This suggests that genes in the immune associated clusters reflect a biological function related to a more general ageing phenotype , at least in multiple immune cell types, and are not solely accountable to cell-count differences. We also note that cell subset classification is to a greater or lesser extent artificial, reflecting our current ability to distinguish cells based on specific small sets of available markers. Accepted subpopulation of cells can often be further broken down into additional subgroups as the tools for such classification become more sophisticated. The analysis of unfractionated cell populations (such as our study) adds a layer of complexity to the interpretation, but is not necessarily less informative than the analysis of marker defined subpopulations.

Aside from the immune clusters, we identified and newly emphasized pathways associated with human ageing, for example, glycosaminoglycan degradation and actin remodelling. These pathways have previously been implicated in life span regulation of the model organisms *C. elegans* and *D. melanogaster*^45^,^46^,^47^. Glycosaminoglycans (GAGs) influence cell migration, proliferation and differentiation and play a role in wound healing^48^,^49^. Impaired degradation of GAGs in extreme lysosomal storage disorders lead to chronic, progressive effects on a variety of organs and physiologic systems^50^. Tissue repair and regeneration are known to be impaired in the elderly and inhibition of GAG degradation may be therapeutic in these contexts^51^. Our findings suggest GAG degradation as a candidate mechanism for the age-associated changes. The actin cytoskeleton is a critical structural element in eukaryotic cells that is crucial in mediating cell responses to both internal and external signals in yeast^52^. Actin dynamics have clearly been linked to yeast replicative ageing through both reactive oxygen species-mediated apoptosis and through selective sequestration of healthy mitochondria to new daughter cells during cell division^52^,^53^. Our pathway analysis indicates that the actin cytoskeleton may be similarly important in human ageing. While much prior effort in targeting actomyosin dynamics has been aimed at cancers, recent studies indicate that targeted modulation of these systems could also have benefits in immune-mediated pathologies^48^.

In addition to these novel candidate pathways, our 1,497 age-associated genes contain genes in many pathways known to be associated with ageing. Beyond the immune-related pathways, we confirm an age-associated role for mitochondrial function^54^, metabolic function^12^, ribosome biogenesis^55^, DNA replication, elongation and repair^56^,^57^, focal adhesion^58^ and lysosome metabolism^59^, and suggest a number of new potential age-related targets within these pathways, including *TTC27* (ribosome biogenesis); *CCDC34* (ribosomal cluster); *ARHGAP15, DOCK10, FAM129C, FCRLA, GIMAP7* and *VPREB3* (T- and B-cell signalling genes and genes involved in haematopoiesis); *GZMH, SAMD9L* and *TAGAP* (innate and adaptive immunity). Of note, overexpression of the full-length *ARHGAP15* protein in COS-7 and HeLa cells resulted in an increase in actin stress fibres and cell contraction, relating the newly ageing emphasized actin remodelling pathway and the focal adhesion pathway in ageing to immune cell changes^60^. Thus, by using co-expression networks, we identified new genes and pathways that are likely important in human ageing, opening new avenues of enquiry for future studies.

Age-related epigenetic changes have recently been examined including a large study combining data across 7,844 non-cancer samples from 82 individual data sets to define a set of age-methylation clock genes. Only 35 of our 1,497 age-related genes were found among the genes harbouring the 353 age-methylation clock CpG sites reported by Horvath^23^, suggesting that our age-associated genes may not be particularly enriched for age-associated CpG methylation sites. To test this formally, we analysed the DNA methylation sites (CpG sites only) within 250 kb (upstream and downstream) of all 1,497 age-associated genes, as well as a comparison set of 1,497 randomly chosen unassociated genes. We observed that the genes exhibiting age-associated transcript levels in blood are much more likely than other genes to harbour CpG methylation sites that associate with expression levels, but are not substantially more likely to harbour methylation differences in close CpG sites associated with chronological age. These results suggest that genes showing age-related expression differences are characterized primarily by the presence of nearby CpG sites with regulatory potential, rather than by the presence of age-associated CpG methylation sites, which are abundant everywhere in the genome. A limitation of our study is that we used the Illumina Infinium HumanMethylation450K BeadChip Array for measuring methylation levels: this array queries only 1.6% of all CpGs in the genome and the CpG selection is biased towards CpG islands. In addition, we did not examine non-CpG methylated sites, which have recently been suggested to play a role in regulating gene expression as well^61^. Other techniques—whole-genome bisulfite sequencing^62^ and methylC-capture (MCC) sequencing^63^, for example—have definite technical advantages (higher resolution and no CpG island selection bias), but these have currently not been applied to a large number of samples.

Although the CpG selection on the methylation array is biased towards CpG islands, the CpG sites for which methylation was associated with both expression and chronological age were strongly enriched for enhancer activity. This is consistent with the concept that methylation at enhancers is more variable and may regulate gene expression in development^64^ and/or in environmental responses, while promoter methylation is comparatively stable. Interestingly, the age- and expression-associated CpGs were also enriched at insulators, which function to block the communication between an enhancer and a promotor, thereby preventing inappropriate gene activation. Taken together, these results suggest that the age-associated genes reported here may be regulated by methylation of CpG sites in specific functional regions, and that studying both methylation and expression as potential joint effectors of the ageing process may significantly improve the prediction of age and identification of novel age-related genes and pathways.

Using gene expression levels as a predictive biomarker indicated that individuals having higher predicted than chronological age also have clinical features consistent with an older age, such as higher blood pressure and total cholesterol levels. Developing a strongly predictive gene expression set as a biomarker panel has clinical potential to identify subjects at risk for early biological ageing, and provide a tool for targeting susceptible individuals for early intervention. It remains to be seen whether the transcriptomic age can serve as a surrogate marker to predict age-associated decline in other tissues. Therefore, the development of a robust transcriptomic predictor for age will require independent and prospective validation across different tissues.

We observed that both the transcriptomic predictor and the epigenetic predictors were significantly associated with a number of phenotypes, but that the pattern of association differed among the predictors. Therefore, the transcriptomic age and the epigenetic age should be combined to obtain the optimal biological age prediction. A general transcriptomic prediction formula has been calculated that is freely available (Supplementary Data 5B). These results suggest that the biological mechanisms behind the transcriptomic and the epigenetic predictors are different. The exact mechanism of these differences need further examination in larger sample sizes and subgroup analysis were different diseases are studied. In addition, the predictors need to be evaluated for their prognostic value. In conclusion, gene expression levels are likely to become a valuable addition to evolving indicators of age based on epigenetic and telomeric age predictors. Ideally, a combination of transcriptomic, epigenetic and telomeric elements could further improve and refine age prediction.

In conclusion, we have identified a compendium of genes and pathways associated with human chronological age. By leveraging transcriptional information across large, multiethnic cohorts, different tissue types and genomic repositories, we captured an unprecedented overview of the complex and temporally dynamic biological pathways orchestrating the ageing process. Our list of genes should provide a rich trove of data for future ageing studies. While the pursuit of an anti-ageing panacea in humans remains a distant goal, our work has generated new biological hypotheses and will serve as a roadmap for future studies aimed at translating findings into treatment strategies for age-related diseases.

## Methods

### Study design

We performed a differential expression meta-analysis in 7,074 human peripheral blood samples from six independent cohort studies, including EGCUT (*n*=1,086), FHS—2nd generation (*n*=2,446), INCHIANTI (*n*=698), KORA (*n*=993), ROTTERDAM STUDY (*n*=881), and SHIP-TREND (*n*=970; Supplementary Table 17). Gene expression data for each dataset was obtained using either PAXGene (Becton Dickinson) or Tempus Tubes (Life Technologies), followed by hybridization to Illumina Whole-Genome Expression BeadChips (HT12v3 or HT12v4) or Affymetrix Human Exon 1.0 ST GeneChips.

We replicated the significantly associated transcripts in 7,909 peripheral blood samples from seven independent cohort studies, including BSGS (*n*=862), DILGOM (*n*=512), FEHRMANN (*n*=1,191), FHS—3rd generation (*n*=3,180), GTP (*n*=359), HVH (*n*=121 on the Illumina HT12v3 platform and *n*=227 on the Illumina HT12v4 platform), and NIDDK/PHOENIX (*n*=1,457) (Supplementary Table 18). Gene expression data for these datasets was also obtained using either PAXGene (Becton Dickinson) or Tempus Tubes (Life Technologies), followed by hybridization to Illumina Whole-Genome Expression BeadChips (HT8v2, HT12v3, or HT12v4 arrays) or Affymetrix Human Exon 1.0 ST GeneChips.

We generalized the significantly replicated transcripts in 4,644 samples with other tissue types, including: CD4+ cells of EGCUT (*n*=302) and a Boston sample (*n*=213), CD8+ cells of EGCUT (*n*=299), CD14+ cells (or monocytes) of a Boston sample (*n*=213) and MESA (*n*=354), LCLs of GENOA (*n*=869), lymphocytes of SAFHS (*n*=1,244), PBMCs of GARP (*n*=134) and PMBC-MS (*n*=228), and brain tissue (cerebellum and frontal cortex) of NABEC-UKBEC (*n*=394) (Supplementary Table 19). Gene expression data of these data sets was obtained by tissue specific RNA isolation and hybridization to Illumina Whole-Genome Expression BeadChips (WG6v1, HT12v3 or HT12v4), Affymetrix Human Exon Arrays, or Affymetrix Human Gene Arrays.

The study outline is summarized in Supplementary Fig. 8. The study populations, the RNA isolation methods, the amplification and labelling methods and the array types used for each study are described in the Supplementary Methods. The covariates used in each study are presented in Supplementary Tables 17–19.

### Phenotype

Chronological age was defined as the length of time in years between birth and blood draw, using two decimals. Detailed descriptions of the chronological age distributions, fasting status and the available covariates from the participating cohorts are presented in Supplementary Tables 17–19 and Supplementary Fig. 9A–V.

### Illumina pipeline: gene expression probes and normalization procedure

The different Illumina platforms used by the different cohorts share a large number of probes with identical 50-mer probe sequences. Therefore, we harmonized the probes across the HT12-v3 and the HT12-v4 platforms by determining the probe sequences from the different annotation files for each platform; renumbering the probes on the basis of unique probe sequences. In total, we identified 56,330 unique Illumina probes (11,453 probes measured only on the HT12-v3 platform, 7,529 probes measured only on the HT12-v4 platform, 37,348 probes measured on both platforms). Genes were declared significantly expressed in the discovery data when (1) the detection *P*-values calculated by GenomeStudio were <0.05 in >10% of all discovery samples and (2) the probes were measured in at least two cohorts. This resulted in 23,170 transcripts considered as being significantly expressed in our Illumina discovery; these transcripts code for 15,639 well characterized unique genes. 3,484 genes have more than one Illumina probe on the HT12 platform. Illumina gene expression data was quantile normalized to the median distribution and subsequently log2-transformed. The probe and sample means were centered to zero.

### Affymetrix pipeline: gene expression probes and normalization procedure

The Affymetrix platform generated CEL files, containing both gene-based and exon-based expression levels. We used the gene-based expression levels and normalized the data using Affymetrix Power Tools: probes with RLE mean values >3.0 (range 1.34–12.71) were considered to be significantly expressed. This resulted in 16,798 well characterized unique genes in the Affymetrix discovery. Samples with all probeset RLE means > 0.7 were defined as outliers and excluded from further analysis. A genetic expression SNP analysis was undertaken to locate mislabeled samples and reidentify them where possible with high confidence. After exclusions and reidentification, the RMA normalization was repeated.

### Differential expression with chronological age

All Illumina studies ran a least squares linear regression model (lm) using the normalized and standardized gene expression values as dependent variables, chronological age as an explanatory variable and with adjustments for the potential confounders: sex (factor), fasting and smoking status (both factors), plate origin (factor), RNA quality (RIN/RQS) and cell counts (number of granulocytes, lymphocytes, monocytes, erythrocytes and platelets), so:


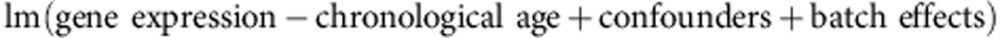


The Affymetrix cohort ran a multivariate stepwise PC regression, using the normalized and standardized gene expression values as dependent variables, chronological age as an explanatory variable, and the significant technical covariates: all_probeset_mean, all_probeset_stdev, neg_control_mean, neg_control_stdev, pos_control_mean, pos_control_stdev, all_probeset_rle_mean, all_probeset_mad_residual_mean, RNA quality (RIN), and RNA processing batch. Batch was included in modelling as a random factor while all others were fixed factors.

### Meta-analysis of significantly expressed genes

We ran four separate meta-analyses: one for the studies using the Illumina platforms in the discovery phase, one for the Illumina discovery studies plus the FHS Affymetrix discovery results, one for the replication sample combined, and one for the discovery samples plus replication samples for validated results to re-rank the final results list. For these meta-analyses, we used a sample size weighted meta-analysis based on *P*-values and the direction of the effects; using *P*-values, a *Z*-statistic characterizing the evidence for association was calculated. The *Z*-statistic summarized the magnitude and the direction of the effect. An overall *Z*-statistic and *P*-value was calculated from the weighted sum of the individual statistics. Weights were proportional to the square-root of the number of individuals examined in each sample and standardized such that the squared weights sum to 1.

We calculated the *Z*-scores and *P*-values using the Meta-Analysis Tool for genome-wide association scans (METAL)^65^. METAL is a flexible and computationally efficient command line tool that was developed for meta-analyzing GWAS studies, but can easily be adapted to gene expression studies. Because we are dealing with gene expression levels and not SNPs, we changed the SNPID column to probe IDs and gave all probes a minor allele A and a major allele G, a minor allele frequency=0.10, and a + strand. For the positions, the probe chromosomes and the midpoint position of the probes were used. Sample sizes, effect directions, and *P*-values were extracted from the linear model results files. We extensively tested what input parameters to use for meta-analysing gene expression data. By using similar allele names, allele frequencies, and allele strands for all cohorts, we forced METAL to use the default meta-analysis approach. We tested an inverse variance weighted meta-analysis (using the effect size estimates and the standard errors), and found that our METAL meta-analysis results were identical to the meta-analysis results using the R package Meta.

*Meta-analysis of discovery samples*. To calculate which genes are significantly associated with chronological age, we ran a sample size weighted meta-analysis based on *P*-values and the direction of the effects of the results of the Affymetrix and the Illumina meta-analyses. Combining the 16,798 Affymetrix probes and the 15,639 Illumina probes, these platforms have 11,908 genes significantly expressed in whole blood samples in common.

*Replication phase*. Genes with a *P*-value <4.20E−6 (0.05/11,908 genes tested) were considered transcriptome-wide significantly associated with chronological age. We replicated these findings in an additional 8,009 samples (Supplementary Table 18). Replication cohorts used the same analysis plan and R-scripts as the discovery phase, however, some covariates were not available in these cohorts and ethnicities could be different than European-ancestry.

*Meta-analysis of the replication cohorts*. We meta-analysed the summary statistics of the replication cohorts using METAL. Genes were considered significantly replicating if *P*<2.23E−5 (0.05/2,238 genes tested) and the overall *Z*-score was in the same direction as the overall *Z*-score of the discovery meta-analysis.

*Meta-analysis of discovery and replication cohorts*. We additionally performed a meta-analysis based on the summary statistics of all discovery and all replication cohorts and obtained two-sided *P*-values.

### Generalization phase

To see whether our findings are specific for whole blood, we tried to generalize our significantly replicating transcripts in samples of other tissue types, including CD4+ cells, CD8+ cells, CD14+ cells (monocytes), LCLs, lymphocytes, PBMCs and brain tissue (both cerebellum and frontal cortex; Supplementary Table 19). If we had data of one tissue type of more than one cohort, then we ran a meta-analysis based on the summary statistics of both cohorts. Because sample sizes of these tissue types were very small, we considered *P*-values <0.05 (with an identical effect direction) sufficient to document generalization of the effect.

### Pathway analysis of significant genes

We used WEBGESTALT ( http://bioinfo.vanderbilt.edu/webgestalt/analysis.php) and GeneNetwork ( http://genenetwork.nl:8080/GeneNetwork/pathway_network.html) for pathway analysis of age-associated transcripts. First, we ran the co-functionality network analysis separately on 897 down-regulated genes and 600 up-regulated genes, using a correlation threshold of 0.7. Of 897 downregulated genes, 192 formed cluster groups at this threshold, and of 600 upregulated genes, 114 formed cluster groups. We next re-ran the co-expression cluster analysis on these 192 and 114 genes, using a correlation threshold of 0.65 to see if small clusters could be merged together if a lower co-expression threshold was applied. We selected clusters with five and more genes for pathway analysis; in total 178 and 100 down- and upregulated genes respectively. On the basis of the clustering analysis, we performed per-cluster pathway analysis. Pathways were selected using KEGG, Reactome and GO-terms. In WEBGESTALT Benjamini & Hochberg FDR was used for multiple testing corrections. The significant threshold 0.05 after correction for multiple testing was applied.

### Analysis of chronological age, methylation and expression

For 3,073 blood samples with methylation data available from the Illumina 450 K array, we analysed methylation for CpG sites within 250 kb of the 1,497 genes identified in the differential expression meta-analysis. For this analysis we performed a new meta-analysis of samples from seven cohorts including EGCUT (*n*=82), InChianti (*n*=485), KORA (*n*=735), Rotterdam Study (*n*=726), BSGS (*n*=610), GTP (*n*=315) and GARP (*n*=120); all samples were derived from whole-blood except for GARP (PBMCs). After filtering (to remove non-specific probes and probes with SNPs in the probe target as documented by Price *et al.*^66^), 135,230 CpG sites within 250 kb of the 1,497 age-associated genes were eligible for analysis.

Within each cohort, we fit two linear regression models where we considered as our dependent variable either standardized gene expression values for a particular gene or methylation β-values, which are measures of the proportion of DNA methylated within a sample, for a particular CpG site. In Model 1, we regressed methylation β-values on chronological age. In Model 2, we regressed gene expression on both methylation and chronological age. In both models we adjusted for the following potential confounders as available in each cohort: sex, fasting and smoking status (both modelled as categorical variables or factors), and cell counts (number of granulocytes, lymphocytes, monocytes, erythrocytes and platelets). In Model 1, where methylation was the dependent variable we adjusted for chip and row on chip (both as factors). In Model 2, where the dependent variable was gene expression we adjusted for plate origin (factor) and RNA quality (RIN/RQS). For each of the age-associated genes, we fit these models separately for each CpG site within 250 kb (upstream or downstream) of the gene.

To combine results from these models across cohorts, we performed a sample size weighted meta-analysis based on the *t*-statistics from these models. For each model, we calculated a *Z*-score as the weighted sum of *t*-statistics across the seven cohorts. As above, weights were proportional to the square-root of the number of individuals analysed in each cohort and selected such that the squared weights sum to 1. To test for mediation of the age-expression relationship by methylation of a particular CpG site, we used the *Z*-scores from Model 1 and Model 2 to perform a Sobel test^67^, such that our Sobel *Z*-score was equal to:


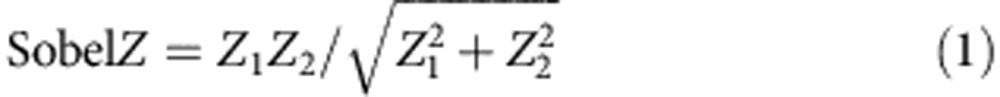


where *Z*_1_ is the meta-analysis *Z*-score from the association between methylation and chronological age in Model 1, and *Z*_2_ is the meta-analysis *Z*-score from the association between expression and methylation, adjusted for chronological age, in Model 2. To assess overall significance for each model (Model 1, Model 2 and the Sobel test), we used a Bonferroni-adjusted α-level of .05/135,230=3.70 × 10^−7^ for all CpG sites tested. To assess whether sites in each gene were significant, we assessed Bonferroni significance for each gene according to the number of CpG sites tested in that gene.

To test whether the genes were enriched for CpG sites associated with chronological age in Model 1, or CpG sites associated with expression in Model 2, we performed similar analyses on a set of 1,497 random genes. We chose these genes by first selecting the 5,000 least-associated genes from the original age-expression analysis. We then used the optmatch R package^68^ to select a subset of 1,497 random genes that were well-matched to the 1,497 age-associated genes in terms of gene length (bp) and the log of mean expression in whole blood. By doing this, we obtained a set of 1,497 random genes that were similar to the 1,497 age-associated genes in distributions of gene length, mean expression, and number of CpG sites within 250 kb (Supplementary Fig. 3A–D). We then performed the meta-analysis for Models 1 and 2 for all eligible CpG sites (after filtering to remove sites with probes that were non-specific or harboured genetic variants) within 250 kb of these genes. We used Fisher's exact test to test whether there was an increased proportion of significant (*P*<*α*) CpG sites in each model in the age-associated genes compared to the random genes. For our main enrichment test we set *α*=2.37 × 10^−7^ as in the original analysis but to ensure robustness we re-performed the enrichment test for a wide range of *α*-levels, ranging from 10^−20^ to 0.05, and observed that results were consistent for all *α*-levels considered.

To identify whether the mediating CpG sites were located in functionally relevant regions, we took two main approaches. First, we intersected the CpG positions with the hg19 CpG island annotation track from UCSC Genome Browser ( http://genome.ucsc.edu), to determine whether each site was located in a CpG island, CpG shore (+/−1.5 kb from island) or CpG shelf (+/−1.5 kb from shore). Second, we intersected the CpG positions with ENCODE's ChromHMM annotation for lymphoblastoid cell line GM12878, which uses a hidden Markov model to assign genomic features based on the combinatorial pattern of various chromatin marks^69^. The ChromHMM annotation allowed us to identify CpGs located in promoters, enhancers and insulators. We then used Fisher's exact test to assess whether there was significant enrichment of each feature in mediating CpG sites compared to other CpG sites within the 1,497 genes.

### Query of candidate age-expression associated genes and pathways

A total of 204 candidate genes were identified from a variety of sources including Mendelian ageing disorders, longevity genetics candidates^11^,^12^,^70^,^71^,^72^ and members of key ageing pathways, mainly FOXO/mTOR, key DNA repair genes, regulators of telomere maintenance, and mitochondrial ribosomal proteins^12^,^25^,^71^,^73^. Additional candidates included those from past human or multispecies expression studies^74^,^75^,^76^, and markers of naive or differentiated immune cells^77^. Animal model gene names were translated to human homologue names. All genes and their known human alias names were searched against the discovery and replication results. Thirty-three genes were not tested due to lack of measurement or blood expression below filtered levels. Most candidate genes (*n*=126) were analysed but did not meet the strict discovery thresholds to be carried forward to the replication phase (Supplementary Table 9). Of 45 genes carried into replication, 33 convincingly replicated in whole blood (Supplementary Table 8).

### Transcriptomic age prediction as surrogate biomarker

To investigate how accurate biological age can be predicted from gene expression levels, we performed a leave-one-out prediction analysis, that is, re-running the meta-analysis excluding each of the validation cohorts. For all models, we used the standardized residuals of the gene expression levels, which were obtained by adjusting the gene expression levels for the technical covariates (RNA quality, batch effects) and some biological covariates (sex, fasting status, smoking status and cell counts).

To predict age, we needed to have the estimated effect sizes of the gene expression levels on chronological age (model 1: chronological age ∼gene expression). However, effect sizes from the meta-analysis were for chronological age on gene expression levels (model 2: gene expression ∼chronological age). We used an equivalent transformation to convert the effect size in model 2 to that in model 1 by the following equation:


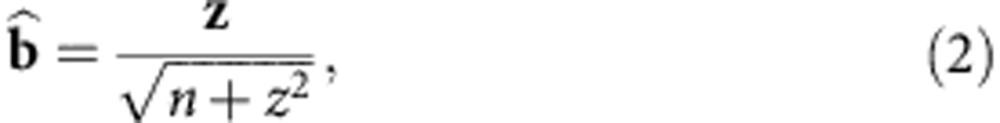


where 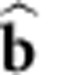 is the effect size of the gene expression level on chronological age (model 1), based on standardized chronological age and standardized gene expression levels, so that it needs to be interpreted in s.d. unit for both chronological age and gene expression level; *z* is the *z*-statistic for association from the meta-analysis; and *n* is the sample size. We then conducted an approximate ridge regression analysis based on a random effect model, which is analogue to the best linear unbiased prediction approach in mixed linear model analysis, to estimate the effect sizes of all 11,908 genes jointly taking correlations between probes into account. The random effect model can be written as:


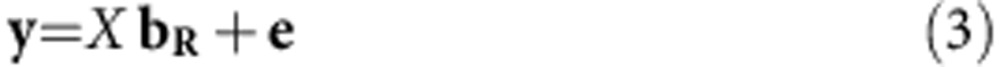


where **y** is the vector of age phenotype and **X** is the matrix of gene expression level, **b**_**R**_ is a vector of effects of gene expression on age with:


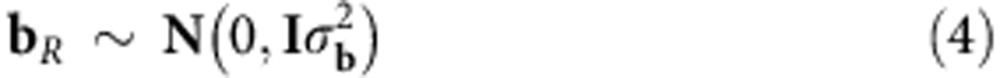


and **e** is a vector of residual with:


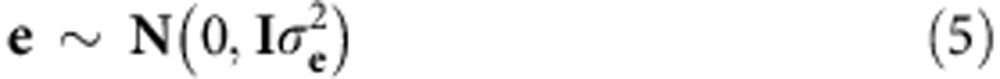


In a ridge regression analysis, **b**_**R**_ can be estimated as


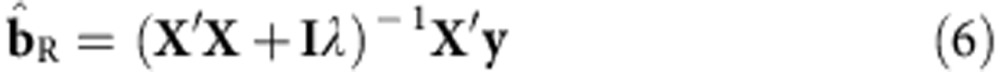


where


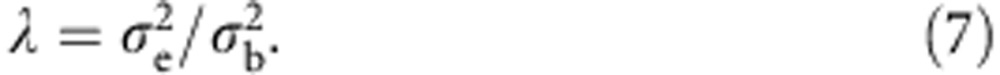


In a single probe based meta-analysis, the analysis is equivalent to:


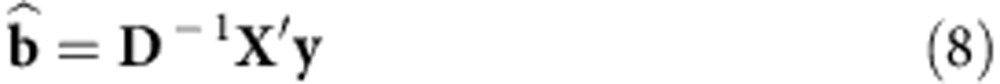


where **b** is a vector of effect sizes estimated from the meta-analysis and **D** is the diagonal matrix of 
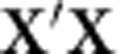
. If the gene expression level of each probe is standardized, the *i*th diagonal element of **D** is:


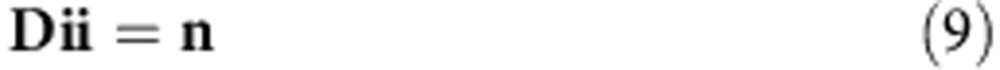


with *n* being the sample size. We therefore have


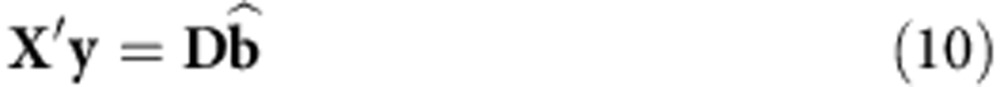


so that


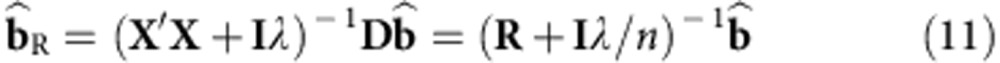


where **R** is the correlation matrix between probes. This method largely follows the method that was proposed to estimate the joint effect sizes of SNPs using summary data from GWAS and linkage disequilibrium between SNPs from a reference sample^78^. We estimated **b**_**R**_ using 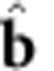 from the meta-analysis (excluding the validation cohort) and probe correlation matrix **R** from reference samples (also independent from the validation cohort).

We calibrated the parameter *λ* using BSGS as the validation cohort (finding a *λ* value that maximized prediction accuracy in BSGS; Supplementary Fig. 10) and applied it to the prediction analysis in the other validation cohorts (Supplementary Data 5A). We call this an approximate method because the correlation matrix **R** consisted of weighted averages (weighted by sample size) from up to six of the discovery cohorts rather than all the samples pooled together. We applied the estimates of the individual genes from the ridge regression analysis to the left-out sample (validation sample) to predict age, and calculated the correlation coefficient of chronological age and the predicted transcriptomic age (Fig. 3a–h).

Since the effect sizes of the probes were estimated from the meta-analyses excluding the validation sample, the validation set is completely independent from the discovery (training) set, so that the prediction accuracy is unbiased. We created the predictor of an individual in the validation cohort as


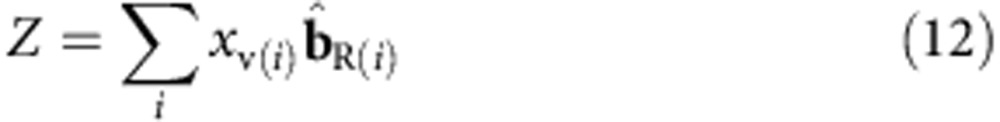


with *x*_v(*i*)_ being the gene expression level of *i*th probe in the validation cohort. We scaled *Z* using the mean and s.d. of chronological age from the validation cohort:


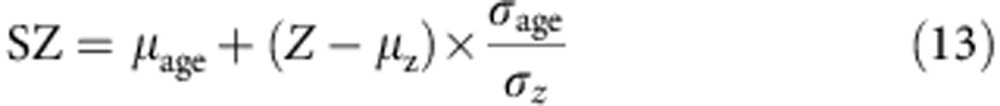


where *μ*_*age*_ and *σ*_*age*_ are the mean and s.d. of chronological age from the validation cohort, and *μ*_*z*_ and *σ*_*z*_ are the mean and s.d. of the predictor *Z*. Delta age was defined as the difference between the scaled transcriptomic predicted age (SZ) and chronological age for each individual.

We explored whether delta age was associated with any multi-systemic biological parameter (or biomarker) of ageing, such as sex, blood pressure, cholesterol levels, glucose levels, and so on. For all biomarkers used, outliers were excluded from the analysis. Associations were tested using a linear model, including the phenotype of interest as the outcome (the dependent variable) and the delta age as the independent variable; all associations were adjusted for chronological age. To overcome the effects of obesity on cardiovascular disease and other traits, we additionally adjusted for BMI in a second model. In additional, we tested whether the biological parameters were directly associated with chronological age (Supplementary Table 20), so:


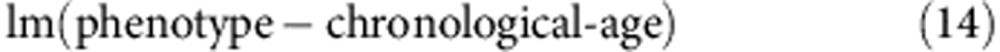


### Transcriptomic age prediction for external cohorts

A general transcriptomic predictor (*Z*) was generated which can be used by external researchers for future purposes. This predictor was calculated using the prediction meta-analysis of all cohorts (except BSGS on which we calibrated the *λ* parameter; Supplementary Fig. 10). Cohorts that have chronological age available should scale the predictor as we did for the validation cohorts (equation (13)), using the mean and s.d. of chronological age and the mean and s.d. of the predictor (*Z*).

To make our predictor useful to cohorts that do not have chronological age available, we further transformed the predictor to a scaled transcriptomic predictor (in years). This scaled predictor was calculated using the mean and s.d. of chronological age from all discovery cohorts in the meta-analysis (equation (13)). Since the individual level age data were not available, the s.d. of chronological age was calculated using the pooled variance method (Supplementary Table 21).

The Transcriptomic Age Prediction (TRAP) webpage contains information on how to calculate transcriptomic age based on data measured with the Illumina HumanHT-12 (v3/v4) Gene Expression BeadChips or the Affymetrix Human Exon (1.0 ST) Arrays. After uploading your gene expression data, the function will return a text file whose rows report the estimated transcriptomic age of each subject. The online Transcriptomic Age Predictor can be accessed at: https://trap.erasmusmc.nl/.

## Additional information

**How to cite this article:** Peters, M. J. *et al.* The transcriptional landscape of age in human peripheral blood. *Nat. Commun.* 6:8570 doi: 10.1038/ncomms9570 (2015).

## Supplementary Material

Supplementary Information Supplementary Figures 1-10, Supplementary Tables 1-21, Supplementary Notes 1-2, Supplementary Acknowledgements and Supplementary References

Supplementary Data 1 Discovery, replication and generalization results. Y = yes, N = no; - is negative direction, + = positive direction; weight = sample size, P = p-value; WB = whole blood.

Supplementary Data 2 Number of genes that associate with age across all tissues. 2A: A gene-based overview for age-association across all tissues. 2B: A gene-based overview for significant expression across all tissues.

Supplementary Data 3 Up/down regulated gene set analyses in WEBGESTALT and GENENETWORK.

Supplementary Data 4 Methylation results: Sobel test for methylation-based mediation of age-expression association.

Supplementary Data 5 Transcriptomic age prediction formulas. 5A: Prediction formula weights for all participating cohorts (based on Leave-One-Out Meta-Analyses). 5B: The average "transcriptomic predictor formula" - the GENERAL predictor - for external cohorts

## Figures and Tables

**Table 1:** Top 50 age-associated genes.

		**Discovery**		**Replication**		**Meta-analysis**			**Generalization**	
**Gene**	**Rank**	***Z*****-score**	***P*****-value**	***Z*-score**	***P*****-value**	**Number of samples**	***Z*-score**	***P*****-value**	**Cerebellum**	**Frontal Cortex**
CD248	1	−32.48	2.32E−231	−40.13	4.07E−352	15,266	−51.46	1.62E−577	NA	NA
LRRN3	2	−29.12	2.03E−186	−33.55	7.81E−247	15,266	−44.38	3.53E−430	N	Y (−)
NELL2	3	−23.65	1.18E−123	−23.48	6.93E−122	15,266	−33.31	2.67E−243	N	Y (−)
LEF1	4	−22.18	5.57E−109	−22.46	9.38E−112	15,266	−31.56	1.22E−218	NA	NA
CCR7	5	−21.14	3.59E−99	−22.44	1.48E−111	15,266	−30.83	1.04E−208	NA	NA
ABLIM1	6	−22.32	2.34E−110	−20.73	1.71E−95	15,266	−30.41	4.41E−203	N	Y (+)
GZMH	7	18.68	7.03E−78	20.97	1.26E−97	15,266	28.07	2.39E−173	NA	NA
MYC	8	−18.96	3.36E−80	−19.51	9.94E−85	15,266	−27.20	5.96E−163	NA	NA
CD27	9	−17.65	1.07E−69	−20.68	5.13E−95	15,266	−27.15	2.76E−162	NA	NA
FAM102A	10	−19.46	2.24E−84	−18.68	7.11E−78	15,266	−26.95	5.68E−160	N	Y (+)*
SERPINE2	11	−16.08	3.71E−58	−20.95	1.91E−97	14,385	−26.34	7.66E−153	Y (−)	Y (−)**
SLC16A10	12	−20.39	2.29E−92	−16.51	3.15E−61	13,809	−26.15	1.00E−150	Y (+)	Y (−)
FCGBP	13	−15.76	5.50E−56	−20.83	2.49E−96	15,266	−25.95	1.65E−148	NA	Y (+)*
GPR56	14	17.52	9.47E−69	19.02	1.21E−80	15,266	25.86	2.03E−147	NA	NA
BACH2	15	−17.82	4.64E−71	−17.75	1.85E−70	15,266	−25.14	1.71E−139	N	NA
SYT11	16	17.23	1.72E−66	18.23	3.24E−74	15,266	25.08	8.82E−139	Y (−)	Y (−)
PDE9A	17	−17.21	2.22E−66	−18.20	5.44E−74	15,266	−25.05	1.91E−138	N	N
NG	18	−17.01	7.41E−65	−17.52	9.87E−69	15,266	−24.42	1.16E−131	NA	NA
FLNB	19	−15.78	4.26E−56	−18.61	2.87E−77	15,266	−24.36	4.94E−131	N	Y (+)**
NT5E	20	−17.45	3.29E−68	−16.59	8.23E−62	15,039	−24.06	6.98E−128	NA	NA
FGFBP2	21	17.45	3.51E−68	15.79	3.51E−56	15,266	23.47	8.43E−122	NA	NA
TGFBR3	22	15.00	7.73E−51	17.66	9.15E−70	15,266	23.13	2.41E−118	N	Y (+)*
ITM2C	23	−14.41	4.24E−47	−17.73	2.45E−70	15,266	−22.78	7.22E−115	N	N
ATF7IP2	24	−15.52	2.73E−54	−16.61	5.85E−62	15,266	−22.73	2.34E−114	NA	Y (−)*
CR2	25	−16.29	1.10E−59	−15.85	1.51E−56	15,266	−22.71	3.49E−114	NA	NA
FAIM3	26	−17.92	8.65E−72	−14.22	7.40E−46	15,266	−22.65	1.41E−113	NA	NA
PHGDH	27	−13.25	4.56E−40	−18.30	8.10E−75	15,266	−22.39	4.85E−111	N	Y (+)*
LDHB	28	−15.63	4.33E−55	−15.96	2.42E−57	15,266	−22.34	1.55E−110	Y (−)*	Y (−)**
SIRPG	29	−15.64	4.16E−55	−15.45	7.71E−54	15,266	−21.97	5.58E−107	NA	NA
FCRL6	30	13.29	2.83E−40	17.65	9.90E−70	15,266	21.95	9.70E−107	NA	NA
PDE7A	31	−15.58	9.40E−55	−15.37	2.68E−53	15,266	−21.88	4.42E−106	NA	NA
NSIP	32	−14.44	3.12E−47	−16.19	5.74E−59	15,266	−21.68	3.13E−104	N	N
PAICS	33	−16.00	1.26E−57	−14.34	1.29E−46	15,266	−21.42	9.39E−102	N	Y (+)**
BZW2	34	−14.93	2.19E−50	−15.18	4.55E−52	15,266	−21.29	1.42E−100	Y (−)**	Y (−)**
OXNAD1	35	−15.59	9.09E−55	−14.32	1.71E−46	15,266	−21.12	5.66E−99	NA	NA
CX3CR1	36	14.09	4.14E−45	15.66	3.04E−55	14,385	21.07	1.67E−98	NA	NA
SCML1	37	−14.00	1.58E−44	−15.69	1.92E−55	15,266	−21.01	5.02E−98	NA	NA
RPL22	38	−14.91	3.03E−50	−14.79	1.79E−49	15,266	−20.99	8.61E−98	N	Y (−)**
LDLRAP1	39	−14.57	4.19E−48	−14.82	1.15E−49	15,266	−20.78	6.69E−96	N	NA
RHOC	40	12.89	4.89E−38	15.93	3.71E−57	15,266	20.43	8.94E−93	N	Y (+)
LTB	41	−14.90	3.55E−50	−14.02	1.11E−44	15,266	−20.43	9.52E−93	NA	NA
FAM134B	42	−15.17	5.88E−52	−13.43	3.96E−41	15,266	−20.19	1.31E−90	N	N
LBH	43	−14.18	1.29E−45	−14.22	7.04E−46	15,266	−20.07	1.28E−89	NA	Y (−)**
PRSS23	44	14.07	5.76E−45	14.07	6.25E−45	15,266	19.89	5.11E−88	NA	NA
SUSD3	45	−14.26	4.01E−46	−13.91	5.30E−44	14,385	−19.87	6.90E−88	NA	NA
PIK3IP1	46	−14.93	2.02E−50	−13.13	2.16E−39	15,266	−19.81	2.58E−87	Y (+)*	Y (+)**
MFGE8	47	−12.46	1.23E−35	−15.34	4.09E−53	15,266	−19.70	2.06E−86	N	N
AGMAT	48	−13.77	4.14E−43	−14.09	4.34E−45	15,266	−19.70	2.31E−86	NA	NA
NKG7	49	14.43	3.17E−47	13.42	4.53E−41	15,266	19.67	3.67E−86	NA	NA
PPP2R2B	50	13.49	1.81E−41	14.26	4.19E−46	15,266	19.63	9.40E−86	Y (−)*	Y (−)

NA, not expressed.

For the 50 most significant age-associated genes, the discovery *P*-value (and Z-score), the replication *P*-value (and Z-score), and the meta-analysis *P*-value (and sample size and *Z*-score) are shown. The last two columns display whether the genes were also significantly associated with age in the brain tissues cerebellum and frontal cortex.

*Y=P*<0.05; Y*=P<0.01; Y**=P<0.0001; *N=P≥*0.05; (−) or (+) gives the direction of the effect with age.

**Table 2:** Meta-analysis of associations between transcriptomic Δage with twelve biological ageing phenotypes.

	**Adjusted for chronological age**				**Adjusted for chronological age+BMI**			
**Phenotype of Interest**	***Z*-score**	***P*****-value**	**Direction**	***N***	***Z*-score**	P*-value	**Direction**	***N***
Sex: 0=male, 1=female	−2.7610	5.76E−03	−−+−−+−+	8,836	0.7500	4.53E−01	−−+++−−+	8,829
Systolic bloodpressure: mm Hg	9.8510	6.78E−23	++++++++	8,571	9.3740	6.97E−21	++++++++	8,564
Diastolic bloodpressure: mm Hg	7.7200	1.16E−14	++++++++	8,568	6.8020	1.03E−11	++++++++	8,561
Total cholesterol levels: mmol l^−1^	5.4190	5.99E−08	+++++−++	8,688	4.6370	3.53E−06	+++++−++	8,681
HDL cholesterol levels: mmol l^−1^	4.4630	8.07E−06	+++++−+−	8,687	5.8310	5.52E−09	+++++−++	8,680
Fasting glucose levels: mmol l^−1^	6.9330	4.11E−12	++++++??	7,330	5.8920	3.82E−09	++++++??	7,323
BMI: kg m^−2^	5.3860	7.21E−08	++++++++	8,829	NA	NA	NA	NA
Waist hip ratio	3.3800	7.25E−04	++??++++	4,837	1.9370	5.27E−02	++??++++	4,837
Hand grip strength: kg	−1.5120	1.31E−01	++?−????	3,651	−1.1760	2.40E−01	++?−????	3,651
Renal function	0.8740	3.82E−01	+++−+?−?	7,317	−0.4890	6.25E−01	+++−+?−?	7,310
Mini mental state exam score	−1.3130	1.89E−01	−−??????	1,492	−1.3810	1.67E−01	−−??????	1,492
Current smoking: 0=no, 1=yes	5.5100	3.59E−08	+−?+++−−	7,379	3.2040	1.36E−03	−−?+++−−	7,379

BMI, body mass index; NA, not available.

We tested whether the transcriptomic delta age was associated with twelve biological phenotypes known to be associated with chronological age. Gene expression levels were adjusted for plate ID, RNA quality score, fasting state, sex, smoking status and cell counts. Association results of all cohorts were meta-analysed. After adjustment for chronological age and BMI (right columns), systolic blood pressure, diastolic blood pressure, total cholesterol levels, HDL cholesterol levels, and fasting glucose levels were significantly positively associated with the delta age (*P*<4.17E−3). Samples predicted to be older (positive delta age) consistently had higher levels for these ageing phenotypes.

Delte age=transcriptomic age−chronological age; +*Z*-score=increasing phenotype with higher predicted age; *−Z*-score=decreasing phenotype with higher predicted age; *if *P*<(0.05/12=4.17E−3), significance has been reached.
