# Supplementary material for: The transcriptional landscape of age in human peripheral blood
Source: Nat Commun. 2015 Oct 22;6:8570. doi: 10.1038/ncomms9570 (PMC4639797; doi:10.1038/ncomms9570)
Supplement: Supplementary Information — Supplementary Figures 1-10, Supplementary Tables 1-21, Supplementary Notes 1-2, Supplementary Acknowledgements and Supplementary References [file ncomms9570-s1.pdf]

<u>**SUPPLEMENTARY FIGURES**</u>

Supplementary Figure 1. Age-associated genes in discovery and replication samples.

A.

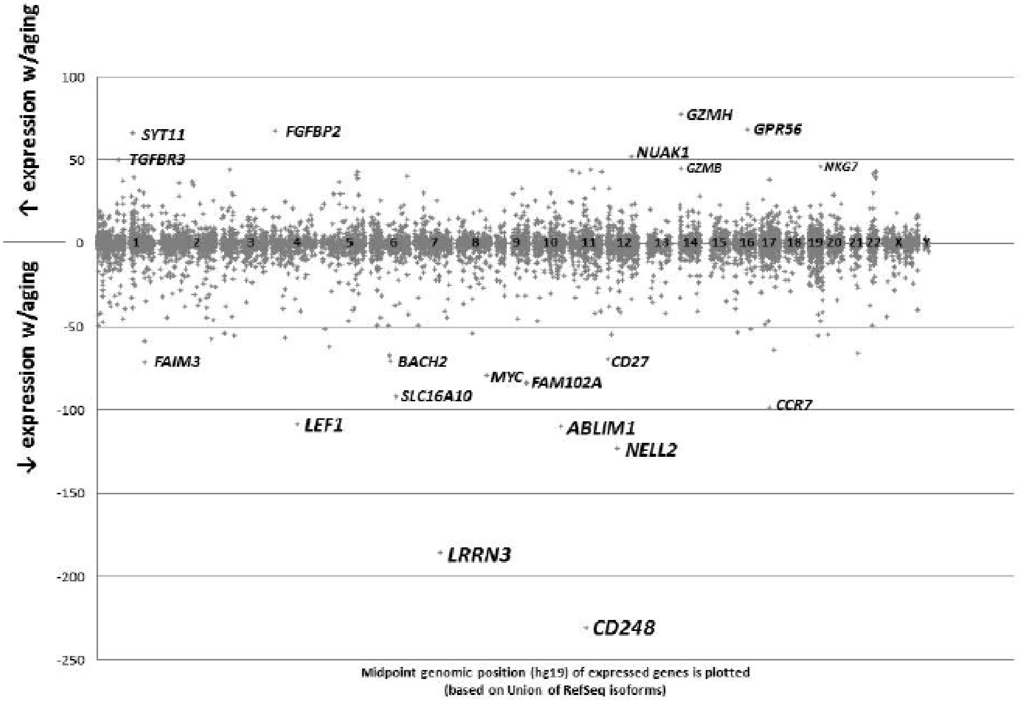

B.

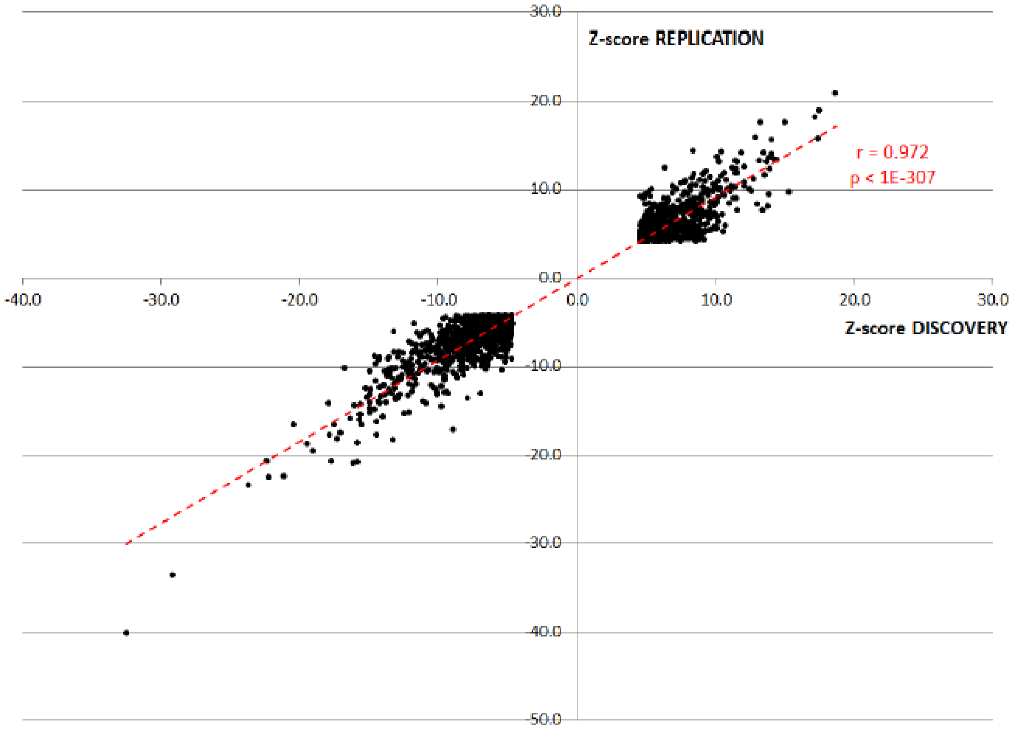

(1A) Manhattan mirror plot of the whole blood expression-age discovery meta-analysis. Each dot represents a gene; the x-axis gives the position of the gene on the genome (chr 1 – chrY), and the y-axis represents the direction of the effect (-/+) and the p-value of the association (-logP). (**1B**) The scatterplot illustrating the correlation between the discovery z-scores and the replication z-scores of the 1,497 age-associated genes.

Supplementary Figure 2. Venn diagram for overlapping genes between whole blood and brain tissue.

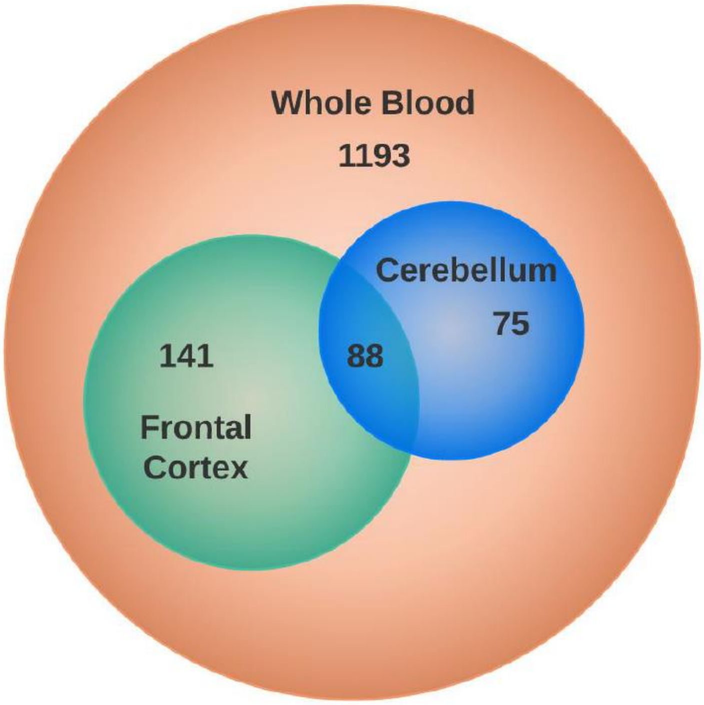

Venn diagram for the age-associated genes identified in the analyses for whole blood, cerebellum, and frontal cortex. 88 genes were differentially expressed with age across all three tissues.

Supplementary Figure 3. Match quality of random genes selected for methylation-expression enrichment analysis.

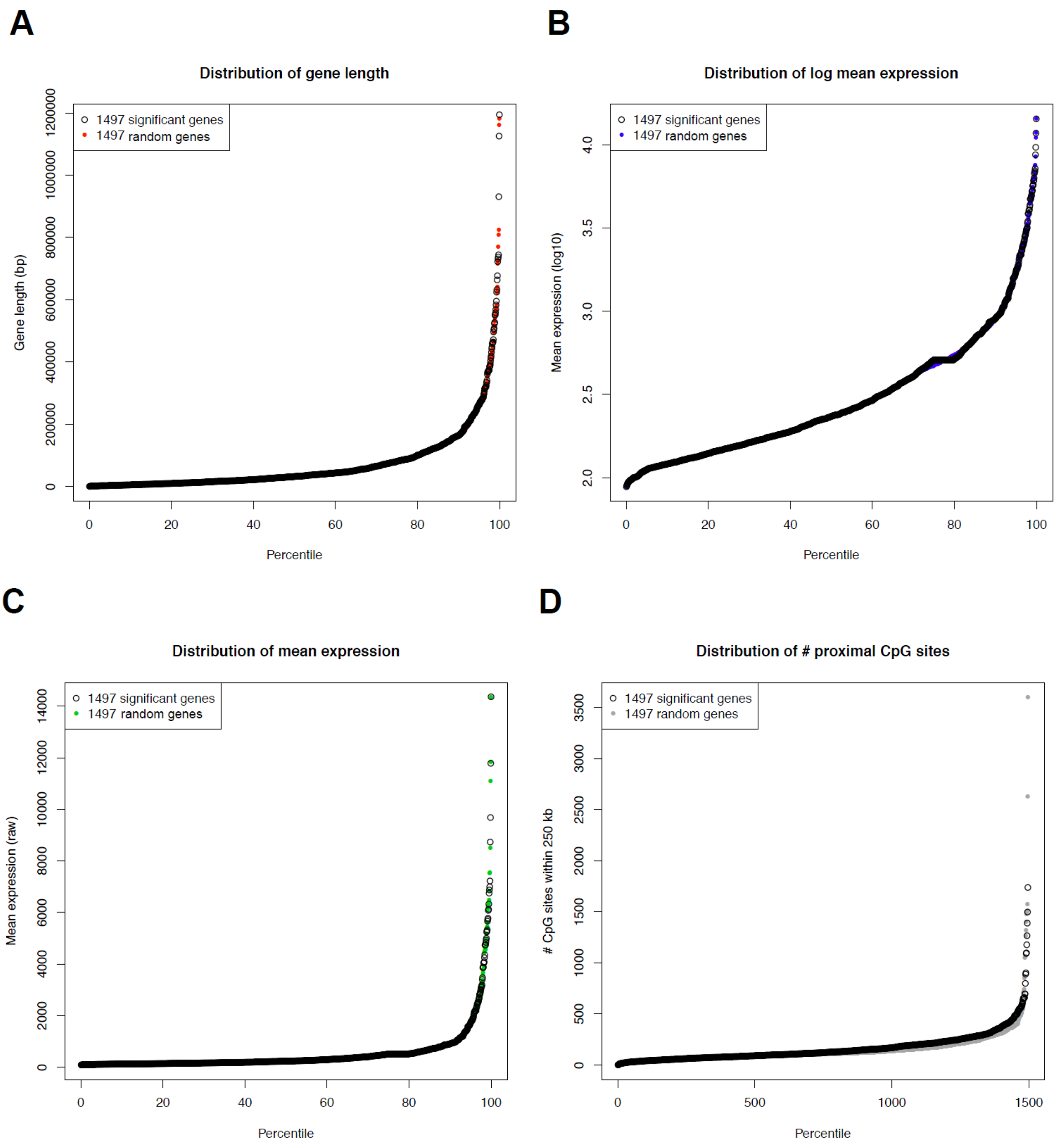

(3A) Distribution of gene length of 1,497 age-associated genes and 1,497 random genes (matched on gene length and the log of mean expression as described in Methods). **(3B)** Distribution of log mean expression of 1,497 age-associated genes and 1,497 random genes. **(3C)** Distribution of mean expression of 1,497 age-associated genes and 1,497 random genes. **(3D)** Distribution of number of proximal CpG sites (within 250kb) of 1,497 age-associated genes and 1,497 random genes.

Supplementary Figure 4. Quantile-Quantile (QQ) plots of the 1,497 age-associated genes versus 1,497 random genes, separately by cohort.

A.

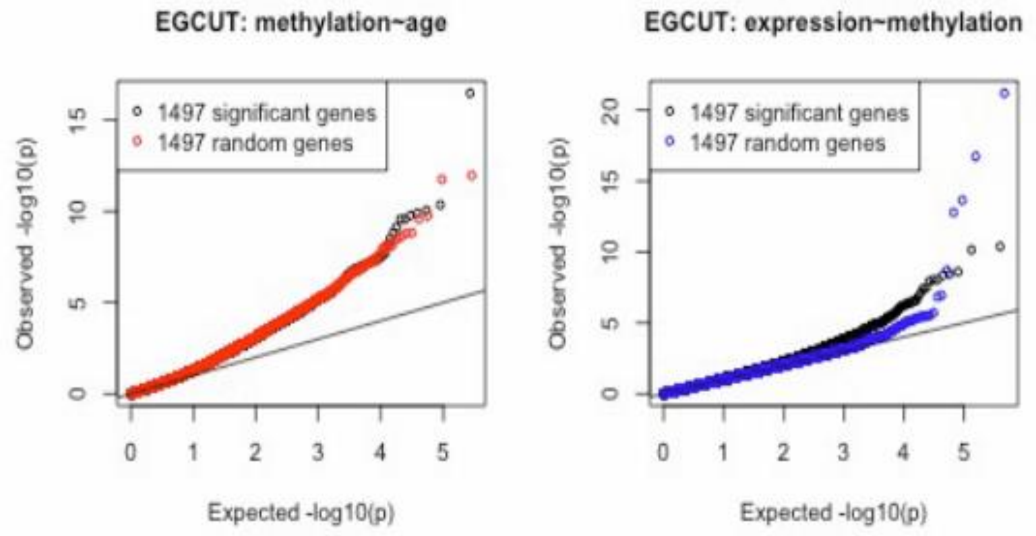

B.

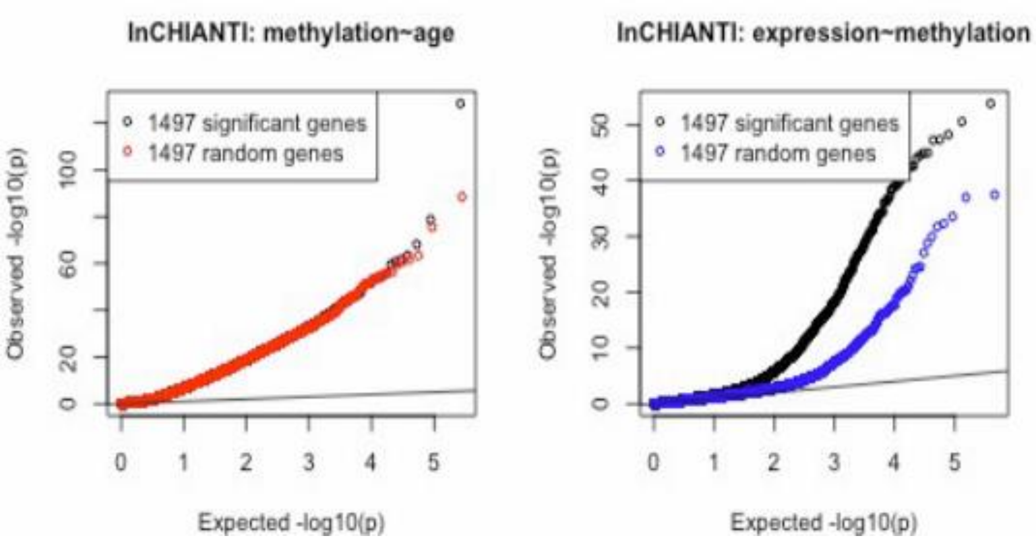

C.

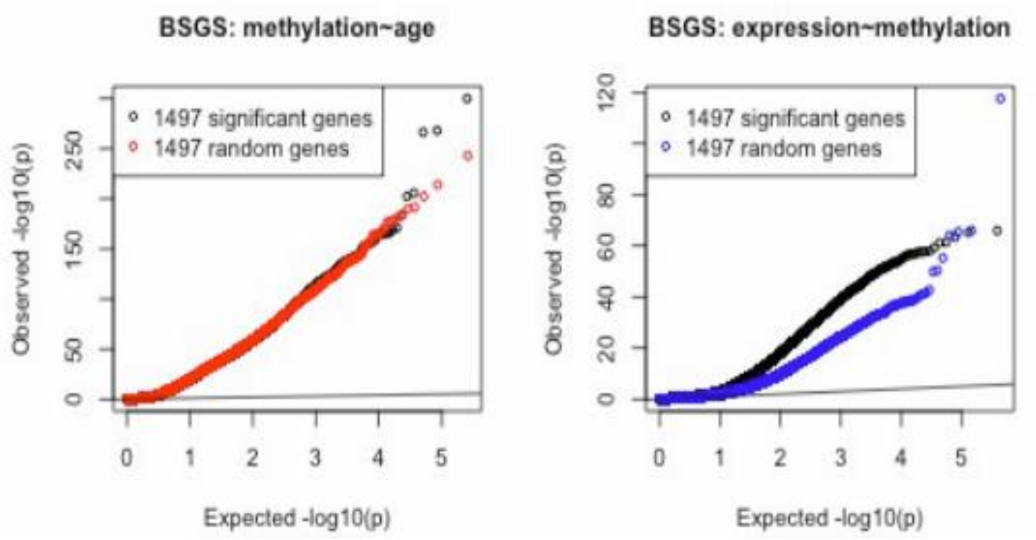

D.

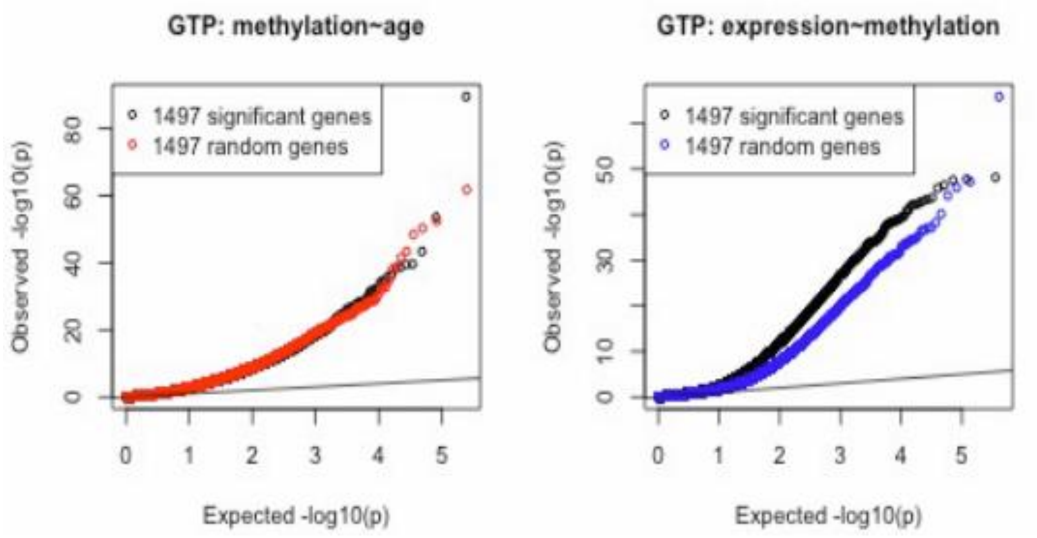
GTP: methylation~age
1497 significant genes
1497 random genes
Observed -log10(p)
Expected -log10(p)
GTP: expression~methylation
1497 significant genes
1497 random genes
Observed -log10(p)
Expected -log10(p)

E.

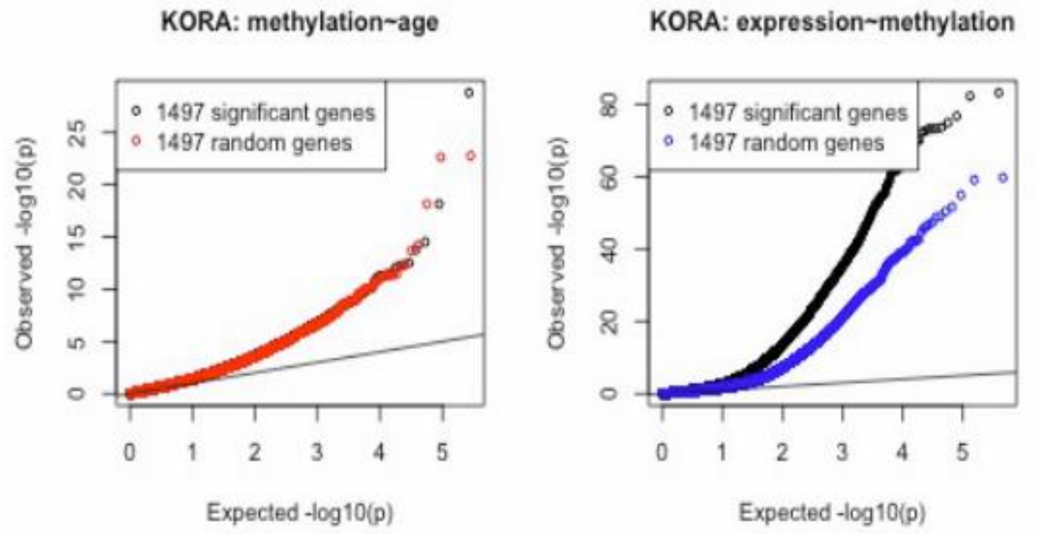
KORA: methylation~age
1497 significant genes
1497 random genes
Observed -log10(p)
Expected -log10(p)
KORA: expression~methylation
1497 significant genes
1497 random genes
Observed -log10(p)
Expected -log10(p)

F.

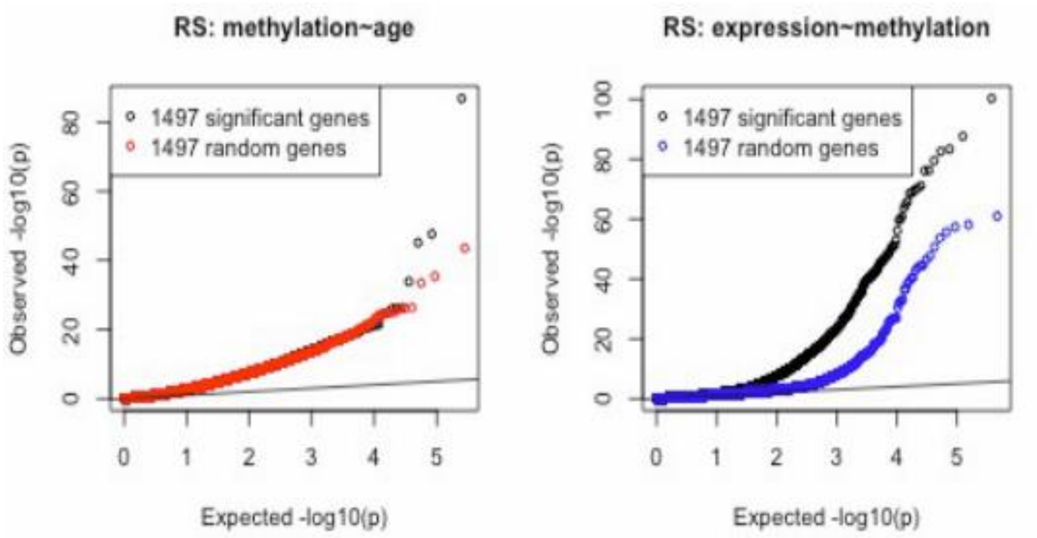
RS: methylation~age
1497 significant genes
1497 random genes
Observed -log10(p)
Expected -log10(p)
RS: expression~methylation
1497 significant genes
1497 random genes
Observed -log10(p)
Expected -log10(p)

G.

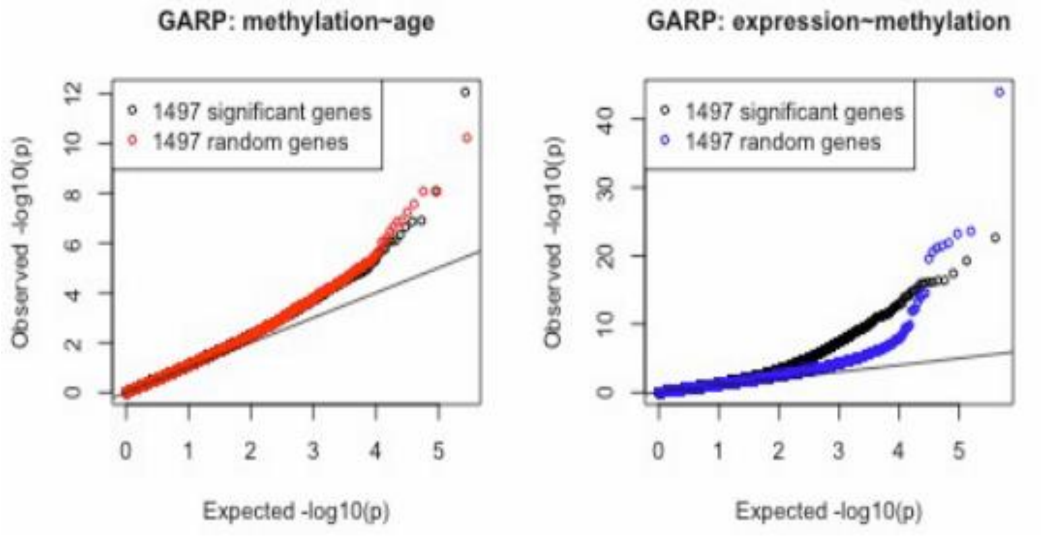

Q-Q plots of the observed P values (-log10P) for the methylation~age associations (*left panels)* and the methylation~expression associations (*right panels*). The plots in black show P values from the 1,497 significant age-associated genes, whereas the plots in red and blue show P values for 1,497 random genes. Plots A-G represent the results for the separate cohorts with methylation data: **(4A)** EGCUT, **(4B)** INCHIANTI, **(4C)** BSGS, **(4D)** GTP, **(4E)** KORA, **(4F)** RS, and **(4G)** GARP. These plots were the basis for Figure 2, which shows the results of the meta-analysis across all cohorts.

Supplementary Figure 5. Quantile-Quantile (QQ) plots of the 1,497 age-associated genes versus 1,497 random genes, separately by genomic region.

A.

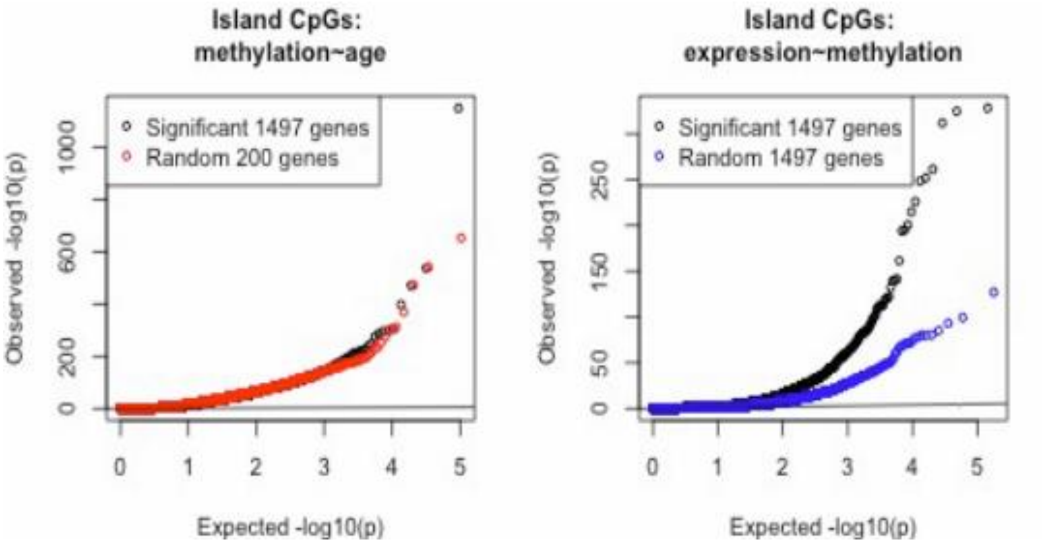

B.

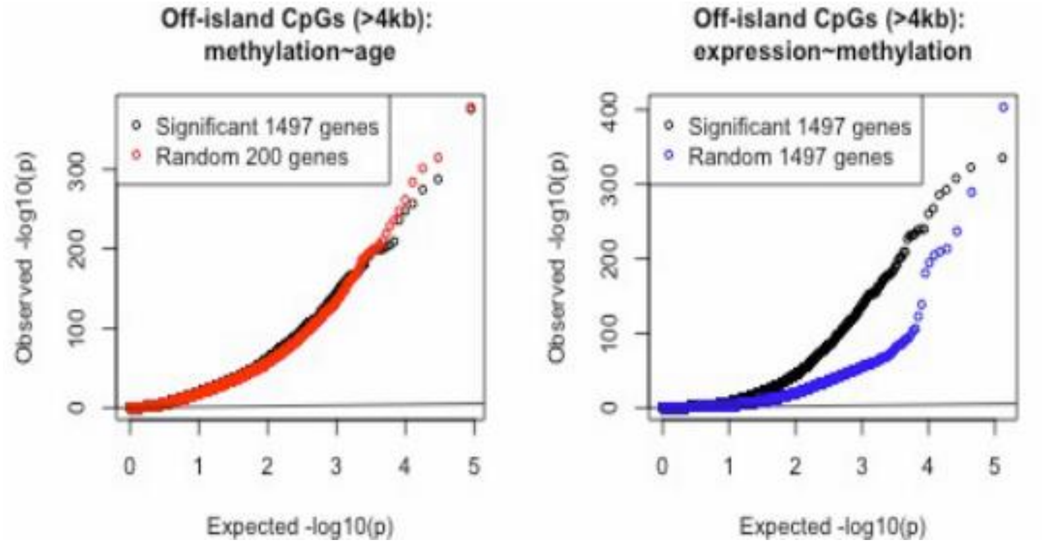

C.

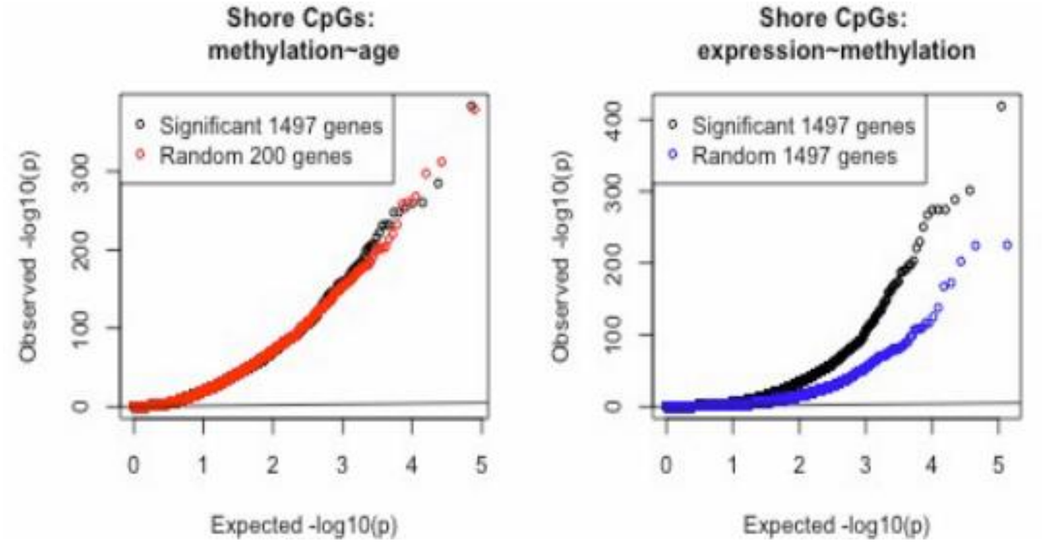

D.

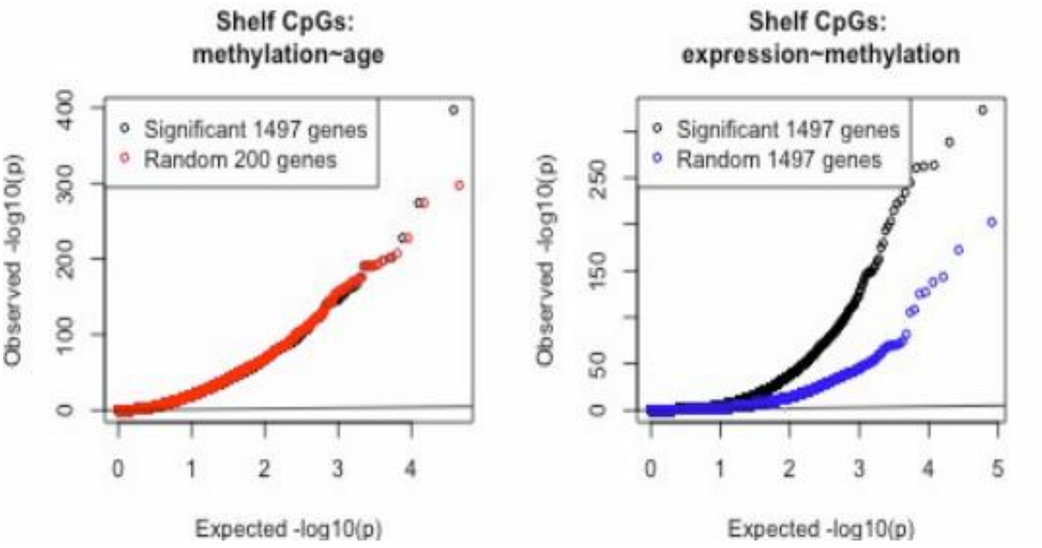
Shelf CpGs:
methylation~age
Significant 1497 genes
Random 200 genes
Observed -log10(p)
Expected -log10(p)
Shelf CpGs:
expression~methylation
Significant 1497 genes
Random 1497 genes
Observed -log10(p)
Expected -log10(p)

E.

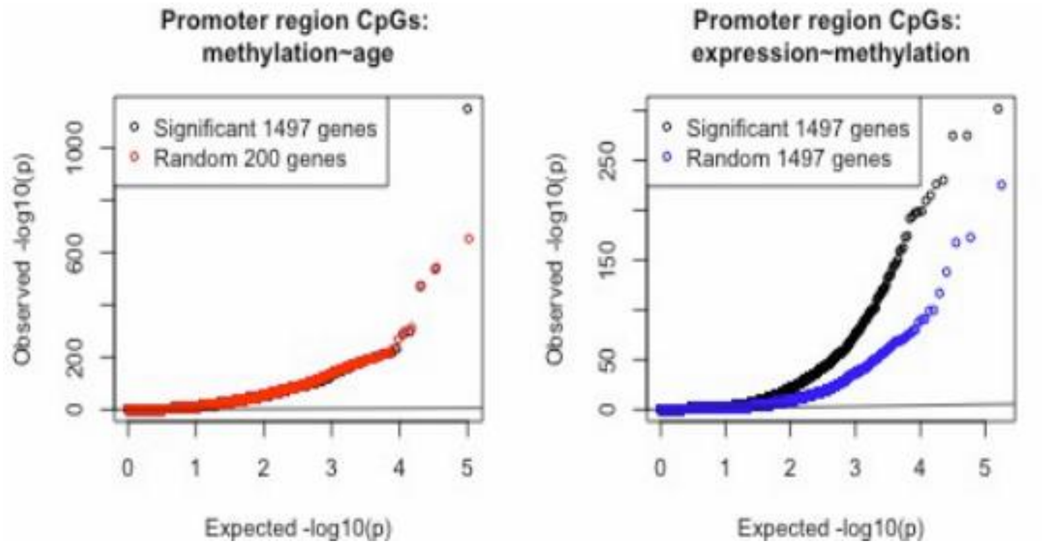
Promoter region CpGs:
methylation~age
Significant 1497 genes
Random 200 genes
Observed -log10(p)
Expected -log10(p)
Promoter region CpGs:
expression~methylation
Significant 1497 genes
Random 1497 genes
Observed -log10(p)
Expected -log10(p)

F.

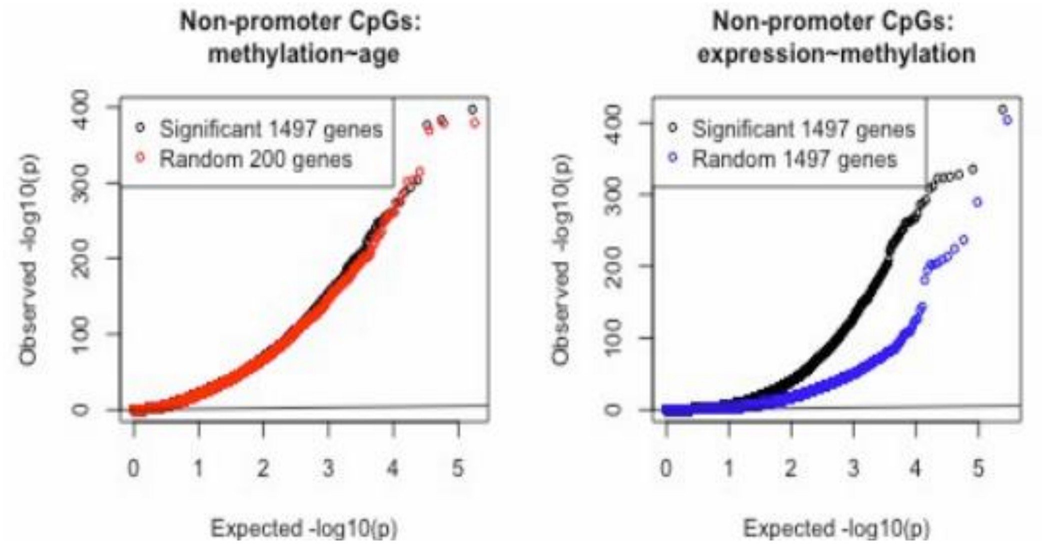
Non-promoter CpGs:
methylation~age
Significant 1497 genes
Random 200 genes
Observed -log10(p)
Expected -log10(p)
Non-promoter CpGs:
expression~methylation
Significant 1497 genes
Random 1497 genes
Observed -log10(p)
Expected -log10(p)

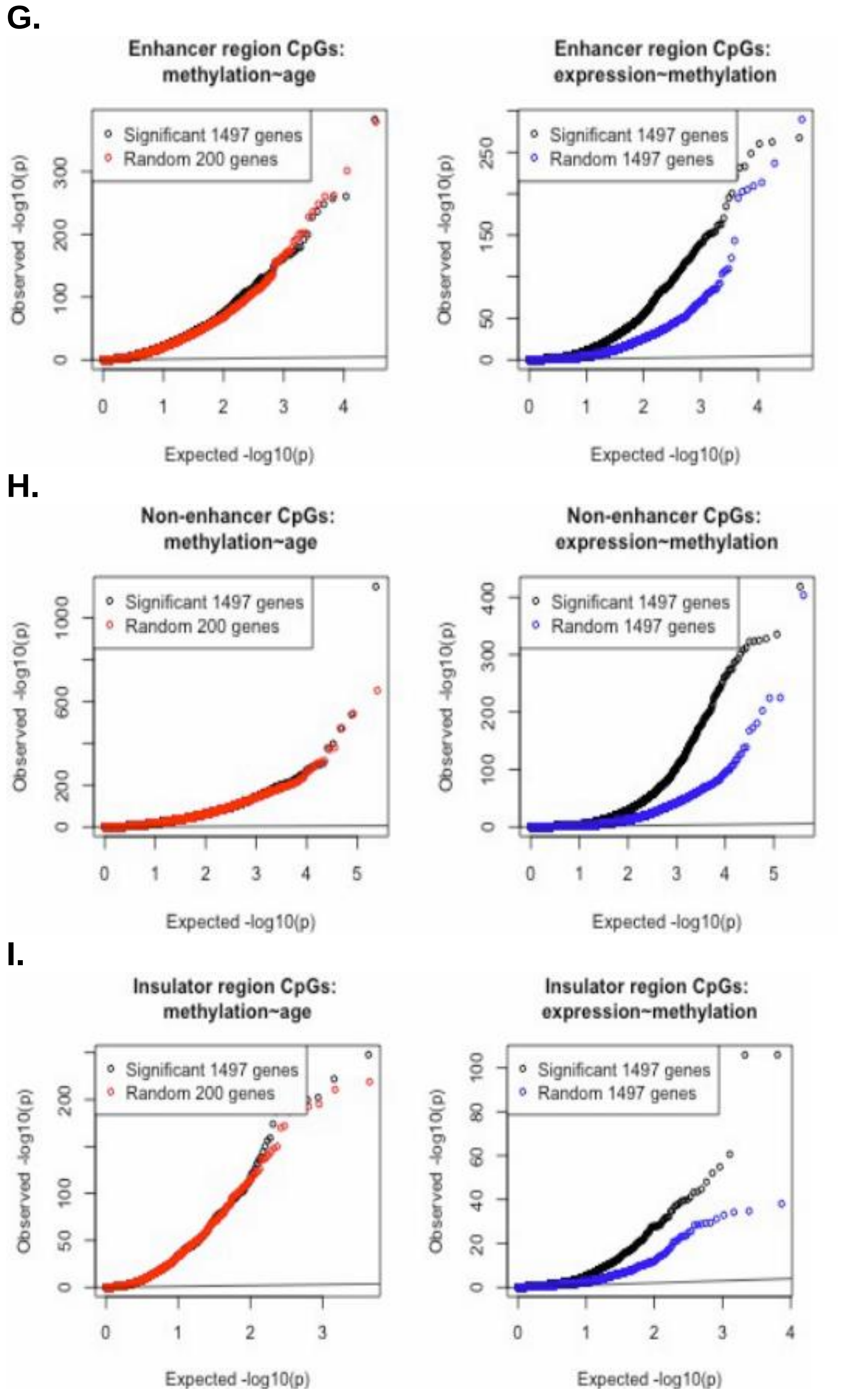
G.
Enhancer region CpGs: methylation~age
Significant 1497 genes
Random 200 genes
Observed -log10(p)
Expected -log10(p)
Enhancer region CpGs: expression~methylation
Significant 1497 genes
Random 1497 genes
Observed -log10(p)
Expected -log10(p)
H.
Non-enhancer CpGs: methylation~age
Significant 1497 genes
Random 200 genes
Observed -log10(p)
Expected -log10(p)
Non-enhancer CpGs: expression~methylation
Significant 1497 genes
Random 1497 genes
Observed -log10(p)
Expected -log10(p)
I.
Insulator region CpGs: methylation~age
Significant 1497 genes
Random 200 genes
Observed -log10(p)
Expected -log10(p)
Insulator region CpGs: expression~methylation
Significant 1497 genes
Random 1497 genes
Observed -log10(p)
Expected -log10(p)

J.

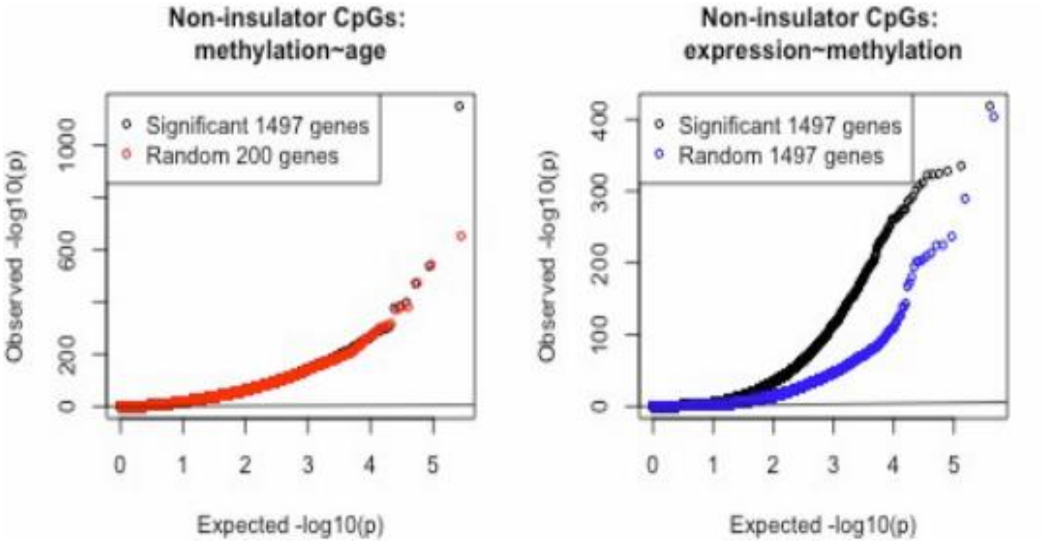

Q-Q plots of the observed P values (-log10P) for the methylation~age associations (*left panels)* and the methylation~expression associations (*right panels*). The plots in black show P values from the 1,497 significant age-associated genes, whereas the plots in red and blue show P values for 1,497 random genes. Plots A-G represent the results for different genomic regions: (**5A)** CpG-islands, (**5B)** Off-island, (**5C)** Shores, (**5D)** Shelves, (**5E**) Promoters, (**5F)** Non-promoters, (**5G)** Enhancers, (**5H)** Non-enhancers, (**5I)** Insulators, and (**5J)** Non-insulators.

Supplementary Figure 6. Odds ratios for enrichment of CpG sites with significant Sobel test p-values for the different genomic regions.

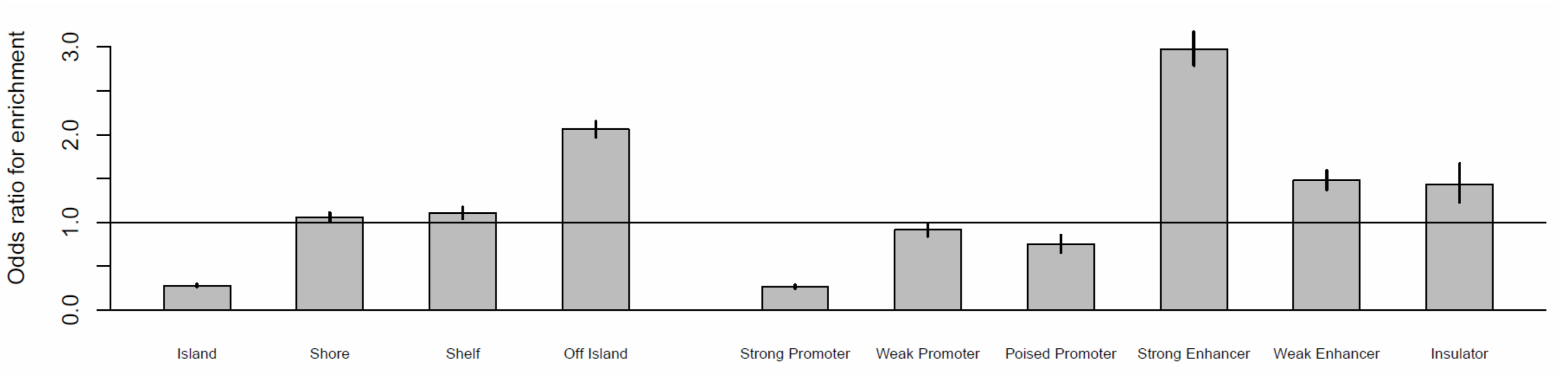

Each odds ratio is based on comparison of CpG sites within 250kb of the 1,497 age-associated genes with significant Sobel test p-values (N=8,234) vs. other CpG sites within 250kb of the 1,497 age-associated genes (N=191,832). Vertical bars represent the 95% confidence interval for each odds ratio. Strong enhancers, weak enhancers, insulators, and off island regions were enriched for CpG sites with significant Sobel test p-values.

Supplementary Figure 7. Comparison of the transcriptomic and epigenetic age prediction methods.

A.

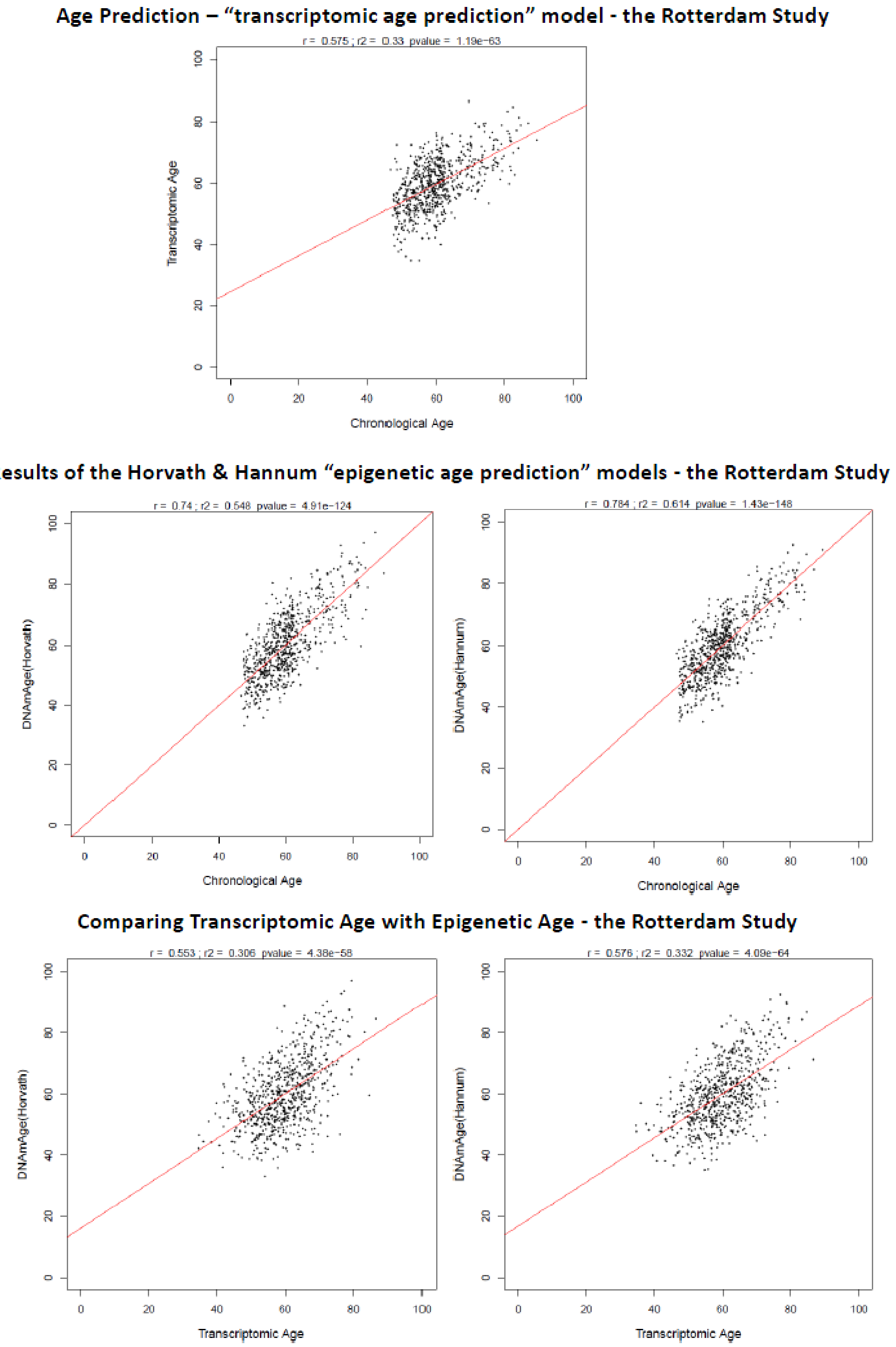

(7A) The top figure represents the correlation between transcriptomic age (y-axis) and chronological age (x-axis). The figures in the middle show the correlation between epigenetic age (y-axis: left = Horvath, right = Hannum) and chronological age (x-axis). The figures at the bottom explain the correlation between the transcriptomic predictor (x-axis) and the epigenetic age (y-axis: left = Horvath, right = Hannum) – *results based on a selection of 671 Rotterdam Study samples having both gene expression & DNA methylation data available.*

B.

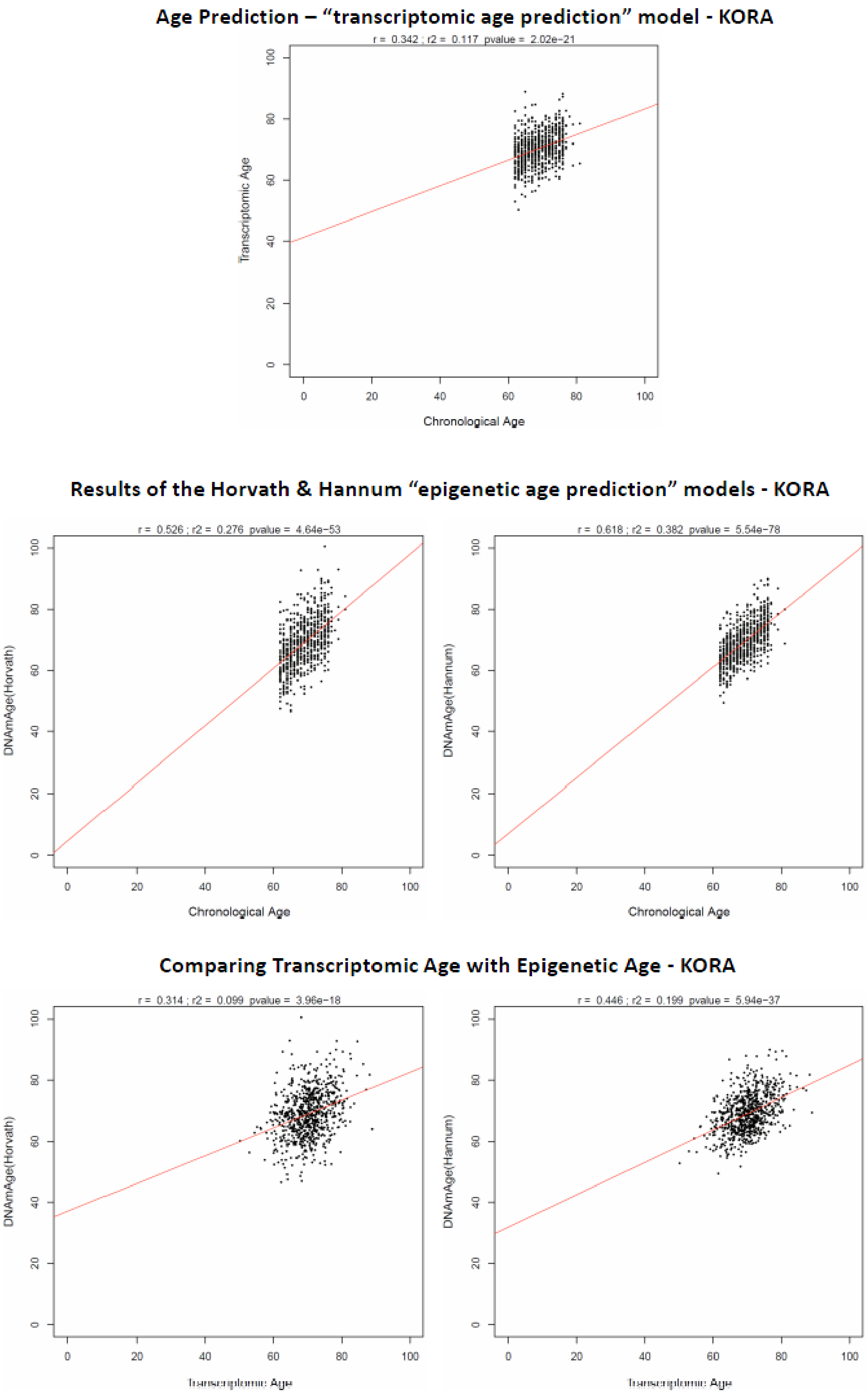

(7B) The top figure represents the correlation between transcriptomic age (y-axis) and chronological age (x-axis). The figures in the middle show the correlation between epigenetic age (y-axis: left = Horvath, right = Hannum) and chronological age (x-axis). The figures at the bottom explain the correlation between the transcriptomic predictor (x-axis) and the epigenetic age (y-axis: left = Horvath, right = Hannum) – *results based on a selection of 725 KORA samples having both gene expression & DNA methylation data available.*

Supplementary Figure 8. Overview of the study design.

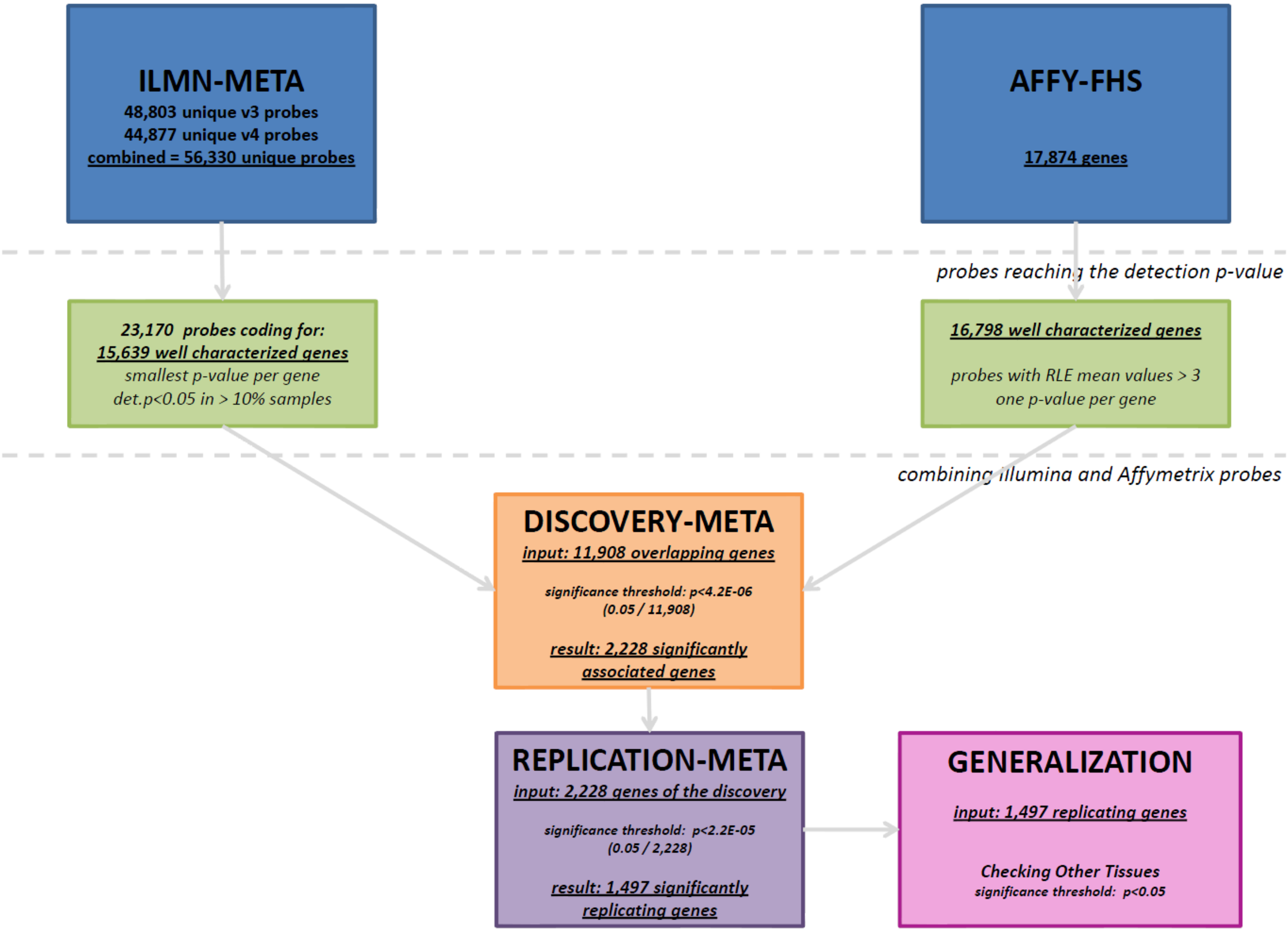

Affymetrix probe IDs and Illumina probe IDs have been combined to be able to run one meta-analysis on 11,908 overlapping genes. 2,228 genes were significantly associated with age in the discovery meta-analysis (p<4.2E-6), and 1,497 genes have been replicated in the replication meta-analysis (p<2.2E-5). The 1,497 replicated age-associated genes have been checked in other tissues and ethnicities (p<0.05).

Supplementary Figure 9. Age distributions of the discovery (A-F), replication (G-N), and generalization cohorts (O-V).

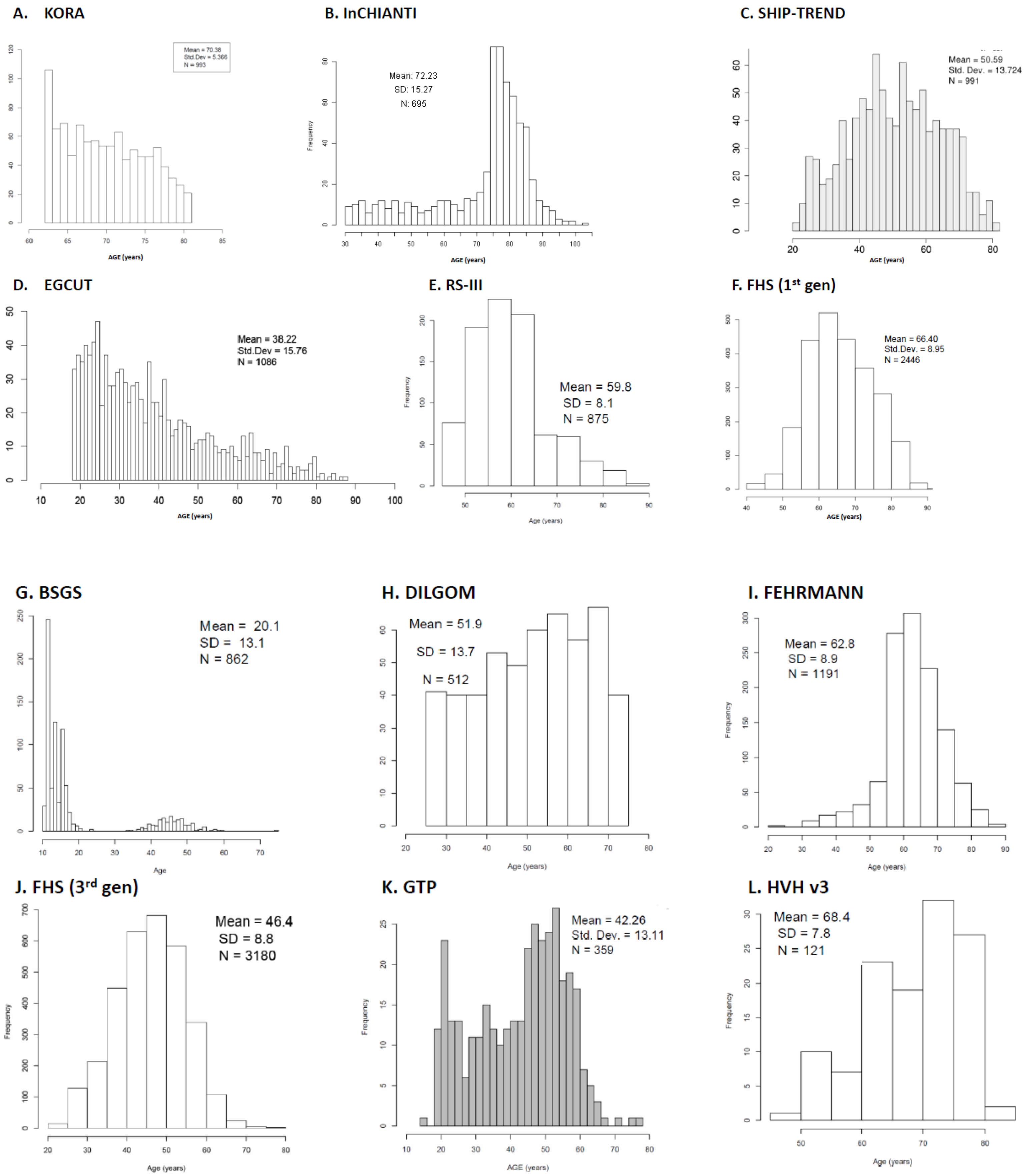

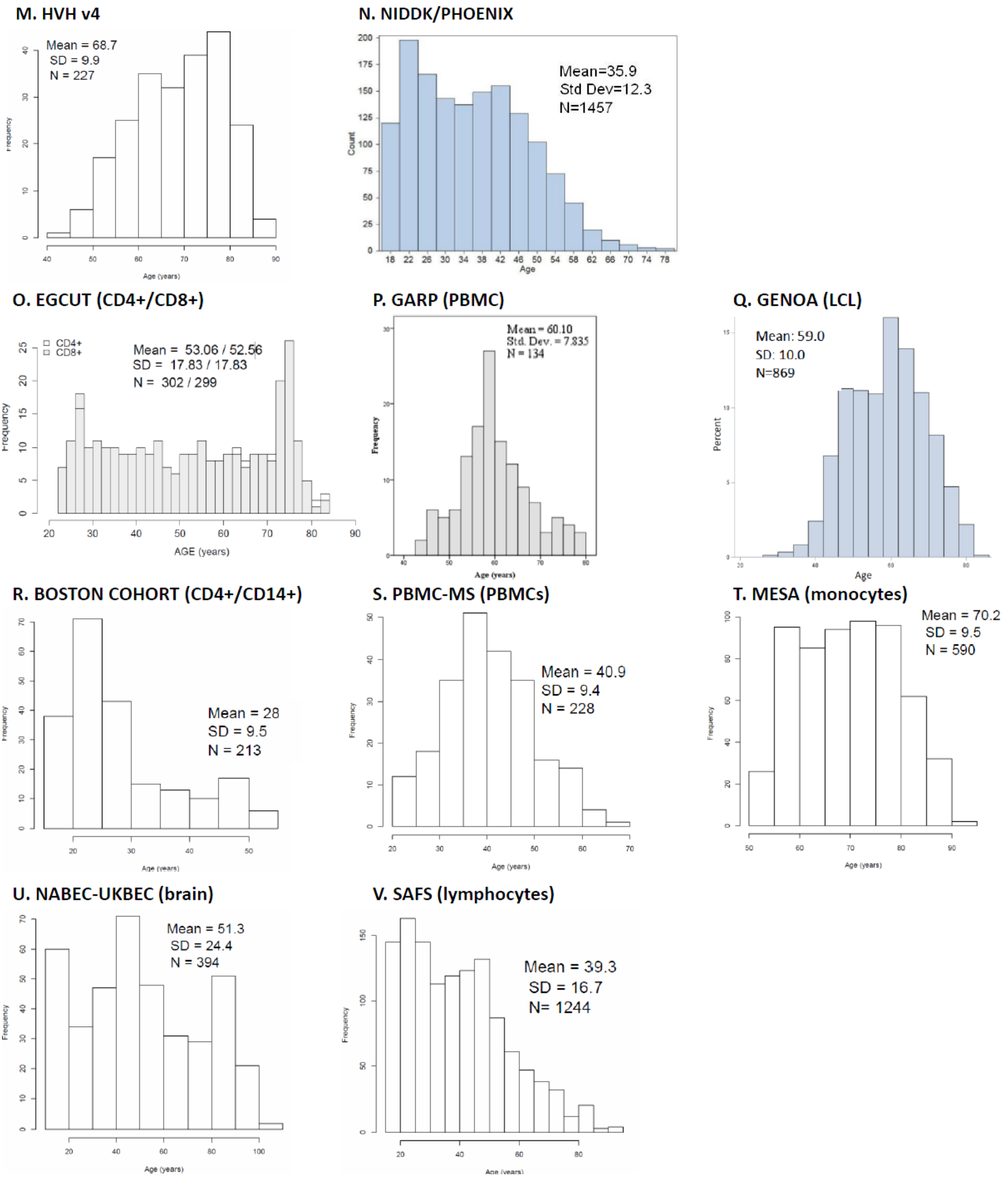

(9A) KORA, **(9B)** INCHIANTI, **(9C)** SHIP-TREND, **(9D)** EGCUT, **(9E)** RS, **(9F)** FHS-OFFSPRING(GEN2: EXAM 8), **(9G)** BSGS, **(9H)** DILGOM, **(9I)** FEHRMANN, **(9J)** FHS-GEN3 (GEN3: EXAM 2), **(9K)** GTP, **(9L)** HVH (v3), **(9M)** HVH (v4), **(9N)** NIDDK/PHOENIX, **(9O)** EGCUT (CD4+ and CD8+ cells), **(9P)** GARP (PBMCs), **(9Q)** GENOA (LCLs), **(9R)** BOSTON COHORT (CD4+ and CD14+ cells), **(9S)** PBMC-MS (PBMCs), **(9T)** MESA (monocytes), **(9U)** NABEC-UKBEC (brain cells), **(9V)** SAFS (lymphocytes).

Supplementary Figure 10. Calibration of the parameter lambda (λ) for the approximate ridge regression analysis in the BSGS cohort.

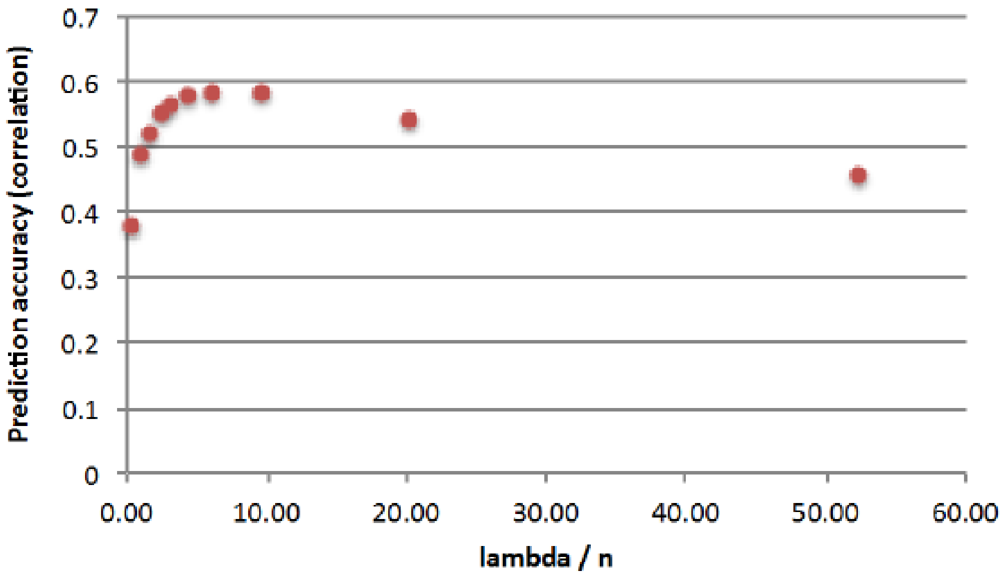

Lambda (λ) is the shrinkage parameter that is used to estimate effect sizes in the ridge regression (see methods). We re-ran the meta-analysis excluding the BSGS cohort. We performed the approximate ridge regression analysis with a range of lambda values, and calculated the correlation between the predictor and the actual age in BSGS (shown on y-axis). Shown on x-axis is *lambda / n* with *n* being the sample size of the meta-analysis (excluding BSGS). The maximum prediction accuracy is achieved at lambda = *6.07 * n* with n being the sample size.

SUPPLEMENTARY TABLES

Supplementary Table 1. The number of age-associated genes across different ancestries.

	Caucasian *(meta-analysis)*	***Native American*** *(NIDDK/Phoenix)*	***Hispanic*** *(SAFHS)*	***African American*** *(GTP)*
# samples	15,266	1,457	1,244	359
# expressed genes	1,497	1,424	595	1,481
**% EXPRESSED**	**100.0%**	**95.1%**	**39.7%**	**98.9%**
# expressed genes - same direction		1,326	562	1,219
**% SAME DIRECTION**		**93.1%**	**94.5%**	**82.3%**
# expressed genes - same direction & p<0.05		1,005	440	392
**% GENERALIZED**		**70.6%**	**73.9%**	**26.5%**

Supplementary Table 2: The number of age-associated genes across different tissue types.

	Whole blood	***Cerebellum***	***Frontal cortex***	***CD4+***	***CD8+***	***CD14+***	***LCLs***	***Lymphocytes***	***PBMCs***
# samples	15,266	394	394	515	299	567	869	1244	362
# expressed genes	1,497	860	882	1,470	1,203	1,473	1,315	595	1,497
**% EXPRESSED**	**100.0%**	**57.4%**	**58.9%**	**98.2%**	**80.4%**	**98.4%**	**87.8%**	**39.7%**	**100.0%**
# expressed genes - same direction		414	438	1168	1100	1020	596	562	1126
**% SAME DIRECTION**		**48.1%**	**49.7%**	**79.5%**	**91.4%**	**69.2%**	**45.3%**	**94.5%**	**75.2%**
# expressed genes - same direction & p<0.05		163	229	440	663	400	220	440	328
**% GENERALIZED**		**19.0%**	**26.0%**	**29.9%**	**55.1%**	**27.2%**	**16.7%**	**73.9%**	**21.9%**

Supplementary Table 3: A matrix of shared age-associated genes across different tissues.

<u>Tissue</u><br>(# age-associated genes)	Whole blood<br>*(1,497 genes)*	Cerebellum<br>*(860 genes)*	Frontal cortex<br>*(882 genes)*	CD4+<br>*(1,470 genes)*	CD8+<br>*(1,203 genes)*	CD14+<br>*(1,473 genes)*	LCLs<br>*(1,315 genes)*	Lymphocytes<br>*(595 genes)*	PBMCs<br>*(1,497 genes)*
**Whole blood**<br>***(1,497 genes)***	1,497	163	229	440	663	400	220	440	328
**Cerebellum**<br>***(860 genes)***	163	163	88	53	79	63	18	43	26
**Frontal cortex**<br>***(882 genes)***	229	88	229	76	107	82	35	69	40
**CD4+**<br>***(1,470 genes)***	440	53	76	440	283	132	63	165	131
**CD8+**<br>***(1,203 genes)***	663	79	107	283	663	180	111	238	169
**CD14+**<br>***(1,473 genes)***	400	63	82	132	180	400	55	137	73
**LCLs**<br>***(1,315 genes)***	220	18	35	63	111	55	220	64	52
**Lymphocytes**<br>***(595 genes)***	440	43	69	165	238	137	64	440	111
**PBMCs**<br>***(1,497 genes)***	328	26	40	131	169	73	52	111	328

Supplementary Table 4. 34 genes encoding ribosomal subunits were significantly associated with age: 33 were negatively associated with age.

UNIQUEID	RANK	GENID	WEIGHT	ZSCORE	DIRECTION	P-VALUE
48072	38	RPL22	15266	-20.987	-	8.61E-98
13742	317	RPS6	15266	-12.692	-	6.57E-37
9389	356	RPS4X	15266	-12.083	-	1.29E-33
40921	374	RPL18	15266	-11.924	-	8.87E-33
60831	387	RPL13	15266	-11.827	-	2.85E-32
45616	414	RPS23	14385	-11.561	-	6.49E-31
17817	432	RPL8	14385	-11.474	-	1.78E-30
67967	451	RPS13	15266	-11.319	-	1.06E-29
45323	453	RPL5	15266	-11.302	-	1.28E-29
42546	478	RPLP0	15266	-11.116	-	1.04E-28
62881	596	RPSAP58	15266	-10.241	-	1.30E-24
19552	610	RPS2	15266	-10.167	-	2.77E-24
32696	615	RPL13P5	15266	-10.136	-	3.81E-24
55317	704	RPL19	15266	-9.742	-	2.00E-22
46193	711	RPP40	15266	-9.709	-	2.77E-22
34217	730	RPS16	14385	-9.618	-	6.72E-22
48839	791	RPL4	15266	-9.392	-	5.87E-21
17484	887	RPL36	15266	-9.014	-	1.99E-19
66008	910	RPS20	15266	-8.921	-	4.60E-19
52719	973	RPS14	15266	-8.643	-	5.49E-18
4260	1071	RPL13AP3	15266	-8.28	-	1.23E-16
45816	1098	RPL28	15266	-8.187	-	2.67E-16
57181	1159	RPS29	15266	-8.001	-	1.24E-15
45497	1167	RPL17	15266	-7.955	-	1.79E-15
14935	1255	RPSA	12620	-7.666	-	1.77E-14
18988	1257	RPS6KB1	15266	-7.647	-	2.06E-14
68466	1275	RPP21	15266	-7.558	-	4.08E-14
48699	1303	RPL11	15266	-7.47	-	8.03E-14
11427	1338	RPL27A	15266	-7.34	-	2.14E-13
58212	1404	RPL30	15266	-7.113	-	1.14E-12
65095	1413	RPL35	15266	-7.053	-	1.75E-12
63762	1458	RPS6KA1	15266	6.798	+	1.06E-11
62334	1460	RPL23AP64	15266	-6.773	-	1.26E-11
49929	1472	RPS18	15266	-6.628	-	3.41E-11

Supplementary Table 5. 10 genes encoding mitochondrial ribosomal genes were significantly associated with age: all were negatively associated with age.

UNIQUEID	RANK	GENID	WEIGHT	ZSCORE	DIRECTION	P-VALUE
54318	480	MRPL45	15266	-11.083	-	1.52E-28
34287	641	MRPS27	15266	-9.994	-	1.62E-23
44427	794	MRPL3	14385	-9.38	-	6.61E-21
36113	820	MRPL24	15266	-9.285	-	1.62E-20
13521	840	MRPS26	15266	-9.196	-	3.71E-20
6683	866	MRPS31	15266	-9.057	-	1.34E-19
35886	894	MRPS18B	15266	-8.984	-	2.61E-19
14726	1025	MRPS33	15266	-8.447	-	2.99E-17
22151	1128	MRPS9	15266	-8.098	-	5.58E-16
36300	1492	MRPL35	15266	-6.419	-	1.37E-10

Supplementary Table 6. The number of whole blood functional cluster genes significantly associated with age across the different tissues.

<u>Functional cluster</u><br>*(# genes expressed)*	**Whole blood**<br>*(278 genes)*	**Cerebellum**<br>*(131 genes)*	**Frontal cortex**<br>*(136 genes)*	**CD4+**<br>*(275 genes)*	**CD8+**<br>*(228 genes)*	**CD14+**<br>*(277 genes)*	**LCLs**<br>*(252 genes)*	**Lymphocytes**<br>*(129 genes)*	**PBMCs**<br>*(278 genes)*
+1	77	4	6	40	42	21	10	32	10
+2	9	2	6	4	4	5	5	4	1
+3	8	0	1	1	2	2	2	2	0
+4	6	2	3	2	4	2	0	1	0
-1.1	50	5	9	6	19	28	1	23	19
-1.2	37	7	5	8	18	23	1	14	11
-1.3	22	3	2	6	14	3	1	6	7
-2	57	1	1	11	23	10	17	17	17
-3	12	0	0	1	8	9	4	1	0
**AVG # of SIGN functional clusters genes**	**278**	**24**	**33**	**79**	**134**	**103**	**41**	**100**	**65**

Supplementary Table 7. The percentage of whole blood functional cluster genes significantly associated with age across the different tissues.

<u>Functional cluster</u><br>*(# genes expressed)*	**Whole blood**<br>*(278 genes)*	**Cerebellum**<br>*(131 genes)*	**Frontal cortex**<br>*(136 genes)*	**CD4+**<br>*(275 genes)*	**CD8+**<br>*(228 genes)*	**CD14+**<br>*(277 genes)*	**LCLs**<br>*(252 genes)*	**Lymphocytes**<br>*(129 genes)*	**PBMCs**<br>*(278 genes)*
+1	100.0%	5.2%	7.8%	51.9%	54.5%	27.3%	13.0%	41.6%	13.0%
+2	100.0%	22.2%	66.7%	44.4%	44.4%	55.6%	55.6%	44.4%	11.1%
+3	100.0%	0.0%	12.5%	12.5%	25.0%	25.0%	25.0%	25.0%	0.0%
+4	100.0%	33.3%	50.0%	33.3%	66.7%	33.3%	0.0%	16.7%	0.0%
-1.1	100.0%	10.0%	18.0%	12.0%	38.0%	56.0%	2.0%	46.0%	38.0%
-1.2	100.0%	18.9%	13.5%	21.6%	48.6%	62.2%	2.7%	37.8%	29.7%
-1.3	100.0%	13.6%	9.1%	27.3%	63.6%	13.6%	4.5%	27.3%	31.8%
-2	100.0%	1.8%	1.8%	19.3%	40.4%	17.5%	29.8%	29.8%	29.8%
-3	100.0%	0.0%	0.0%	8.3%	66.7%	75.0%	33.3%	8.3%	0.0%
**AVG % of SIGN functional clusters genes**	**100.0%**	**11.7%**	**19.9%**	**25.6%**	**49.8%**	**40.6%**	**18.4%**	**30.8%**	**17.1%**

Supplementary Table 8. The candidate genes carried forward to replication.

GENEID	Lookup GENEID	Reason	exp. dir	Citation(s)	RANK	Dir	disc-p	repl-p
LRRN3	LRRN3	Markers of naïve/differentiated cells	-	Chou et al. 2013	2	-	2.03E-186	7.81E-247
CCR7	CCR7	Markers of naïve/differentiated cells	-	Chou et al. 2013	5	-	3.59E-99	1.48E-111
CD27	CD27	Markers of naïve/differentiated cells	-	Chou et al. 2013	9	-	1.07E-69	5.13E-95
IGJ	IGJ	Multi-tissue, multi-species candidate	+	de Magalhaes/Church 2009	68	-	6.52E-19	1.08E-65
LDH2	LDHB	Downregulated in mice and humans w/ageing in multi-tissue analyses	-	Zahn et al. AGEMAP	28	-	4.33E-55	2.42E-57
VEGFB	VEGFB	MTOR/FOXO pathways	-	Harries et al. 2012	218	-	8.85E-18	6.51E-29
ATM	ATM	Key DNA repair gene	-	Park et al. 2013	86	-	1.25E-42	1.41E-28
FOXO1	FOXO1	MTOR/FOXO pathways	-	Harries et al. 2012	108	-	3.06E-36	3.43E-28
IL7R	IL7R	Age and longevity expression association	-	Passtoors et al. 2012	60	-	7.07E-63	2.85E-24
CAMK4	CAMK4	Longevity genetics candidate	-	Newman & Murabito 2013	96	-	1.98E-47	2.15E-22
SREBF1	SREBF1	MTOR/FOXO pathways	-	Harries et al. 2012	573	-	2.80E-07	2.45E-21
CD28	CD28	Markers of naïve/differentiated cells	-	Chou et al. 2013	127	-	1.69E-45	6.84E-19
LMNA	LMNA	Hutchinson-Gilford Progeria syndrome	+	Luo et al. 2013	382	+	8.88E-16	2.40E-18
ICSBP1	IRF8	Upregulated in mice and humans w/ageing in multi-tissue analyses	+	Zahn et al. AGEMAP	319	-	2.15E-20	4.87E-18
CD70	CD70	Markers of naïve/differentiated cells	-	Chou et al. 2013	297	+	3.63E-23	3.37E-17
MRPS26	MRPS26	Mitochondrial ageing results	-	Houtkooper et al. 2013	840	-	8.64E-07	1.12E-15
MRPL45	MRPL45	Mitochondrial ageing results	-	Houtkooper et al. 2013	480	-	7.13E-15	2.94E-15
FCGR1	FCGR1A	Upregulated in mice and humans w/ageing in multi-tissue analyses	+	Zahn et al. AGEMAP	383	+	2.47E-19	6.19E-15
EIF4G3	EIF4G3	MTOR/FOXO pathways	-	Harries et al. 2012	485	+	6.59E-16	3.60E-14
MRPS18B	MRPS18B	Mitochondrial ageing results	-	Houtkooper et al. 2013	894	-	9.41E-08	2.43E-13
WRN	WRN	Werner syndrome	-	Yu et al. 1996	544	-	2.32E-16	4.66E-12
MRPS27	MRPS27	Mitochondrial ageing results	-	Houtkooper et al. 2013	641	-	2.38E-13	8.81E-12
MRPL24	MRPL24	Mitochondrial ageing results	-	Houtkooper et al. 2013	820	-	6.65E-11	3.98E-11
MRPS9	MRPS9	Mitochondrial ageing results	-	Houtkooper et al. 2013	1128	-	1.26E-07	7.75E-10
MRPL3	MRPL3	Mitochondrial ageing results	-	Houtkooper et al. 2013	794	-	1.41E-13	2.34E-09
MRPS31	MRPS31	Mitochondrial ageing results	-	Houtkooper et al. 2013	866	-	1.57E-12	7.63E-09
STAT3	STAT3	MTOR/FOXO pathways	-	Harries et al. 2012	1219	+	2.95E-08	4.28E-08
MRPS33	MRPS33	Mitochondrial ageing results	-	Houtkooper et al. 2013	1025	-	1.93E-12	7.00E-07
ANXA5	ANXA5	Multi-tissue, multi-species candidate	+	de Magalhaes/Church	987	+	5.88E-14	2.38E-06
NFKB1	NFKB1	MTOR/FOXO pathways	-	Harries et al. 2012	940	-	4.66E-16	4.82E-06
RPS6KB1	RPS6KB1	MTOR/FOXO pathways	-	Harries et al. 2012	1257	-	2.60E-10	5.62E-06
S6K	RPS6KB1	MTOR/FOXO pathways	-	Harries et al. 2012	1257	-	2.60E-10	5.62E-06
MRPL35	MRPL35	Mitochondrial ageing results	-	Houtkooper et al. 2013	1492	-	1.44E-06	1.91E-05
FOXO3A	*FOXO3*	*MTOR/FOXO pathways*	-	*Harries et al. 2012*	*na*	+	*4.41E-09*	*9.99E-05*
MRPS6	*MRPS6*	*Mitochondrial ageing results*	-	*Houtkooper et al. 2013*	*na*	-	*1.12E-06*	*1.33E-04*
LYZ	*LYZ*	*Multi-tissue, multi-species candidate*	+	*de Magalhaes/Church 2009*	*na*	+	*1.88E-08*	*5.54E-04*
CTSZ	*CTSZ*	*Multi-tissue, multi-species candidate*	+	*de Magalhaes/Church 2009*	*na*	+	*1.36E-06*	*2.18E-03*
MRPL46	*MRPL46*	*Mitochondrial ageing results*	-	*Houtkooper et al. 2013*	*na*	-	*2.70E-06*	*2.26E-03*
ADARB1	*ADARB1*	*Longevity genetics candidate*	*?*	*Newman & Murabito 2013*	*na*	-	*4.67E-08*	*2.83E-02*
GRN	*GRN*	*Multi-tissue, multi-species candidate*	+	*de Magalhaes/Church 2009*	*na*	+	*3.48E-11*	*3.10E-02*
MRPL36	*MRPL36*	*Mitochondrial ageing results*	-	*Houtkooper et al. 2013*	*na*	-	*1.72E-06*	*4.06E-02*
CETP	*CETP*	*Longevity genetics candidate*	+	*Newman & Murabito 2013*	*na*	+	*1.84E-11*	*1.06E-01*
SLC25A17	*SLC25A17*	*Downregulated in mice and humans w/ageing in multi-tissue analyses*	-	*Zahn et al. AGEMAP*	*na*	-	*9.69E-07*	*3.42E-01*
CDKN2B	*CDKN2B*	*Longevity candidate gene*	+	*Baker et al. 2008*	*na*	+	*7.89E-07*	*7.45E-01*

Supplementary Table 9: The candidate genes not carried forward to replication.

GENEID	Lookup GENEID	Reason	Citation(s)	RANK
ADARB2	ADARB2	Longevity genetics candidate	Newman & Murabito 2013	3984
ADIPOQ	ADIPOQ	Longevity genetics or biology candidate	Barzilai & Gabriely 2010	9016
AKT1	AKT1	Longevity genetics candidate	Newman & Murabito 2013	5162
ANXA3	ANXA3	Multi-tissue, multi-species candidate	de Magalhaes/Church 2009	4022
ARPP19	ARPP19	Downregulated in mice and humans w/ageing in multi-tissue analyses	Zahn et al. AGEMAP	10836
ASF1A	ASF1A	Age and longevity expression association	Passtoors et al. 2012	2844
ATP5G3	ATP5G3	Multi-tissue, multi-species candidate	de Magalhaes/Church 2009	9622
ATP5J	ATP5J	Downregulated in mice and humans w/ageing in multi-tissue analyses	Zahn et al. AGEMAP	6638
BANF1	BANF1	Nestor-Guillermo Progeria syndrome	Mendelian ageing disorder	4363
BLM	BLM	DNA repair genes	Key DNA repair gene	5492
BRCA1	BRCA1	DNA repair genes	Key DNA repair gene	6096
C1QA	C1QA	Multi-tissue, multi-species candidate	de Magalhaes/Church 2009	11600
C3	C3	Multi-tissue, multi-species candidate	de Magalhaes/Church 2009	2901
CAT	CAT	Longevity genetics or biology candidate	Barzilai & Gabriely 2010	10608
CETP	CETP	Longevity genetics or biology candidate	Barzilai & Gabriely 2010	1591
CPSF5	NUDT21	Downregulated in mice and humans w/ageing in multi-tissue analyses	Zahn et al. AGEMAP	3232
CTSH	CTSH	Multi-tissue, multi-species candidate	de Magalhaes/Church 2009	2380
CTSS	CTSS	Multi-tissue, multi-species candidate	de Magalhaes/Church 2009	2308
CXCR4	CXCR4	Upregulated in mice and humans w/ageing in multi-tissue analyses	Zahn et al. AGEMAP	6812
EIF4E	EIF4E	MTOR/FOXO pathways	Harries et al. 2012	2644
EIF4EBP1	EIF4EBP1	MTOR/FOXO pathways	Harries et al. 2012	4534
ERCC8	ERCC8	Cockayne syndrome	Mendelian ageing disorder	6625
FOXO4	FOXO4	MTOR/FOXO pathways	Harries et al. 2012	5304
GIP	C1QTNF1	Longevity candidate gene	Sebastiani	10428
HIF1A	HIF1A	MTOR/FOXO pathways	Harries et al. 2012	8798
IGF1R	IGF1R	MTOR/FOXO pathways	Kenyon 2011	2770
INSR	INSR	MTOR/FOXO pathways	Harries et al. 2012	6856
LKB1	STK11	MTOR/FOXO pathways	Harries et al. 2012	10505
MDH1	MDH1	Downregulated in mice and humans w/ageing in multi-tissue analyses	Zahn et al. AGEMAP	4275
MGST1	MGST1	Multi-tissue, multi-species candidate	de Magalhaes/Church 2009	2724
MRE11	MRE11A	DNA repair genes	Key DNA repair gene	3888
MRE11A	MRE11A	Downregulated in mice and humans w/ageing in multi-tissue analyses	Zahn et al. AGEMAP	3888
MRP63	MRP63	Mitochondrial ageing results	Houtkooper et al. 2013	8838
MRPL1	MRPL1	Mitochondrial ageing results	Houtkooper et al. 2013	3705
MRPL10	MRPL10	Mitochondrial ageing results	Houtkooper et al. 2013	5773
MRPL11	MRPL11	Mitochondrial ageing results	Houtkooper et al. 2013	10556
MRPL12	MRPL12	Mitochondrial ageing results	Houtkooper et al. 2013	4716
MRPL13	MRPL13	Mitochondrial ageing results	Houtkooper et al. 2013	6335
MRPL14	MRPL14	Mitochondrial ageing results	Houtkooper et al. 2013	10108
MRPL15	MRPL15	Mitochondrial ageing results	Houtkooper et al. 2013	7351
MRPL16	MRPL16	Mitochondrial ageing results	Houtkooper et al. 2013	7019
MRPL17	MRPL17	Mitochondrial ageing results	Houtkooper et al. 2013	9959
MRPL18	MRPL18	Mitochondrial ageing results	Houtkooper et al. 2013	4032
MRPL19	MRPL19	Mitochondrial ageing results	Houtkooper et al. 2013	8375
MRPL2	MRPL2	Mitochondrial ageing results	Houtkooper et al. 2013	4326
MRPL20	MRPL20	Mitochondrial ageing results	Houtkooper et al. 2013	NA

MRPL21	MRPL21	Mitochondrial ageing results	Houtkooper et al. 2013	5971
MRPL22	MRPL22	Mitochondrial ageing results	Houtkooper et al. 2013	9371
MRPL23	MRPL23	Mitochondrial ageing results	Houtkooper et al. 2013	7774
MRPL27	MRPL27	Mitochondrial ageing results	Houtkooper et al. 2013	10148
MRPL28	MRPL28	Mitochondrial ageing results	Houtkooper et al. 2013	6173
MRPL30	MRPL30	Mitochondrial ageing results	Houtkooper et al. 2013	10371
MRPL32	MRPL32	Mitochondrial ageing results	Houtkooper et al. 2013	4652
MRPL33	MRPL33	Mitochondrial ageing results	Houtkooper et al. 2013	9970
MRPL34	MRPL34	Mitochondrial ageing results	Houtkooper et al. 2013	6884
MRPL37	MRPL37	Mitochondrial ageing results	Houtkooper et al. 2013	5757
MRPL38	MRPL38	Mitochondrial ageing results	Houtkooper et al. 2013	5668
MRPL39	MRPL39	Mitochondrial ageing results	Houtkooper et al. 2013	2928
MRPL4	MRPL4	Mitochondrial ageing results	Houtkooper et al. 2013	4356
MRPL40	MRPL40	Mitochondrial ageing results	Houtkooper et al. 2013	9138
MRPL41	MRPL41	Mitochondrial ageing results	Houtkooper et al. 2013	9523
MRPL42	MRPL42	Mitochondrial ageing results	Houtkooper et al. 2013	4092
MRPL42P5	MRPL42P5	Mitochondrial ageing results	Houtkooper et al. 2013	NA
MRPL43	MRPL43	Mitochondrial ageing results	Houtkooper et al. 2013	4233
MRPL44	MRPL44	Mitochondrial ageing results	Houtkooper et al. 2013	7459
MRPL47	MRPL47	Mitochondrial ageing results	Houtkooper et al. 2013	7448
MRPL48	MRPL48	Mitochondrial ageing results	Houtkooper et al. 2013	7343
MRPL49	MRPL49	Mitochondrial ageing results	Houtkooper et al. 2013	6545
MRPL50	MRPL50	Mitochondrial ageing results	Houtkooper et al. 2013	3825
MRPL51	MRPL51	Mitochondrial ageing results	Houtkooper et al. 2013	7197
MRPL52	MRPL52	Mitochondrial ageing results	Houtkooper et al. 2013	5007
MRPL53	MRPL53	Mitochondrial ageing results	Houtkooper et al. 2013	11385
MRPL54	MRPL54	Mitochondrial ageing results	Houtkooper et al. 2013	10444
MRPL55	MRPL55	Mitochondrial ageing results	Houtkooper et al. 2013	3480
MRPL9	MRPL9	Mitochondrial ageing results	Houtkooper et al. 2013	5619
MRPS10	MRPS10	Mitochondrial ageing results	Houtkooper et al. 2013	5784
MRPS11	MRPS11	Mitochondrial ageing results	Houtkooper et al. 2013	8418
MRPS12	MRPS12	Mitochondrial ageing results	Houtkooper et al. 2013	9451
MRPS14	MRPS14	Mitochondrial ageing results	Houtkooper et al. 2013	10388
MRPS15	MRPS15	Mitochondrial ageing results	Houtkooper et al. 2013	11684
MRPS16	MRPS16	Mitochondrial ageing results	Houtkooper et al. 2013	11549
MRPS17	MRPS17	Mitochondrial ageing results	Houtkooper et al. 2013	2958
MRPS18A	MRPS18A	Mitochondrial ageing results	Houtkooper et al. 2013	8305
MRPS18C	MRPS18C	Mitochondrial ageing results	Houtkooper et al. 2013	10829
MRPS2	MRPS2	Mitochondrial ageing results	Houtkooper et al. 2013	2409
MRPS21	MRPS21	Mitochondrial ageing results	Houtkooper et al. 2013	3928
MRPS22	MRPS22	Mitochondrial ageing results	Houtkooper et al. 2013	10766
MRPS23	MRPS23	Mitochondrial ageing results	Houtkooper et al. 2013	8491
MRPS24	MRPS24	Mitochondrial ageing results	Houtkooper et al. 2013	5522
MRPS25	MRPS25	Mitochondrial ageing results	Houtkooper et al. 2013	2774
MRPS28	MRPS28	Mitochondrial ageing results	Houtkooper et al. 2013	3085
MRPS30	MRPS30	Mitochondrial ageing results	Houtkooper et al. 2013	6375
MRPS34	MRPS34	Mitochondrial ageing results	Houtkooper et al. 2013	7406
MRPS35	MRPS35	Mitochondrial ageing results	Houtkooper et al. 2013	2413
MRPS36	MRPS36	Mitochondrial ageing results	Houtkooper et al. 2013	11533
MRPS5	MRPS5	Mitochondrial ageing results	Houtkooper et al. 2013	8330
MRPS7	MRPS7	Mitochondrial ageing results	Houtkooper et al. 2013	8289
MTOR	FRAP1		Kenyon	7925
NBS	NBN	DNA repair genes	Key DNA repair gene	4173

NDRG1	NDRG1	MTOR/FOXO pathways	Harries et al. 2012	2646
NDUFB11	NDUFB11	Multi-tissue, multi-species candidate	de Magalhaes/Church 2009	4732
OAT	OAT	Downregulated in mice and humans w/ageing in multi-tissue analyses	Zahn et al. AGEMAP	9865
PDK1	PDK1	MTOR/FOXO pathways	Harries et al. 2012	4215
PI3K	PIK3CA	MTOR/FOXO pathways	Harries et al. 2012	7589
PRDX6	PRDX6	Upregulated in mice and humans w/ageing in multi-tissue analyses	Zahn et al. AGEMAP	5361
PTEN	PTEN	MTOR/FOXO pathways	Harries et al. 2012	7324
RHEB	RHEB	MTOR/FOXO pathways	Harries et al. 2012	10143
RPL10	NOV	Upregulated in mice and humans w/ageing in multi-tissue analyses	Zahn et al. AGEMAP	2064
RPL37	RPL37	Upregulated in mice and humans w/ageing in multi-tissue analyses	Zahn et al. AGEMAP	NA
S100A4	S100A4	Multi-tissue, multi-species candidate	de Magalhaes/Church 2009	NA
S100A6	S100A6	Multi-tissue, multi-species candidate	de Magalhaes/Church 2009	3393
SESN3	SESN3	MTOR/FOXO pathways	Harries et al. 2012	5719
SGK1	SGK	MTOR/FOXO pathways	Harries et al. 2012	3855
SIRT1	SIRT1	Longevity genetics or biology candidate	Barzilai & Gabriely 2010	3309
SIRT2	SIRT2	Longevity genetics or biology candidate	Barzilai & Gabriely 2010	2478
SLC25A5	SLC25A5	Downregulated in mice and humans w/ageing in multi-tissue analyses	Zahn et al. AGEMAP	NA
SNX3	SNX3	Downregulated in mice and humans w/ageing in multi-tissue analyses	Zahn et al. AGEMAP	10058
SOD1	SOD1	Longevity genetics or biology candidate	Barzilai & Gabriely 2010	4099
SOD2	SOD2	Longevity genetics or biology candidate	Barzilai & Gabriely 2010	2262
TEK	TEK	Longevity candidate gene	Sebastiani	5648
TFRC	TFRC	Multi-tissue, multi-species candidate	de Magalhaes/Church 2009	6258
TSC1	TSC1	MTOR/FOXO pathways	Harries et al. 2012	5045
TSC2	TSC2	MTOR/FOXO pathways	Harries et al. 2012	11259
ULK1	ULK1	MTOR/FOXO pathways	Harries et al. 2012	4350
UQCRFS1	UQCRFS1	Multi-tissue, multi-species candidate	de Magalhaes/Church 2009	11681
UQCRQ	UQCRQ	Multi-tissue, multi-species candidate	de Magalhaes/Church 2009	5502

Supplementary Table 10. The mean (absolute) transcriptomic Δage per cohort, plus the number of samples, females, and smokers.

**COHORT**	**# samples**	**# females (%)**	**# smokers (%)**	**Mean Δage** *(years)*	**Mean abs Δage** *(years)*
RS	829	446 (53.8)	226 (27.3)	0.00	6.06
InCHIANTI	681	370 (54.3)	73 (10.7)	0.00	11.21
NIDDK/PHOENIX	1,457	717 (49.2)	NA	0.00	9.05
FHS-GEN2 (OFFSPRING)	2,446	1,343 (54.9)	205 (8.4)	0.00	7.31
SHIP-TREND	970	546 (56.3)	214 (22.1)	0.00	8.63
KORA	989	493 (49.9)	66 (6.7)	0.00	4.84
EGCUT	962	482 (50.1)	243 (25.3)	0.00	9.12
DILGOM	513	276 (53.8)	100 (19.5)	0.00	7.15
**SUM**	**8,847**	**4,673 (52.8)**	**1,127 (12.7)**	**0.00**	**7.84**

Supplementary Table 11 – part 1. Associations of transcriptomic Δage with 12 ageing phenotypes in the cohorts RS, INCHIANTI, NIDDK?PHOENIX, and FHS-GEN2 - *adjusted for chronological age.*

	**RS**				**INCHIANTI**				**NIDDK/PHOENIX**				**FHS-GEN2 (OFFSPRING)**			
**Phenotype of Interest**	**Effect**	**SE**	**P**	**n**	**Effect**	**SE**	**P**	**n**	**Effect**	**SE**	**P**	**n**	**Effect**	**SE**	**P**	**n**
Sex: *0 = male, 1 = female*	-0.0020	0.0026	4.32E-01	829	-0.0019	0.0015	2.12E-01	681	0.0007	0.0013	6.07E-01	1,457	-0.0049	0.0013	1.14E-04	2,440
Systolic blood pressure: *mmHg*	0.5424	0.1221	1.06E-05	572	0.1064	0.0585	6.96E-02	679	0.2110	0.0377	2.59E-08	1,457	0.2267	0.042	8.48E-08	2,438
Diastolic blood pressure: *mmHg*	0.3526	0.0706	7.77E-07	572	0.0338	0.0329	3.05E-01	679	0.1330	0.0259	3.19E-07	1,457	0.0706	0.025	4.84E-03	2,435
Total cholesterol levels: *mmol/L*	0.0017	0.0058	7.68E-01	691	0.4370	0.1208	3.20E-04	681	0.0065	0.0030	2.71E-02	1,447	0.3251	0.095	6.21E-04	2,440
HDL cholesterol levels: *mmol/L*	0.0012	0.0024	6.02E-01	691	0.1021	0.0465	2.85E-02	681	0.0050	0.0010	1.73E-06	1,447	0.1336	0.046	3.58E-03	2,439
Fasting glucose levels: *mmol/L*	0.0165	0.0057	3.96E-03	826	0.0058	0.0041	1.52E-01	681	0.0314	0.0081	1.05E-04	1,430	0.1941	0.061	1.51E-03	2,439
Body Mass Index: *kg/m2*	0.0706	0.0243	3.72E-03	829	0.0104	0.0132	4.31E-01	681	0.0081	0.0190	6.72E-01	1,450	0.0462	0.014	8.05E-04	2,440
Waist Hip Ratio	0.0008	0.0004	5.48E-02	819	0.0001	0.0002	7.57E-01	591	na	na	na	na	na	na	na	na
Hand grip strength: *kg*	0.0135	0.0569	8.12E-01	556	0.0295	0.0312	3.44E-01	673	na	na	na	na	-0.0701	0.028	1.36E-02	2,422
Renal function	0.0377	0.0842	6.55E-01	827	0.0925	0.0614	1.32E-01	674	0.0751	0.0453	9.80E-02	1,445	-0.0424	0.045	3.47E-01	2,439
Mini Mental State Exam Score	-0.0064	0.0099	5.23E-01	817	-0.0183	0.0147	2.12E-01	675	na	na	na	na	na	na	na	na
Current smoking: *0 = no, 1 = yes*	0.0057	0.0023	1.37E-02	829	-0.0008	0.0009	4.13E-01	681	na	na	na	na	0.0033	0.0007	3.19E-06	2,440

Supplementary Table 11 – part 2. Associations of transcriptomic Δage with 12 ageing phenotypes in the cohorts SHIP-TREND, KORA, EGCUT, and DILGOM - *adjusted for chronological age.*

	**SHIP-TREND**				**KORA**				**EGCUT**				**DILGOM**			
**Phenotype of Interest**	**Effect**	**SE**	**P**	**n**	**Effect**	**SE**	**P**	**n**	**Effect**	**SE**	**P**	**n**	**Effect**	**SE**	**P**	**n**
Sex: *0 = male, 1 = female*	-0.0001	0.0016	9.63E-01	970	0.0007	0.003	8.33E-01	984	-0.0021	0.001	1.55E-01	962	0.0006	0.0025	8.05E-01	513
Systolic bloodpressure: *mmHg*	0.2131	0.0514	3.62E-05	969	0.2752	0.127	3.03E-02	983	0.0450	0.048	3.47E-01	962	0.2227	0.0884	1.21E-02	511
Diastolic bloodpressure: *mmHg*	0.1316	0.0313	2.90E-05	969	0.0187	0.062	7.64E-01	983	0.0404	0.031	1.89E-01	962	0.1236	0.0504	1.46E-02	511
Total cholesterol levels: *mmol/L*	0.0101	0.0033	2.23E-03	970	-0.0031	0.007	6.38E-01	984	0.0058	0.003	6.77E-02	962	0.0028	0.0048	5.60E-01	513
HDL cholesterol levels: *mmol/L*	0.0016	0.0012	1.73E-01	970	-0.0018	0.002	4.28E-01	984	0.0006	0.001	6.77E-01	962	-0.0009	0.0019	6.22E-01	513
Fasting glucose levels: *mmol/L*	0.0043	0.0019	2.30E-02	970	0.4725	0.149	1.56E-03	984	Na	na	na	na	na	na	na	na
Body Mass Index: *kg/m2*	0.0335	0.0143	1.89E-02	970	0.0804	0.029	5.18E-03	984	0.0031	0.013	8.19E-01	962	0.0530	0.0228	2.06E-02	513
Waist Hip Ratio	0.0003	0.0003	2.44E-01	970	0.0011	0.001	2.52E-02	984	0.0003	0.000	2.65E-01	962	0.0006	0.0004	1.63E-01	511
Hand grip strength: *kg*	na	na	na	na	na	na	na	na	Na	na	na	na	na	na	na	na
Renal function	0.0573	0.0616	3.52E-01	970	na	na	na	na	-0.0382	0.052	4.65E-01	962	na	na	na	na
Mini Mental State Exam Score	na	na	na	na	na	na	na	na	Na	na	na	na	na	na	na	na
Current smoking: *0 = no, 1 = yes*	0.0117	0.0012	2.14E-20	970	0.0025	0.002	1.07E-01	984	-0.0058	0.001	7.33E-06	962	-0.0005	0.0020	7.86E-01	513

We tested whether the transcriptomic delta age was associated with twelve different phenotypes known to be associated with chronological age. Gene expression levels were adjusted for plate ID, RNA quality score, fasting state, sex, smoking status, and cell counts. Results are presented per cohort, and these results were the basis for Table 2, which shows the results of the meta-analysis across all cohorts.

Supplementary Table 12 – part 1. Associations of transcriptomic Δage with 12 ageing phenotypes in the cohorts RS, INCHIANTI, NIDDK/PHOENIX, and FHS-GEN2 - *adjusted for chronological age and BMI.*

	RS				INCHIANTI				NIDDK/PHOENIX				FHIS-GEN2 (OFFSPRING)			
**Phenotype of Interest**	**Effect**	**SE**	**P**	**n**	**Effect**	**SE**	**P**	**n**	**Effect**	**SE**	**P**	**n**	**Effect**	**SE**	**P**	**n**
Sex: *0 = male, 1 = female*	-0.0019	0.0026	4.60E-01	829	-0.0018	0.0015	2.33E-01	681	0.0006	0.001	6.30E-01	1,450	-0.0054	0.0013	2.18E-05	2,440
Systolic bloodpressure: *mmHg*	0.5120	0.1219	3.11E-05	572	0.1007	0.0582	8.37E-02	679	0.2100	0.038	2.38E-08	1,450	0.2120	0.042	5.03E-07	2,438
Diastolic bloodpressure: *mmHg*	0.3230	0.0697	4.41E-06	572	0.0276	0.0320	3.90E-01	679	0.1310	0.026	4.13E-07	1,450	0.0645	0.025	9.94E-03	2,435
Total cholesterol levels: *mmol/L*	0.0020	0.0058	7.28E-01	691	0.4334	0.1209	3.61E-04	681	0.0067	0.003	2.42E-02	1,440	0.3699	0.094	8.79E-05	2,440
HDL cholesterol levels: *mmol/L*	0.0030	0.0023	1.89E-01	691	0.1129	0.0445	1.15E-02	681	0.0051	0.001	4.03E-07	1,440	0.1872	0.043	1.42E-05	2,439
Fasting glucose levels: *mmol/L*	0.0107	0.0054	4.75E-02	826	0.0052	0.0040	1.91E-01	681	0.0311	0.008	1.06E-04	1,423	0.1453	0.059	1.46E-02	2,439
Body Mass Index: *kg/m2*	na	na	na	na	na	na	na	na	na	na	na	na	na	na	na	na
Waist Hip Ratio	0.0003	0.0004	4.30E-01	819	0.0001	0.0002	7.26E-01	591	na	na	na	na	na	na	na	na
Hand grip strength: *kg*	0.0134	0.0571	8.14E-01	556	0.0253	0.0308	4.12E-01	673	na	na	na	na	-0.0769	0.028	6.77E-03	2,422
Renal function	0.0418	0.0847	6.22E-01	827	0.0720	0.0494	1.45E-01	674	0.0757	0.045	9.53E-02	1,438	-0.0430	0.045	3.42E-01	2,439
Mini Mental State Exam Score	-0.0064	0.0100	5.21E-01	817	-0.0197	0.0146	1.78E-01	675	na	na	na	na	na	na	na	na
Current smoking: *0 = no, 1 = yes*	0.0063	0.0023	6.40E-03	829	-0.0007	0.0009	4.34E-01	681	na	na	na	na	0.0034	0.0007	1.19E-06	2,440

Supplementary Table 12 – part 2. Associations of transcriptomic Δage with 12 ageing phenotypes in the cohorts SHIP-TREND, KORA, EGCUT, and DILGOM - *adjusted for chronological age and BMI.*

	SHIP-TREND				KORA				EGCUT				DILGOM			
**Phenotype of Interest**	**Effect**	**SE**	**P**	**n**	**Effect**	**SE**	**P**	**n**	**Effect**	**SE**	**P**	**n**	**Effect**	**SE**	**P**	**n**
Sex: *0 = male, 1 = female*	0.0003	0.0016	8.58E-01	970	0.0000	0.003	9.99E-01	984	-0.0021	0.001	1.60E-01	962	0.0007	0.0026	7.72E-01	513
Systolic bloodpressure: *mmHg*	0.1834	0.0498	2.45E-04	969	0.2732	0.127	3.23E-02	983	0.0412	0.045	3.58E-01	962	0.1934	0.0880	2.85E-02	511
Diastolic bloodpressure: *mmHg*	0.1068	0.0295	3.04E-04	969	0.0173	0.063	7.83E-01	983	0.0378	0.029	1.86E-01	962	0.1030	0.0500	3.97E-02	511
Total cholesterol levels: *mmol/L*	0.0096	0.0033	3.77E-03	970	-0.0018	0.007	7.79E-01	984	0.0057	0.003	7.00E-02	962	0.0019	0.0048	7.01E-01	513
HDL cholesterol levels: *mmol/L*	0.0027	0.0011	1.62E-02	970	-0.0001	0.002	9.78E-01	984	0.0007	0.001	5.97E-01	962	0.0003	0.0018	8.58E-01	513
Fasting glucose levels: *mmol/L*	0.0031	0.0018	9.10E-02	970	0.3707	0.145	1.08E-02	984	na	na	na	na	na	na	na	na
Body Mass Index: *kg/m2*	na	na	na	na	na	na	na	na	na	na	na	na	na	na	na	na
Waist Hip Ratio	0.0001	0.0003	8.04E-01	970	0.0008	0.000	1.23E-01	984	0.0003	0.000	2.52E-01	962	0.0002	0.0004	5.96E-01	511
Hand grip strength: *kg*	na	na	na	na	na	na	na	na	na	na	na	na	na	na	na	na
Renal function	0.0710	0.0615	2.49E-01	970	na	na	na	na	-0.0375	0.052	4.73E-01	962	na	na	na	na
Mini Mental State Exam Score	na	na	na	na	na	na	na	na	na	na	na	na	na	na	na	na
Current smoking: *0 = no, 1 = yes*	0.0117	0.0012	2.75E-20	970	0.0029	0.002	6.57E-02	984	-0.0058	0.001	7.45E-06	962	-0.0005	0.0020	8.23E-01	513

We tested whether the transcriptomic delta age was associated with twelve different phenotypes known to be associated with chronological age. Gene expression levels were adjusted for plate ID, RNA quality score, fasting state, sex, smoking status, and cell counts. Results are presented per cohort, and these results were the basis for Table 2, which shows the results of the meta-analysis across all cohorts.

Supplementary Table 13. Association of transcriptomic Δage (green), Horvath's epigenetic Δage (red), and Hannum's epigenetic Δage (blue) with ageing phenotypes – *adjusted for chronological age, sex, and BMI.*

Phenotype of Interest	n	TC-Effect	TC-SE	TC-P	Dir	HO-Effect	HO-SE	HO-P	Dir	HA-Effect	HA-SE	HA-P	Dir
**Systolic bloodpressure:** ***SBP (mmHg)***	1,197	**0.306**	**0.095**	**1.30E-03**	++	-0.012	0.080	8.80E-01	-+	0.087	0.103	4.02E-01	++
Diastolic bloodpressure: *DBP (mmHg)*	1,197	0.104	0.051	4.32E-02	+-	-0.059	0.042	1.61E-01	--	-0.028	0.055	6.15E-01	+-
Total cholesterol levels: *TC (mmol/L)*	1,286	-0.001	0.005	8.71E-01	+-	-0.002	0.004	6.19E-01	--	-0.010	0.005	6.26E-02	--
HDL cholesterol levels: *HDL (mmol/L)*	1,286	0.003	0.002	1.25E-01	++	-0.002	0.001	1.04E-01	--	-0.003	0.002	1.15E-01	+-
Fasting glucose levels: *FG (mmol/L)*	1,394	0.011	0.006	5.30E-02	++	-0.003	0.005	5.42E-01	-+	**0.013**	**0.006**	**3.49E-02**	++
**WHR**	1,388	**0.001**	**0.000**	**2.13E-02**	++	**0.001**	**<0.001**	**1.33E-02**	++	**0.001**	**<0.001**	**2.51E-03**	++
**Current smoking:** ***0 = no, 1 = yes***	1,396	**0.005**	**0.002**	**7.92E-04**	++	0.001	0.001	3.71E-01	++	**0.004**	**0.002**	**5.87E-03**	++

Gene expression levels were adjusted for plate ID, RNA quality score, fasting state, sex, smoking status, and cell counts. TC = transcriptomic predictor, HO = Horvath predictor, HA = Hannum predictor, Dir = direction of the effect, n = sample size of the meta-analysis, EV = explained variance, RS = Rotterdam Study, KORA = The KORA Study, BMI = Body mass index, WHR = Waist-hip-ratio.

Supplementary Table 14. The explained variance using the transcriptomic predictor (green), the Horvath's epigenetic predictor (red), and the Hannum's epigenetic predictor (blue) of the ageing phenotypes – *adjusted for chronological age, sex, and BMI*

Phenotype of Interest	n	TC-EV-RS	TC-EV-KORA	HO-EV-RS	HO-EV-KORA	HA-EV-RS	HA-EV-KORA
Systolic bloodpressure: *SBP (mmHg)*	1,197	0.022	0.001	0.000	0.000	0.000	0.000
Diastolic bloodpressure: *DBP (mmHg)*	1,197	0.033	0.000	0.000	0.000	0.000	0.000
Total cholesterol levels: *TC (mmol/L)*	1,286	0.000	0.001	0.000	0.000	0.002	0.001
HDL cholesterol levels: *HDL (mmol/L)*	1,286	0.000	0.000	0.000	0.005	0.000	0.012
Fasting glucose levels: *FG (mmol/L)*	1,394	0.007	0.007	0.000	0.007	0.007	0.003
WHR	1,388	0.004	0.015	0.002	0.005	0.012	0.006
Current smoking: *0 = no, 1 = yes*	1,396	0.010	0.003	0.002	0.000	0.009	0.001

Gene expression levels were adjusted for plate ID, RNA quality score, fasting state, sex, smoking status, and cell counts. TC = transcriptomic predictor, HO = Horvath predictor, HA = Hannum predictor, n = sample size of the meta-analysis, EV = explained variance, RS = Rotterdam Study, KORA = The KORA Study, BMI = Body mass index, WHR = Waist-hip-ratio.

Supplementary Table 15. Combining the transcriptomic Δage and Horvath's epigenetic Δage in one model – associations of both predictors with the ageing phenotypes - *adjusted for chronological age, sex, and BMI*

Phenotype of Interest	n	TC-Effect	TC-SE	TC-P	Dir	HO-Effect	HO-SE	HO-P	Dir	EV-RS	EV-KORA
**Systolic bloodpressure: *SBP (mmHg)***	1,197	**0.320**	**0.097**	**9.31E-04**	**++**	-0.059	0.080	4.66E-01	-+	0.023	0.000
Diastolic bloodpressure: *DBP (mmHg)*	1,197	0.121	0.052	2.01E-02	+-	-0.076	0.043	7.48E-02	--	0.038	0.000
Total cholesterol levels: *TC (mmol/L)*	1,286	<0.001	0.005	9.54E-01	+-	-0.002	0.004	6.27E-01	-+	0.000	0.000
HDL cholesterol levels: *HDL (mmol/L)*	1,286	0.003	0.002	6.87E-02	++	-0.003	0.001	5.02E-02	--	0.000	0.004
**Fasting glucose levels: *FG (mmol/L)***	1,394	**0.012**	**0.006**	**3.24E-02**	**++**	-0.006	0.005	2.81E-01	-+	0.008	0.012
**WHR**	1,388	<0.001	<0.001	5.91E-02	++	**<0.001**	**<0.001**	**3.68E-02**	**++**	0.004	0.017
**Current smoking: *0 = no, 1 = yes***	1,396	**0.005**	**0.002**	**1.45E-03**	**++**	0.000	0.001	7.80E-01	+-	0.010	0.001

Gene expression levels were adjusted for plate ID, RNA quality score, fasting state, sex, smoking status, and cell counts. TC = transcriptomic predictor, HO = Horvath predictor, n = sample size of the meta-analysis, EV = explained variance, RS = Rotterdam Study, KORA = The KORA Study, BMI = Body mass index, WHR = Waist-hip-ratio.

Supplementary Table 16. Combining the transcriptomic Δage and Hannum's epigenetic Δage in one model – associations of both predictors with the ageing phenotypes - *adjusted for chronological age, sex, and BMI*

Phenotype of Interest	n	TC-Effect	TC-SE	TC-P	Dir	HA-Effect	HA-SE	HA-P	Dir	EV-RS	EV-KORA
**Systolic bloodpressure: *SBP (mmHg)***	1,197	**0.309**	**0.100**	**1.89E-03**	**++**	-0.003	0.107	9.76E-01	-+	0.020	0.000
Diastolic bloodpressure: *DBP (mmHg)*	1,197	0.123	0.054	2.17E-02	+-	-0.056	0.058	3.29E-01	--	0.032	0.000
Total cholesterol levels: *TC (mmol/L)*	1,286	0.002	0.005	6.74E-01	+-	-0.010	0.005	6.36E-02	--	0.003	0.001
**HDL cholesterol levels: *HDL (mmol/L)***	1,286	**0.004**	**0.002**	**3.23E-02**	**++**	**-0.004**	**0.002**	**3.54E-02**	**--**	0.000	0.011
Fasting glucose levels: *FG (mmol/L)*	1,394	0.008	0.006	1.50E-01	++	0.011	0.006	9.63E-02	++	0.011	0.007
**WHR**	1,388	<0.001	<0.001	1.34E-01	++	**0.001**	**<0.001**	**1.62E-02**	**++**	0.012	0.016
**Current smoking: *0 = no, 1 = yes***	1,396	**0.004**	**0.002**	**7.34E-03**	**++**	0.003	0.002	6.00E-02	++	0.015	0.002

Gene expression levels were adjusted for plate ID, RNA quality score, fasting state, sex, smoking status, and cell counts. TC = transcriptomic predictor, HA = Hannum predictor, n = sample size of the meta-analysis, EV = explained variance, RS = Rotterdam Study, KORA = The KORA Study, BMI = Body mass index, WHR = Waist-hip-ratio.

Supplementary Table 17. Description of the discovery cohorts.

Discovery Cohort	Sample size	Primary ancestry	Cell type	Age (mean)	Fasting	Type of Cohort	Covariates Included
EGCUT	1,086	European	Whole blood	38.2	Y	Population-Based	cell counts, plateID, RQS, sex, smoking status, fasting status
FHS - OFFSPRING *(GEN 2: EXAM 8)*	2,608	European-American	Whole blood	66.4	Y	Family-Based	plateID, RQS, sex, smoking status, fasting status, family design
INCHIANTI	698	European	Whole blood	72.2	N	Population-Based	cell counts, plateID, sex, smoking status
KORA	993	European	Whole blood	70.4	Y	Population-Based	#platelets, plateID, RQS, sex, smoking status, fasting status
RS-III	881	European	Whole blood	59.8	Y	Population-Based	cell counts, plateID, RQS, sex, smoking status, fasting status
SHIP-TREND	991	European	Whole blood	50.2	Y	Population-Based	cell counts, plateID, RQS, sex, smoking status, fasting status

The sample size, the primary ancestry, the cell type, the mean age, the fasting status, the type of cohort, and the covariates used in the linear regression analysis.

Supplementary Table 18. Description of the replication cohorts.

Replication Cohort	Sample size	Primary ancestry	Cell type	Age (mean)	Fasting	Type of Cohort	Covariates Included
BSGS	962	European-Australian	Whole blood	20.1 *(bi-model distribution)*	Y	Family-Based	cellcounts, plateID, RQS, sex, fasting status
DILGOM	512	European	Whole blood	51.5	Y	Population-Based	sex, fasting status
FEHRMANN	1,191	European	Whole blood	62.8	N	COPD/ALS cases & controls	Sex
FHS - GEN 3 *(GEN 3: EXAM 2)*	3,180	European-American	Whole blood	46.4	Y	Family-Based	cellcounts, plateID, RQS, sex, smoking status, fasting status, family design
GTP	359	African-American	Whole blood	42.5	Y	Population-Based	plateID, array version, sex, RQS
HVHv3	121	European-American	Whole blood	68.4	N	MI/STROKE/AF cases & controls	cellcounts, plateID, RQS, sex
HVHv4	227	European-American	Whole blood	68.7	N	MI/STROKE/AF cases & controls	cellcounts, plateID, RQS, sex
NIDDK/PHOENIX	1,457	Native American	Whole blood	35.9	Y	Population-Based	plateID, sex

The sample size, the primary ancestry, the cell type, the mean age, the fasting status, the type of cohort, and the covariates used in the linear regression analysis.

Supplementary Table 19. Description of the generalization cohorts.

Generalization Cohort	Sample size	Primary ancestry	Cell type	Age (mean)	Fasting	Type of Cohort	Covariates Included
BOSTON COHORT-CD4+	213	Multi-ethnic	CD4+ cells	28.4	na	Population-Based	sex
EGCUT-CD4+	302	European	CD4+ cells	53.1	na	Population-Based	plateID, sex, smoking status, amount of RNA per sample
EGCUT-CD8+	299	European	CD8+ cells	52.6	na	Population-Based	plateID, sex, smoking status, amount of RNA per sample
BOSTON COHORT-CD14+	213	Multi-ethnic	CD14+ cells	28.4	na	Population-Based	sex
MESA-MONOCYTES	354	Multi-ethnic	Monocytes	70.2	na	Atherosclerosis cases & controls	cellcounts, plateID, RQS, smoking status, fasting status
NABEC-UKBEC-CB	394	European	Cerebellum	51.3	na	Autopsy brain samples: no recognisable neurological disease	batch, sex, postmortem interval, 2 Principal Components
NABEC-UKBEC-FC	394	European	Frontal cortex	51.3	na	Autopsy brain samples: no recognisable neurological disease	batch, sex, postmortem interval, 2 Principal Components
GENOA-LCLs	869	Multi-ethnic	LCLs	59.0	na	Hypertension Siblings	plateID, RQS, sex, smoking status
SAFS-LYMPHOCYTES	1,244	Hispanic	Lymphocytes	39.3	Y	Family-Based	sex, family design
GARP-PBMCs	134	European	PBMC	60.1	N	Osteoarthritis cases & controls	plateID, sex, smoking status, fasting status
PBMC-MS	228	Multi-ethnic	PBMC	40.9	N	MS cases & controls: treated with IFN-b, glatiramer acetate or untreated	sex

The sample size, the primary ancestry, the cell type, the mean age, the fasting status, the type of cohort, and the covariates used in the linear regression analysis.

Supplementary Table 20. Direct associations between chronological age and the ageing phenotypes.

	META-ANALYIS			
**Phenotype of Interest**	**Zscore**	**P**	**Direction**	**n**
Sex: *0 = male, 1 = female*	0.585	5.59E-01	++-++-+-	8,836
Systolic bloodpressure: *mmHg*	27.317	2.66E-164	++++++++	8,571
Diastolic bloodpressure: *mmHg*	-1.605	1.08E-01	+-+-+-++	8,568
Total cholesterol levels: *mmol/L*	5.491	3.99E-08	+-+-+-++	8,688
HDL cholesterol levels: *mmol/L*	-1.247	2.12E-01	+-+-----	8,687
Fasting glucose levels: *mmol/L*	13.292	2.58E-40	++++++??	7,330
Body Mass Index: *kg/m2*	8.238	1.75E-16	+++-++++	8,829
Waist Hip Ratio	15.490	4.05E-54	++??++++	4,837
Hand grip strength: *kg*	-23.141	1.79E-118	--?-????	3,651
Renal function	-42.159	2.14e-388	-----?-?	7,317
Mini Mental State Exam Score	-13.397	6.32E-41	--??????	1,492
Current smoking: *0 = no, 1 = yes*	-11.587	4.81E-31	--?---+-	7,379

We tested whether chronological age was associated with the twelve phenotypes known to be associated with chronological age. Gene expression levels were adjusted for plate ID, RNA quality score, fasting state, sex, smoking status, and cell counts. Results were meta-analyzed over all cohorts.

Supplementary Table 21. Calculation of mean age and the pooled variance over all samples in the meta-analysis.

COHORT	MEAN AGE	SD AGE	s2 (variance)	n	n-1	(n-1)*s2
DILGOM	51.90	13.70	187.69	512	511	95909.59
EGCUT	38.22	15.76	248.38	1,086	1,085	269489.70
FHS-OFFSPRING	66.40	8.95	80.10	2,446	2,445	195850.61
INCHIANTI	72.23	15.27	233.17	695	694	161821.99
KORA	70.38	5.37	28.79	993	992	28563.60
NIDDK/PHOENIX	35.90	12.30	151.29	1,457	1,456	220278.24
RS	59.80	8.10	65.61	875	874	57343.14
SHIP-TREND	50.59	13.72	188.35	991	990	186464.69
**MEAN AGE**	**55.81**					
**POOLED s2(VARIANCE)**	**134.38**					
**POOLED SD**	**11.59**					

We calculated the pooled variance using the mean age, the standard deviation, and the sample size, as described on http://en.wikipedia.org/wiki/Pooled_variance

SUPPLEMENTARY NOTE 1

Descriptions of the participating study populations

Brisbane Systems Genetics Study (BSGS)

Individuals present in this study were recruited as part of the Brisbane Twin Nevus and cognition studies (known as BTN and MAPS respectively). As described in detail elsewhere[1], adolescent MZ and DZ twins, their siblings, and their parents have been recruited over a 16 year period into an ongoing study of the genetic and environmental factors influencing pigmented nevi and the associated risk of developing skin cancer and cognition. The sample is of northern European origin (mainly Anglo-Celtic). A Principal Component Analysis (PCA) comparing individuals in this study to HapMap3 (International HapMap3 Consortium) and GenomEUtwin[2] populations shows the close similarity of ancestry to northern European populations. All participants gave informed consent, and the appropriate institutional review boards approved the study protocol. Whole blood for expression profiling was collected directly into PAXGene ™ tube (QIAGEN, Valencia, CA). Total RNA was extracted from PAXGene™ tubes using the WB gene RNA purification kit (QIAGEN, Valencia, CA). RNA from all samples was run on an Agilent Bioanalyzer to assess quality and to estimate RNA concentrations RNA was converted to cDNA, amplified and purified using the Ambion Illumina TotalPrep RNA Amplification Kit (Ambion).

Expression profiles were generated by hybridising 750 ng of cRNA to Illumina HumanHT-12 v4.0 Beadchip according to Illumina whole-genome gene expression direct hybridization assay guide (Illumina Inc, San Diego, USA). Briefly, 500 ng of total RNA were used to generate biotinylated cRNA, which was fragmented and hybridised to an Illumina whole genome expression chip, HumanHT-12 v4.0 for 18 h at 58°C. Beadchips were then washed and stained and subsequently scanned to obtain fluorescence intensities. Samples were scanned using an Illumina Bead Array Reader. Samples were randomized across chips and chip positions, with checks for balance across families, sex and generation. Full details of the BSGS cohort and sample preparation are given elsewhere[3,4]. The expression data is available at GEO (Gene Expression Omnibus) public repository under the accession GSE33321.

DNA methylation was measured on 614 individuals from 117 families of European descent recruited as part of BSGS[5]. Bisulfite conversions were performed in 96 well plates using the EZ-96 DNA Methylation Kit (Zymo Research, Irvine, CA, USA). Prior to conversion, DNA concentrations were determined by NanoDrop quantification (NanoDrop Technologies, Inc., Wilmington, DE, USA) and standardised to include 500ng. Three technical replicates were included in each conversion to assess repeatability. A commercial female human genomic DNA sample (Promega Corporation, Madison, WI, USA) was used on all plates, one sample from each run was duplicated on the plate and one sample duplicated from a different plate. DNA recovery after conversion was quantified using Nanodrop (Thermo Scientific, Wilmington, DE, USA).

Bisulfite converted DNA samples were hybridised to the 12 sample, Illumina HumanMethylation450 BeadChips using the Infinium HD Methylation protocol and Tecan robotics (Illumina, San Diego, CA, USA). The HM 450 BeadChip-assessed methylation status was interrogated at 485,577 CpG sites across the genome. It provides coverage of 99% of RefSeq genes. Methylation scores for each CpG site are obtained as a ratio of the intensities of fluorescent signals and are represented as β-values. Samples were randomly placed with respect to the chip they were measured on and to the position on that chip in order to avoid any confounding with family. Box-plots of the red and green intensity levels and their ratio were used to ensure that no chip position was under- or over-exposed, with any outlying samples repeated. Similarly, the proportion of probes with detection p-value less than 0.01 was examined to confirm strong binding of the sample to the array.

Methylation probes on the sex chromosomes or having been annotated as binding to multiple chromosomes were removed from the analysis, as were those with zero CpG sites. The probability of a probe within a sample either being called as missing or with a detection p-value less than 0.001 were estimated from the average rate across all probes and samples. A threshold for probes showing significant deviation from random missingness (or excess poor binding) was determined by testing against a binomial distribution for the number of samples at the 0.05 significance level with a Bonferroni correction for the number of probes. Any probe with more than 11 individuals with missing data or more than five individuals with detection p-values >

0.001 were removed. After cleaning a total of 417,069 probes remained.

Dietary, Lifestyle, and Genetic determinants of Obesity and Metabolic syndrome study (DILGOM)

The Finnish study samples included a total of 513 unrelated individuals aged 25–74 years from the Helsinki area, recruited during 2007 as part of the DILGOM study, an extension of the FINRISK 2007 study described earlier[6]. Study participants were asked to fast overnight (at least 10 hours) prior to giving a blood sample.

To obtain stabilized total RNA, we used the PAXGene Blood RNA System (PreAnalytiX GMbH, Hombrechtikon, Switzerland). It included collection of 2.5 ml peripheral blood into PAXGene Blood RNA Tubes (Becton Dickinson and Co., Franklin Lakes, NJ, USA) and total RNA extraction with PAXGene Blood RNA Kit (Qiagen GmbH, Hilden, Germany). Protocol recommended by the manufacturer was used. The integrity and quantity of the RNA samples were evaluated with the 2100 Bioanalyzer (Agilent Technologies, Santa Clara, CA, USA). Biotinylated cRNA was produced from 200 ng of total RNA with Ambion Illumina TotalPrep RNA Amplification Kit (Applied Biosystems, Foster City, CA, USA), using the protocol specified by the manufacturer. 750 ng of biotinylated cRNA were hybridized onto Illumina HumanHT-12v3 Expression BeadChips (Illumina Inc., San Diego, CA, USA), using standard protocol. After sample mix-up correction, 509 samples were included for further analysis in this cohort. The expression data is available at ArrayExpress public repository under the accession E-TABM-1036.

Estonian Gene Expression Cohort (EGCUT)

The Estonian Gene Expression Cohort is composed of 1,124 randomly selected samples (mean age 37.8 years) from the Estonian Genome Center, University of Tartu (EGCUT, www.biobank.ee). The EGCUT is the population based database which comprises currently the health, genealogical and genome data of more than 51,530 individuals 18 years of age and up reflecting closely the age distribution in the adult Estonian population. Participants of EGCUT recruited by the general practitioners (GP) from GP offices, physicians from the hospitals or data collectors from EGCUT's patient recruitment offices. Each participant filled out a Computer Assisted Personal interview during 1-2 hours at a doctor's or data collector's office, including personal data (place of birth, place(s) of living, nationality etc.), genealogical data (family history, three generations), educational and occupational history and lifestyle data (physical activity, dietary habits, smoking, alcohol consumption, quality of life). Anthropometric and physiological measurements were also taken. All diseases are defined according to the ICD10 coding. Women filled out additional questionnaire relating to the woman's health.

The collection of blood samples and the data is conducted according to the Estonian Gene Research Act and all participants have signed the broad informed consent. For gene expression profiling, whole blood samples were collected in Tempus Blood RNA Tubes (Life Technologies, NY, USA). RNA was extracted using Tempus Spin RNA Isolation Kit (Life Technologies, NY, USA) and quantified by NanoDrop 1000 Spectrophotometer (Thermo Fisher Scientific, DE, USA). RNA integrity was assessed by Agilent 2100 Bioanalyzer (Agilent Technologies, CA, USA). Samples with RIN<7.0 were excluded. Following amplification and labelling of RNA by Ambion TotalPrep RNA Amplification Kit (Life Technologies, NY, US), the whole genome gene expression levels were obtained by Illumina HT12v3 arrays (Illumina Inc, San Diego, US) according manufactures protocols. 1,086 samples passed the general quality control and were included in the subsequent analyses. The expression data is available at GEO (Gene Expression Omnibus) public repository under the accession GSE48348.

Methylation data pre-processing and quality control analyses were performed in R using the Bioconductor package minfi[7] . 'Raw' pre-processing was used to convert the intensities from the red and the green channels into methylated and unmethylated signals. Beta values were computed using Illumina's formula [beta = M/(M+U+100)]. The difference in the distribution of beta values for type I and type II probes was corrected for using the SWAN normalization method[8]. Detection p-values were obtained for every CpG probe in every sample. Failed positions were defined as signal levels lower than background from both the methylated and unmethylated channels. Samples with high proportion of failed position were discarded from further analyses.

For the purification of CD4+ and CD8+ T-cells, we obtained peripheral blood from healthy donors of the Estonian Genome Center of the University of Tartu (Tserel *et al.*, in press). Peripheral blood

mononuclear cells (PBMC) were extracted using Ficoll-Paque (GE Healthcare) gradient centrifugation. $CD4^+$ T cells and $CD8^+$ T cells were extracted from the PBMCs by consecutive positive separation using microbeads (CD4+ #130-045-101; CD8+ #130-045-201) and AutoMACS technology (Miltenyi Biotec) according to the manufacturer's protocol. RNA was extracted using the miRNeasy Mini Kit combined with a recommended RNase-free DNase I treatment (both from Qiagen) according to the manufacturer's protocol. The RNA was labeled and amplified using the TargetAmp Nano Labeling Kit for Illumina Expression BeadChip (Epicentre Biotechnologies) with SuperScript III Reverse Transcriptase (Life Technologies) according to the manufacturer's protocol, followed by purification with the RNeasy MinElute Cleanup Kit (Qiagen). The labelled RNA samples were hybridized to HumanHT-12 v4 Expression BeadChips (Illumina) according to the manufacturer's instructions.

Fehrmann et al. dataset (FEHRMANN)

The Fehrmann dataset consists of whole peripheral blood samples of 1,469 unrelated individuals from the United Kingdom and the Netherlands[9,10]. Some of these individuals are patients, while others are healthy controls. RNA levels were quantified using both the Illumina H8v2 platform (229 samples) and the HT12v3 platform (1,240 samples), as has been described before. The total number of samples having gene-expression data and age equals 1,191. The expression data is available at GEO (Gene Expression Omnibus) public repository under the accessions GSE20332 and GSE20142.

Framingham Heart Study (FHS)

The Framingham Heart Study (FHS) is a community-based, prospective, longitudinal study of three generations of participants. The original cohort enrolled 5,209 participants starting in 1948 and the Offspring (Second Generation) cohort enrolled 5,124 children and spouses of children of the original cohort starting in 1971[11]. From 2002 to 2005 the grandchildren of the original cohort were enrolled in the Third Generation cohort. Participants gave informed consent for research and the study is approved by the Boston University IRB. Participants received clinic visits examinations over time including detailed medical histories, standard exam measurements and had fasting blood samples collected.

Fasting whole blood samples (PAXGene Tubes-Becton Dickinson) were collected from the FHS OFFSPRING (at their 8th examination cycle), and the FHS 3rd GENERATION (at their 2nd examination cycle). Details of the cohort designs and ascertainments have previously been described[39]. RNA was extracted from whole blood samples following the manufacturers' protocol (PAXGene Blood RNA kit - Assuragen). 50 ng of total RNA were amplified following the NuGen WG-Ovation Pico RNA amplification system and labeling conducted with the FL-Ovation cDNA Biotin Module V2 (NuGen, San Carlos, CA). Fragmented, biotinylated cDNA (5.5ug) was prepared for each sample and added to a hybridization cocktail and loaded onto an Affymetrix Human Exon 1.0 ST GeneChip (1.4 million probesets), and hybridized at 45^0C and 60 rpm for 16h. The manufacturer's protocol was followed for washing and staining, and stained arrays were scanned at 532 nm in an Affymetrix GeneChip Scanner 3000 to generate CEL files for each array. The expression data is available at dbGAP public repository under the accession phs000363.v7.p8.

Genetics, osteoARthritis and Progression (GARP)

The GARP study is a study among Dutch Caucasian sib pairs with symptomatic osteoarthritis at multiple joint locations: the GARP study consists of 191 sib pairs (N=382) of white, Dutch, ancestry. All participants (age-range: 40-70 years; mean age: 60 years) are clinically and radiographically diagnosed with primary, symptomatic osteoarthritis at multiple joint sites in the hand, or in at least two joints of the following locations: hand, spine, knee, or hip[12].

For the current study, whole genome expression profiles were analyzed from 108 participants (68 unrelated families) of the GARP study and 26 age-matched healthy controls was used. Details on the generation of expression profiles and analysis are described earlier[13]. In short, blood of participants was collected and mononuclear blood cells were separated with Histopaque® gradient prior to RNA isolation. After RNA isolation the quality of 36 random samples was analyzed. Samples had an RNA integrity number (RIN) of 8.3 or higher, and an average 28S/18S ratio of 2.2 (range: 1.8-2.7). For cDNA synthesis, amplification, biotin labeling, and hybridization with 500ng of total RNA the Ambion® TotalPrep™-96 RNA amplification kit (Life Technologies) was used according to the manufacturer's protocol. Samples were

hybridized onto the microarrays (Illumina Human HT-12_v3_BeadChip's; Illumina). Quality control was performed in the R statistical programming language using the lumi-package. Total number of samples included in the current work is 134.

For the methylation analysis, genomic DNA was extracted with phenol-chloroform, and isolated DNA was treated with sodium bisulphite using the ZymoResearch EZ DNA Methylation kit. DNA methylation was assayed at over 450,000 sites on the Illumina Infinium HumanMethylation 450K BeadChips. Quality control was performed in the R statistical programming language using the minfi- and methylumi-package. Total number of samples with data on expression and methylation included in the current work is 120. The expression data is available at GEO (Gene Expression Omnibus) public repository under the accession GSE48556.

Genetic Epidemiology Network of Arteriopathy (GENOA)

The Genetic Epidemiology Network of Arteriopathy (GENOA) study consists of hypertensive sibships that were recruited for linkage and association studies in order to identify genes that influence blood pressure and its target organ damage[14]. In the initial phase of the GENOA study (Phase I: 1996-2001), all members of sibships containing ≥ 2 individuals with essential hypertension clinically diagnosed before age 60 were invited to participate, including both hypertensive and normotensive siblings.

The GENOA gene expression cohort is composed of 883 European-American individuals participating in the "Genetics of Microangiopathic Brain Injury" substudy, which investigates the genetic basis of alteration in brain structure detectable by magnetic resonance imaging[15].

RNA samples from cell lines established from peripheral blood samples by Epstein-Barr virus transformation were extracted using standard protocols. RNA quality was assessed using the Agilent 2100 Bioanalyzer (Agilent Technologies Inc., Foster City, CA) and quantified by spectrophotometry using the Nanodrop ND-1000 (Nanodrop Inc., Wilmington, DE). All RNA samples used in the present study yielded both an A260/A280 absorbance ratio greater than 2.0 and a RNA Integrity Number (RIN) ≥ 8. One µg of RNA was labeled using the WT Expression labeling assay (Applied Biosystems/ Ambion, Foster City, CA) including the labeling controls from the GeneChip Eukaryotic Poly-A RNA Control Kit (Affymetrix, Santa Clara, CA). Each step of the labeling protocol was monitored using the Agilent 2100 Bioanalyzer or the Nanodrop spectrophotometer, as recommended by the manufacturer. Labeled cRNAs were hybridized onto the Affymetrix Human Exon 1.0 ST Array.

Array quality control was performed using Affymetrix Expression Console™ (v 1.1) at the transcript level using core-level probe sets. All array images passed visual inspection. Hybridization controls were all present with signal increases following concentration. Labeling control signal strengths followed the order Lys < Phe < Thr < Dap. Signal intensity plots were examined for raw and processed data to identify outliers. Only core probesets were used to assess exon-level expression. Probe sets, which are known to cross-hybridize and those with undetectable expression were also excluded. Transcript-level expression was assessed by averaging all the core probe sets for that gene. QC metrics were again examined to identify possible outliers or non-performing samples using the Partek Genomic Suite software (V.6.6). These included the mean raw intensity for all probes, the distribution of RMA normalized intensities, and principal component analysis. We also verified sex assignment of the samples by examining expression levels of genes on the Y chromosome.

RNA quality control and microarray expression profiling experiments were conducted at the laboratory of Dr. Myriam Fornage at the University of Texas Health Science Center at Houston. The expression data is available at GEO (Gene Expression Omnibus) public repository under the accession GSE49531.

Grady Trauma Project (GTP)

The Grady Trauma Project (GTP) is a population-based, prospective study of demographic characteristics, trauma exposure, and prevalence of post-traumatic stress disorder and major depressive disorder in an urban, predominantly African-American population[16]. Subjects were recruited prospectively from the waiting rooms of primary care and obstetrics-gynecology clinics of Grady Memorial Hospital in Atlanta, GA. Exclusion criteria included mental retardation, active psychosis, or the inability to give informed consent. Written and verbal informed consent was obtained for all participating subjects. All procedures in

this study were approved by the Institutional Review Boards of Emory University School of Medicine and Grady Memorial Hospital. Since its inception in 2005, over 5000 subjects have been interviewed for the study.

For the expression analysis, whole blood was collected between 8 - 9 a.m. in Tempus RNA tubes for 398 subjects. All subjects were instructed to fast before blood collection. Whole genome expression profiles were generated for 398 subjects at the Max-Planck Institute as follows: RNA was isolated using the Versagene kit (Gentra Systems, Minneapolis, U.S.A.), quantified using the Nanophotometer and quality checks were performed on the Agilent Bioanalyzer. 250 nanograms of total RNA were reverse transcribed to cDNA, converted to cRNA and biotin-labeled using the Ambion kit (AMIL1791, Applied Biosystems), 750 nanograms of cRNA were hybridized to Illumina HT-12 v3.0 or v4.0 arrays (Illumina, San Diego, California, U.S.A) and incubated overnight for 16 hours at 55ºC. Arrays were washed, stained with Cy3 labeled streptavidin, dried and scanned on the Illumina BeadScan confocal laser scanner. 21,394 transcripts were on both the v3.0 and v4.0 arrays and were significantly expressed above background levels (detection $P<.05$) in at least 10% of subjects, and were thus eligible for further analysis. Of the initial 398 subjects, 359 subjects with self-reported African-American ancestry were selected for this analysis. Age of subjects at blood draw ranged from 16-78 years. The expression data is available at GEO (Gene Expression Omnibus) public repository under the accession GSE58137.

For the methylation analysis, we extracted DNA from whole blood at the Max Planck Institute in Munich using the Gentra Puregene Kit (Qiagen). Genomic DNA was then bisulfite converted using the Zymo EZ-96 DNA Methylation Kit (Zymo Research). We assessed DNA methylation at >480,000 CpG sites using Illumina HumanMethylation450 BeadChip arrays, with hybridization and processing performed according to the instructions of the manufacturer. For each CpG site and individual, we collected two data points: M (the total methylated signal), and U (the total unmethylated signal). We set to missing data points with 1) a detection p-value greater than 0.001 or 2) a combined signal less than 25% of the total median signal and less than both the median unmethylated and median methylated signal. We removed individual samples from analysis if they were outliers in a hierarchical clustering analysis or had 1) a mean total signal less than half of the median of the overall mean signal or 2000 arbitrary units, or 2) a missingness rate above 5%. Similarly, we removed from analysis CpG sites with a missingness rate above 10%. We then computed β-values for each individual at each CpG site as the total methylated signal divided by the total signal.

Heart and Vascular Health Study (HVH)

The Heart and Vascular Health (HVH) study[17-19] constitutes a group of population based case control studies of myocardial infarction (MI), stroke, venous thromboembolism (VTE), and atrial fibrillation[7]. Study participants were 30-79 year old members of Group Health, a large integrated health care organization in Washington State. Cases were identified from hospital discharge diagnosis codes and subsequently validated by medical record review. Cases shared a common control group that was a random sample of Group Health members, frequency-matched to MI cases on age (within decade), sex, treated hypertension, and calendar year of identification. The HVH study started in 1987 and blood specimen has been collected since 1995. Study eligibility, participant characteristics, and risk factor information were collected by medical record review and telephone interview. In addition, surviving cases and controls who agreed to participate had a blood draw.

Since 2003, whole blood has been collected in PAXGene tubes for mRNA expression studies. Participants of the current study (N=348) are those for whom expression profiling was done as part of several gene expression pilot studies conducted among HVH controls to investigate incident cardiovascular disease, hormone therapy, medications, diabetes, and atrial fibrillation. The Group Health human subjects review committee approved the study and all participants provided written informed consent.

Total RNA was extracted using PAXGene Blood RNA Kit and RNase-Free DNase Set (QIAGEN Inc., Valencia, CA) at the Fred Hutchinson Cancer Research Center, Seattle, WA. RNA integrity and quality was assessed using Agilent 2100 Bioanalyzer (Agilent Technologies Inc., Santa Clara, CA). Illumina® TotalPrep™-96 RNA Amplification Kit (Life Technologies Corp., Carlsbad, CA) was used for RNA amplification and labeling using manufacturer's instructions. Labeled cRNAs were hybridized onto Illumina HumanHT-12v3 and Illumina HumanHT-12v4 Expression Beadchip (Illumina, San Diego, CA) arrays, according to manufacturer's protocols. The images of the array chips were captured using an Illumina Beadarray scanner and scanned array images were imported into Illumina's GenomeStudio Gene Expression

Module. RNA quality control and microarray expression profiling experiments were conducted at the laboratory of Dr. Jerome Rotter at the Medical Genetics Institute at Cedars-Sinai Medical Center, Los Angeles, CA. The expression data is available at GEO (Gene Expression Omnibus) public repository under the accession GSE47729.

Boston Cohort (CD4+ T cells and CD14+ monocytes)

Healthy European-American individuals were sampled from the population of Boston, Massachusetts (n=211). Blood samples from each individual underwent flow cytometric purification to independently isolate CD4+CD8-CD3+ T cells and CD14+CD16- monocytes. Resulting cDNA was profiled using Affymetrix ST1.0 HuGene arrays. The sampled individuals ranged in age from 18 to 54 years of age. The expression data is available at GEO (Gene Expression Omnibus) public repository under the accession GSE56035.

Invecchiare in Chianti, ageing in the Chianti area (InCHIANTI)

The InCHIANTI study (*http://www.inchiantistudy.net*)[20] is a population-based, prospective study of human ageing in the Tuscany area of Italy. 1,455 participants were enrolled at baseline (1998-2000), with follow-up waves every 3 years. Extensive interviews, questionnaires, medical examinations, physical tests and blood samples were taken at every wave. Ethical approval was granted by the Instituto Nazionale Riposo e Cura Anziani institutional review board in Italy, and participants gave informed consent to participate.

Peripheral blood specimens were collected at wave 4 (year 9, 2008⁄9) from 712 individuals, using the PAXGene technology to preserve levels of mRNA transcripts as they were at the point of collection[21]. RNA was extracted from peripheral blood samples using the PAXGene Blood mRNA kit (Qiagen, Crawley, UK) according to the manufacturer’s instructions. RNA was biotinylated and amplified using the Illumina® TotalPrep[22] -96 RNA Amplification Kit and directly hybridized with HumanHT-12_v3 Expression BeadChips that include 48,803 probes. Image data was collected on an Illumina iScan and analyzed using the Illumina and Beadstudio software (Illumina, San Diego, California, USA) as previously described[23]. All microarray experiments and analyses complied with MIAME guidelines. The total number of InCHIANTI samples with good quality whole-genome expression data equals 698, 695 of which also have cell-count data.

CpG methylation data was generated for a subset of the samples seen during follow-up wave 3 (the ‘gene expression’ wave) using the Illumina Infinium HumanMethylation450 BeadChip. Briefly, genomic DNA was bisulfite converted using Zymo EZ-96 DNA Methylation Kit, followed by CpG analysis using the aforementioned Illumina 450k array. Quality control of the samples included exclusion based on sex-discrepancy and call-rate thresholds. Normalization of the arrays was performed using the ‘wateRmelon’[24] R package (specifically the DASEN method), which includes quantile normalization between probe-types and arrays. Samples having 5% of sites with a detection P-value>0.01 were removed. After exclusions, 506 samples had robust data available for analysis [25]. The expression data is available at GEO (Gene Expression Omnibus) public repository under the accession GSE48152.

KOoperative gesundheitsforschung in der Region Augsburg (KORA)

KORA F4 (Cooperative Heath Research in the Region of Augsburg) is a population-based survey in the region of Augsburg in Southern Germany. KORA exists since 1996 in the region of Augsburg in the southwest of Germany, and it is a regional research platform for population-based surveys and follow-up studies. Four cross-sectional health surveys have been performed in five-year intervals, each containing independent random samples of individuals with German nationality resident in Augsburg city or one of sixteen communities from the adjacent counties. 3,080 samples were collected for KORA F4 between 2006 and 2008[26]. The study followed the recommendations of the Declaration of Helsinki and was approved by the local ethical committees.

For the expression analysis, the subset from the survey F4 (2004-2006) of 1002 elderly individuals aged 61 to 82 years was used[27]. Gene expression profiling was performed using the Illumina HumanHT12v3 BeadChip as described elsewhere[28]. RNA was isolated from whole blood under fasting conditions using PAXGene Blood miRNA Kit (Qiagen, Hilden, Germany). Purity and integrity of the RNA was analysed using the Agilent Bioanalyzer with the 6000 Nano LabChip reagent set (Agilent Technologies, Germany). Samples with low quality were excluded after manually inspection. Using the Illumina TotalPrep-96 RNA Amp Kit

(Ambion), 500ng of RNA was reverse transcribed into cRNA, and biotin-UTP-labelled. 3000ng of cRNA was hybridized to the Illumina HumanHT12v3 Expression BeadChips, followed by washing steps as described in the Illumina protocol. After quality control, 993 samples were available for analysis. The expression data is available at ArrayExpress public repository under the accession E-MTAB-1708.

Genome-wide DNA methylation patterns were analyzed using the Infinium HumanMethylation450 BeadChip array (Illumina) as described elsewhere[29]. The percentage of methylation at a given cytosine is reported as a β-value, which is a continuous variable between 0 and 1, corresponding to the ratio of the methylated signal over the sum of the methylated and unmethylated signals. The M-value is calculated as the log2 ratio of the intensities of methylated probe vs. unmethylated probe[30].

Raw methylation data were extracted with Illumina Genome Studio (version 2010.3) with methylation module (version 1.8.5) and preprocessed using R (version 3.0.1). For data pre-processing of the Infinium Human Methylation 450K BeadChip we used the pipeline described earlier with default parameter settings to avoid bias in the analysis since the assay combines two different chemistries[31]. In brief, CpG sites in close proximity (50bp) to common SNPs and on allosomal positions were removed and color bias adjustment based on a smooth quantile normalization method as well as background level correction based on negative-control probes was performed for each chip using the R lumi package[32]. Finally, the pipeline performs subset quantile normalization, including only probes with detection p-values < 0.01, in order to correct for the InfI/InfII shift and normalize between samples. Therefore, the target CpGs were separated into different categories using CpG island annotation. For analysis we considered only beta-values associated with detection p-value < 0.01. Three samples were excluded due to chip wise detection rate < 80%.

Multi-Ethnic Study of Atherosclerosis (MESA)

The MESA study was designed to investigate the prevalence, correlates, and progression of subclinical cardiovascular disease in a population cohort of 6,814 participants. Since its inception in 2000 there have been five clinic visits during which extensive clinical, socio-demographic, lifestyle and behavior, laboratory, nutrition, and medication data were collected[33]. The present analysis is primarily based on analyses of purified monocyte samples from the most recent examination (April 2010-February 2012) from 734 randomly selected MESA participants, 55-94 years old, from four MESA field centers (Baltimore, MD; Forsyth County, NC; New York, NY; and St. Paul, MN).

Centralized training of technicians, standardized protocols, and numerous quality control (QC) measures were implemented for collection, on-site processing, and shipment of MESA specimens and routine calibration of equipment. Blood was initially collected in sodium heparin-containing Vacutainer CPTTM cell separation tubes (Becton Dickinson, Rutherford, NJ) to separate peripheral blood mononuclear cells from other elements within 2 hours from blood draw. Subsequently, monocytes were isolated with the anti-CD14 coated magnetic beads, respectively, using AutoMACs automated magnetic separation unit (Miltenyi Biotec, Bergisch Gladbach, Germany). Based on flow cytometry analysis of 18 specimens, monocyte samples were consistently greater than 90% pure.

DNA and RNA were isolated from samples simultaneously using the AllPrep DNA/RNA Mini Kit (Qiagen, Inc., Hilden, Germany). QC metrics were examined on the DNA and RNA samples, including optical density[22] measurements, using a NanoDrop spectrophotometer and evaluation of the integrity of the 18s and 28s ribosomal RNA. RNA QC testing was performed using the Agilent 2100 Bioanalyzer with RNA 6000 Nano chips (Agilent Techonology, Inc., Santa Clara, CA) according to the manufacturer's instructions. RNA with RIN (RNA Integrity) scores > 9.0 was used for global gene expression microarrays.

The Illumina HumanHT-12 v4 Expression BeadChip and the Illumina Bead Array Reader were used to perform the genome-wide expression analysis, following the Illumina expression protocol. The Illumina TotalPrep-96 RNA Amplification Kit (Ambion/Applied Biosystems, Darmstadt, Germany) was used for reverse transcription, and amplification with 500 ng of input total RNA (at 11ul). 700 ng of biotinylated cRNA was hybridized to a BeadChip at 58°C for 16–17 hours. The expression data is available at GEO (Gene Expression Omnibus) public repository under the accession GSE56045.

North American Brain Expression Consortium (NABEC) and United Kingdom Brain Expression Consortium (UKBEC)

Frozen frontal cortex (FCTX) and cerebellum (CRBL) samples were obtained from > 399 self-reported European ancestry samples without determinable neuropathological evidence of disease [23,34-36]. mRNA expression levels were assayed using Illumina HumanHT-12 v3 Expression Beadchips. In brief, individual probes were excluded from analyses if the p-value for detection was > 0.01 or there was less than 95% completeness of data per probe, and samples were excluded if <95% of probes were detected. Probes were also removed if an analyzed SNP was within the probe or the probe mapped ambiguously to multiple locations in the genome.

Expression data was quantile normalized and log2-transformed prior to analyses. Analyses were carried out separately for each brain region. The total number of NABEC and UKBEC samples with good quality whole-genome expression data was 394. The expression data is available at GEO (Gene Expression Omnibus) public repository under the accession GSE36192.

For the methylation analysis, genomic DNA was extracted with phenol-chloroform. Bisulfite converted DNA and assayed at > 27,000 sites on the Illumina Infinium HumanMethylation 27K BeadChips.

NIDDK-Phoenix Study (NIDDK/PHOENIX)

Subjects for the NIDDK-Phoenix study were selected from among participants in the Phoenix extension of the Family Investigation of Nephropathy and Diabetes[37]. In this study, American Indians from urban Phoenix, Arizona, are invited to have a screening examination for diabetes and nephropathy; beginning in 2008 a PAXGene RNA tube (BD) was collected at each examination. Individuals for the present study were selected among 1961 participants who had been fasting for ≥8 hours, who had been examined between 06:30 and 12:30 hours, and who did not have end-stage renal disease. The self-reported heritage of each individual was ≥½ American Indian, and all participants were ≥ 18 years old. Information on family membership was collected, and participants with diabetes or kidney disease were encouraged to refer family members. A total of 1461 individuals were selected for expression studies: 768 from pedigrees with ≥2 individuals available, and an additional 693, selected at random from the remaining potentially eligible individuals. Informed consent was obtained from each participant, and the Institutional Review Board of the NIDDK approved the study.

Total RNA was isolated using PAXGene Blood miRNA kits (Qiagen) according to the manufacturer's instructions. Samples were amplified and labelled (Ambion Message Ii Biotin-Enhanced aRNA amplification kit), and hybridized to the Illumina HumanHT-12 v4 Expression Beadchips as described by the manufacturer's protocol. Samples were scanned on the Illumina BeadArray 500GX Reader and the Illumina GenomeStudio software was used to perform a standard background normalization prior to exporting the data for statistical analysis. Four samples (2 from the family sample and 2 from the random sample) showed little evidence of overall expression above background levels and were excluded from analyses. Expression studies for the NIDDK-Phoenix samples were performed at the Department of Genetics Laboratories at Texas Biomedical Research Institute (San Antonio, Texas). The total number of NIDDK-Phoenix samples with good quality whole-genome expression data was 1457. Data are not deposited in public databases, but may be made available to potential collaborators, subject to IRB approval.

PBMC-MS

The PBMC-MS subjects were selected from the Comprehensive Longitudinal Investigation of Multiple Sclerosis at the Brigham and Women's Hospital (CLMB), which has been described earlier[38]. PBMCs were collected between 2002 and 2007, and they were frozen and stored in liquid nitrogen. Total RNA was isolated from RLT lysate using the RNeasy Mini kit (Qiagen) according to the manufacturer's instructions. After quality control (RIN >7), total RNA was amplified and labelled using the IVT RNA Amplification and Labeling system (Affymetrix, Santa Clara, CA). The dataset was preprocessed with SimpleAffy and quantile normalized following the GeneChip robust multiarray average (GCRMA) procedure in Bioconductor 2.8 (*www.bioconductor.org*). The limma R package was used for differential expression analysis[39]. Normalized data files and the .gct gene pattern compatible files were used in subsequent analyses. The expression data is available at GEO (Gene Expression Omnibus) public repository under the accession GSE16214.

Rotterdam Study (RS)

The Rotterdam Study (RS) (*www.epib.nl/rotterdamstudy)* is a prospective, population-based cohort study in the district of Rotterdam, the Netherlands, and has been described in detail[40]. The initial design of the study is straight-forward: a prospective cohort study among 7,983 persons living in the well-defined Ommoord district in the city of Rotterdam (78% of 10,215 invitees), called Rotterdam Study I (or RS-I). They were all 55 years of age or older and the oldest participant at the start was 106 years. The study started in the second half of 1989. In 1999, 3,011 participants (out of 4,472 invitees) who had become 55 years of age or moved into the study district since the start of the study were added to the cohort, called Rotterdam Study II (or RS-II). In 2006, a further extension of the cohort was initiated in which 3,932 subjects were included, aged 45 years and older (out of 6,057 invited), called Rotterdam Study III (RS-III).

The participants were all examined in some detail at baseline. They were interviewed at home and then had an extensive set of examinations in a specially built research facility in the centre of their district. These examinations were repeated every 3–4 years in characteristics that could change over time. The participants in the Rotterdam Study are followed for a variety of diseases that are frequent in the elderly. Informed consent was obtained from each participant, and the medical ethics committee of the Erasmus Medical Center Rotterdam approved the study.

For the expression analyses, the RS-III cohort was used: whole-blood was collected (PAXGene Tubes – Becton Dickinson) and total RNA was isolated (PAXGene Blood RNA kits - Qiagen). To ensure a constant high quality of the RNA preparations, all RNA samples were analysed using the Labchip GX (Calliper) according to the manufacturer's instructions. Samples with an RNA Quality Score > 7 were amplified and labelled (Ambion TotalPrep RNA), and hybridized to the Illumina HumanHT12v4 Expression Beadchips as described by the manufacturer's protocol. Processing of the Rotterdam Study RNA samples was performed at the Genetic Laboratory of Internal Medicine, Erasmus University Medical Center Rotterdam. The RS-III expression dataset is available at GEO (Gene Expression Omnibus) public repository under the accession GSE33828: 881 samples are available for analysis.

For the methylation analysis, genomic DNA was extracted from peripheral venous blood using the salting-out method[41]. DNA methylation was assayed at over 450,000 sites on the Illumina Infinium HumanMethylation 450K BeadChips. Quality control was performed in the R statistical programming language using the minfi- and methylumi-package[7,42]. Total number of samples having both gene expression and methylation data is 726.

San Antonio Family Heart Study (SAFHS)

The longitudinal San Antonio Family Heart Study (SAFHS, funded by the long-running NHLBI program project 5P01HL04552, PI Blangero) started in 1991 and was designed to primarily investigate the genetics of cardiovascular disease and its risk factors in Mexican Americans. The SAFHS included 1,431 individuals in 42 extended families at baseline. Ascertainment occurred by way of random selection of an adult Mexican American proband, without regard to presence or absence of disease and almost exclusively from Mexican American census tracts in San Antonio. These individuals have been followed in a mixed longitudinal fashion, with the average participant having been seen ~2.5 times (ranging from 1 to 4 times). A wide range of phenotypic information is available. Blood samples were obtained in the morning after an overnight fast, and mRNA was isolated in lymphocytes only.

Expression profiles were generated for 1,371 lymphocyte samples (from the first clinic visit of study participants, and subsequently stored at -80C w/o interruption before processing) in total, including various controls and duplicates, using Illumina's WG6v1 microarrays (47,293 probes in total). This is an early generation Illumina platform, which did not include all 2,238 age-associated genes for replication. We identified "good" expression data samples based on the number of detected probes (at detection p-values <= 0.05), mean average raw signal across all transcripts, and mean correlation against all other samples across all probes. Samples falling within the bottom 5% in each of these 3 quality measures were deleted, as were controls. Among duplicate samples, we kept the one giving the "best" quality results based on these measures. At the end of the day, this resulted in 1,244 samples with high quality expression data[43]. The gene expression data was then processed by background noise subtraction, log2 transformation, and quantile normalization.

We used SOLAR[44] to analyze what transcripts were associated with age. A random effects "polygenic" model, based on the kinship matrix of study participants, was used to model the genetic non-independence of family members based on their expected average autosomal sharing based on stated (and also

statistically verified) pedigree relationships, and age (at time of blood draw) was included as an additional covariate in the analysis. The expression data is available at the ArrayExpress public repository under the accession E-TABM-305.

Study of Health In Pomerania (SHIP-TREND)

SHIP (Study of Health in Pomerania) is a longitudinal population-based project consisting of two independent cohorts, SHIP and SHIP-TREND, assessing the prevalence and incidence of common, population relevant diseases and their risk factors. Baseline examinations for SHIP-TREND were conducted between 2008 and 2012. The study region of SHIP is West Pomerania, a region in the north-east of Germany. Stratification variables for sampling from population registries were age and sex. Study design and sampling methods were previously described[45,46]. A total number of 4420 Caucasian adults aged 20 to 79 years (response 50.1%) participated in the baseline SHIP-TREND. The medical ethics committee of the University of Greifswald approved the study protocol, and oral and written informed consents were obtained from all study participants.

For this analysis, a subset of the SHIP-TREND cohort with available phenotype and gene expression data (n=991) was used: RNA was prepared from whole blood under fasting conditions in PAXGene tubes (BD) using the PAXGene Blood miRNA Kit (Qiagen, Hilden, Germany): this was done on a QIAcube according to protocols provided by the manufacturer (Qiagen). To ensure a constant high quality of the RNA preparations, all RNA samples were analysed using RNA 6000 Nano LabChips (Agilent Technologies) on a 2100 Bioanalyzer (Agilent Technologies) according to the manufacturer’s instructions. Using the Illumina TotalPrep-96 RNA Amp Kit (Ambion), 500ng of RNA was reverse transcribed into cRNA, and biotin-UTP-labelled. 3000ng of cRNA were hybridized to the Illumina Whole-Genome Human HT12v3 Expression BeadChips, followed by washing steps as described in the Illumina protocol. Processing of the SHIP-TREND RNA samples was performed at the Helmholtz Zentrum München. The expression data is available at GEO (Gene Expression Omnibus) public repository under the accession GSE36382.

SUPPLEMENTARY NOTE 2

Additional acknowledgements

The infrastructure for the CHARGE Consortium is supported in part by the National Heart, Lung, and Blood Institute grant R01HL105756. This study was funded by the European Commission (HEALTH-F2-2008-201865, GEFOS; HEALTH-F2-2008 35627, TREAT-OA), the Netherlands Organization for Scientific Research (NWO) Investments (nr. 175.010.2005.011, 911-03-012), the Netherlands Consortium for Healthy Ageing[9], the Netherlands Genomics Initiative (NGI) / Netherlands Organization for Scientific Research (NWO) project nr. 050-060-810 and VIDI grant 917103521. Additionally, this work was supported by the German Federal Ministry of Education and Research (BMBF) within the framework of the e:Med research and funding concept (grant 01ZX1313A-2014).

BSGS

The Brisbane Systems Genetics Study (BSGS) is supported by the Australian National Health and Medical Research Council (NHMRC) grants 389892, 496667, 613601, 1010374 and 1046880, the Australian Research Council (ARC) grant (DE130100691), and by National Institutes of Health (NIH) grants GM057091 and GM099568. We also acknowledge Anthony Caracella and Lisa Bowdler for sample collection and processing.

DILGOM

Dietary, Lifestyle, and Genetic determinants of Obesity and Metabolic syndrome study (DILGOM). We thank the participants of the DILGOM study. Study was supported by Academy of Finland (DILGOM grant # 118065, 140747 to I.H. 251217 to S.R., 139635 to V.S. and 283045, 266199 to J.K.), the Finnish foundation for Cardiovascular Research to S.R., V.S. and M.P., the Sigrid Juselius Foundation to S.R. and I.H. and Biocentrum Helsinki to S.R.

EGCUT

EGCUT received financing from FP7 programs (ENGAGE, OPENGENE), targeted financing from Estonian Government SF0180142s08, Estonian Research Roadmap through Estonian Ministry of Education and Research, Center of Excellence in Genomics (EXCEGEN) and University of Tartu (SP1GVARENG), and from Estonian Research Council IUT2-2 grant and European Regional Development Fund. Also we acknowledge EGCUT technical personnel, especially Mr V. Soo and S. Smit. Data analyses were carried out in part in the High Performance Computing Center of University of Tartu.

FEHRMANN

This work is supported by the Netherlands Organization for Scientific Research [NWO-VENI grant 916.10.135 to LF], and a Horizon Breakthrough grant from the Netherlands Genomics Initiative [grant 92519031 to LF]. The research leading to these results has received funding from the European Community's Health Seventh Framework Programme (FP7/2007–2013) under grant agreement n° 259867. This study was financed in part by the SIA-raakPRO subsidy for project BioCOMP.

FHS

The National Heart, Lung, and Blood Institute's Framingham Heart Study is supported by NIH grant NO1-HC-25195. The Systems Approach to Biomarker Research in Cardiovascular Disease (SABRe CVD) Initiative is funded by the Division of Intramural Research, National Heart, Lung, and Blood Institute, Bethesda, MD. Dr. J. M. Murabito is supported by R01 AG29451. Dr. R. Joehanes, D. Levy, P. Munson and A.D. Johnson were supported by NHLBI Intramural Funds.

GARP

This study was supported by the Leiden University Medical Centre and the Dutch Arthritis Association. Pfizer Inc., Groton, CT, USA supported the inclusion of the GARP study. The research leading to these results has received funding from the European Union's Seventh Framework Programme (FP7/2007-

2011) under grant agreement n° 259679 and from BBMRI-NL, a research infrastructure financed by the Dutch government: NWO 184.021.007.

GENOA

Support for the Genetic Epidemiology Network of Arteriopathy (GENOA) was provided by the National Heart, Lung and Blood Institute of the National Institutes of Health (HL054464, HL054457, HL054481, HL087660, and NS041558). We would also like to thank the families that participated in the GENOA study.

GTP

This work was primarily supported by the National Institute of Mental Health (MH096764 and MH071537 to K.J.R.). Support was also received from Emory and Grady Memorial Hospital General Clinical Research Center, the Max Planck Society, the Behrens-Weise Foundation, NIH National Centers for Research Resources (M01RR00039), and the National Center for Advancing Translational Sciences of the NIH (UL1TR000454).

HVH

HVH was supported in part by grants R01 HL085251 and R01 HL073410 from the National Heart, Lung, and Blood Institute (NHLBI). The provision of genomic data was supported in part by the National Center for Advancing Translational Sciences, CTSI grant UL1TR000124, and National Institute of Diabetes and Digestive and Kidney Disease Diabetes Research Center (DRC) grant DK063491 to the Southern California Diabetes Research Center. The content is solely the responsibility of the authors and does not necessarily represent the official views of the NHLBI or the National Institutes of Health, USA. Dr. Psaty serves on a DSMB for a clinical trial of a device funded by Zoll LifeCor and on the Steering Committee for the Yale Open Data Access Project funded by Johnson & Johnson. Both activities are unrelated to this work.

InCHIANTI

This study was supported in part by the Intramural Research Program, National Institute on Ageing. UK based work was generously supported by a Wellcome Trust Institutional Strategic Support Award (WT097835MF), plus internal University of Exeter Medical School funding. This work has made use of the resources provided by the University of Exeter Science Strategy and resulting Systems Biology initiative.

The Jackson Laboratory

This work was supported by a Nathan Shock Center grant AG 25707 from the National Institute of Ageing, NIH, USA and by an Ellison Medical Foundation Senior Investigator Award to Beverly Paigen. We thank Scott Leiser and Matt Kaeberlein (University of Washington, Seattle, WA, USA) as well as Jarod Rollins, Aric Rogers, and Dustin Updike (Mount Desert Island Biological Laboratory, Bar Harbor, ME, USA) for useful discussion in resolving technical problems. *C. elegans* reagents and experiments were also supported by NHLBI intramural funds from A.D.J.

KORA

The KORA research platform and the KORA Augsburg studies are financed by the Helmholtz Zentrum München, German Research Center for Environmental Health, which is funded by the BMBF and by the State of Bavaria. Furthermore, KORA research was supported within the Munich Center of Health Sciences (MC Health), Ludwig Maximilians-Universität, as part of the LMUinnovative and by a grant from the German Federal Ministry of Education and Research (BMBF) to the German Center for Diabetes Research (DZD e. V.).This work was supported by the Ministry of Science and Research of the State of North Rhine-Westphalia (MIWF NRW) and the German Federal Ministry of Health (BMG).. Additional support was obtained from the BMBF (National Genome Research Network NGFNplus Atherogenomics, 01GS0834), by the DZHK (Deutsches Zentrum für Herz-Kreislauf-Forschung – German Centre for Cardiovascular Research) and from the European Commission's Seventh Framework Programme (FP7/2007-2013, HEALTH-F2-2011, grant agreement No. 277984, TIRCON and HEALTH-F2-2013, grant agreement No. 603288, SysVasc). We

thank the field staff in Augsburg who was involved in the conduct of the studies. We thank Katja Junghans and Anne Löschner (Helmholtz Zentrum München) for excellent technical assistance.

MESA

This research was supported by contracts N01-HC-from the National Heart, Lung and Blood Institute and by grants UL1-RR-024156 and UL1-RR-025005 from the National Center for Research Resources. The MESA Epigenomics & Transcriptomics Study was funded by NHLBI grant R01HL101250 to Wake Forest University Health Sciences. The authors thank the other investigators, the staff, and the participants of the MESA study for their valuable contributions. A full list of participating MESA investigators and institutions can be found at *http://www.mesa-nhlbi.org*.

NABEC and UKBEC

The work performed by the North American Brain Expression Consortium (NABEC) was supported in part by the Intramural Research Program of the National Institute on Ageing, National Institutes of Health, part of the US Department of Health and Human Services; project number ZIA AG000932-04. In addition this work was supported by a Research Grant from the Department of Defense, W81XWH-09-2-0128. This study utilized the high-performance computational capabilities of the Biowulf Linux cluster at the National Institutes of Health, Bethesda, Md. (*http://biowulf.nih.gov*).

The work performed by the UK Brain Expression Consortium (UKBEC) was supported by the MRC through the MRC Sudden Death Brain Bank (C.S.), by a Project Grant (G0901254 to J.H. and M.W.) and by a Fellowship award (G0802462 to M.R.). D.T. was supported by the King Faisal Specialist Hospital and Research Centre, Saudi Arabia. Computing facilities used at King's College London were supported by the National Institute for Health Research (NIHR) Biomedical Research Centre based at Guy's and St Thomas' NHS Foundation Trust and King's College London. We would like to thank AROS Applied Biotechnology AS company laboratories and Affymetrix for their valuable input.

NIDDK/PHOENIX

This work was supported by the intramural research program of the National Institute of Diabetes and Digestive and Kidney Diseases, Bethesda, MD.

PBMC-MC and the Boston Cohort (CD4+ and CD14+ cells)

T.R. is supported by the NIH F32 Fellowship (F32 AG043267). We thank all study participants in the Phenogenetic project for their contributions.

RS

The Rotterdam Study is funded by Erasmus Medical Center and Erasmus University, Rotterdam, Netherlands Organization for the Health Research and Development (ZonMw), the Netherlands Organisation of Scientific Research NWO Investments (nr. 175.010.2005.011, 911-03-012), the Research Institute for Diseases in the Elderly (014-93-015; RIDE2), the Ministry of Education, Culture and Science, the Ministry for Health, Welfare and Sports, the European Commission (DG XII), and the Municipality of Rotterdam. The authors are grateful to the study participants, the staff from the Rotterdam Study and the participating general practitioners and pharmacists.

The generation and management of RNA-expression array data for the Rotterdam Study was executed and funded by the Human Genotyping Facility of the Genetic Laboratory of the Department of Internal Medicine, Erasmus MC, the Netherlands. We thank Ms. Mila Jhamai, Ms. Jeannette M. Vergeer-Drop, Ms. Bernadette van Ast-Copier, and Mr. Marijn Verkerk for their help in creating the RNA array expression database.

SAFHS

SAFHS was supported in this effort by funding from R01AG031277 (SWB), P01HL045522 (JB), R37MH059490 (JB) and the Azar Foundation (JB).

SHIP-TREND

SHIP is part of the Community Medicine Research net of the University of Greifswald, Germany, which is funded by the Federal Ministry of Education and Research (grants no. 01ZZ9603, 01ZZ0103, and 01ZZ0403), the Ministry of Cultural Affairs as well as the Social Ministry of the Federal State of Mecklenburg-West Pomerania, and the network ‘Greifswald Approach to Individualized Medicine (GANI_MED)’ funded by the Federal Ministry of Education and Research (grant 03IS2061A). Genome-wide data have been supported by the Federal Ministry of Education and Research (grant no. 03ZIK012) and a joint grant from Siemens Healthcare, Erlangen, Germany and the Federal State of Mecklenburg, West Pomerania. The University of Greifswald is a member of the 'Center of Knowledge Interchange' program of the Siemens AG and the Caché Campus program of the InterSystems GmbH.

Individuals not attached to cohorts

AZ is supported by “IN-CONTROL” grant by Cardiovascular Research Netherlands (CVON), and from Rosalind Franklin Fellowship, University of Groningen, the Netherlands.

The authors are very grateful to the participants, the staff, the general practitioners, and the pharmacist from all participating studies.

SUPPLEMENTARY REFERENCES

1 Falchi, M. *et al.* Genome-wide association study identifies variants at 9p21 and 22q13 associated with development of cutaneous nevi. *Nature Genetics* **41**, 915-919 (2009).

2 Perola, M. *et al.* Combined genome scans for body stature in 6,602 European twins: evidence for common Caucasian loci. *PLoS Genet* **3**, e97 (2007).

3 Powell, J. E. *et al.* The Brisbane Systems Genetics Study: genetical genomics meets complex trait genetics. *PLoS One* **7**, e35430 (2012).

4 Powell, J. E. *et al.* Congruence of additive and non-additive effects on gene expression estimated from pedigree and SNP data. *PLoS Genet* **9**, e1003502 (2013).

5 McRae, A. F. *et al.* Contribution of genetic variation to transgenerational inheritance of DNA methylation. *Genome Biol* **15**, R73 (2014).

6 Inouye, M. *et al.* An immune response network associated with blood lipid levels. *PLoS Genet* **6**, e1001113 (2010).

7 Aryee, M. J. *et al.* Minfi: a flexible and comprehensive Bioconductor package for the analysis of Infinium DNA methylation microarrays. *Bioinformatics* **30**, 1363-1369 (2014).

8 Maksimovic, J., Gordon, L. & Oshlack, A. SWAN: Subset-quantile within array normalization for illumina infinium HumanMethylation450 BeadChips. *Genome Biol* **13**, R44 (2012).

9 Dubois, P. C. *et al.* Multiple common variants for celiac disease influencing immune gene expression. *Nature Genetics* **42**, 295-302 (2010).

10 Fehrmann, R. S. *et al.* Trans-eQTLs reveal that independent genetic variants associated with a complex phenotype converge on intermediate genes, with a major role for the HLA. *PLoS Genet* **7**, e1002197 (2011).

11 Feinleib, M., Kannel, W. B., Garrison, R. J., McNamara, P. M. & Castelli, W. P. The Framingham Offspring Study. Design and preliminary data. *Prev Med* **4**, 518-525 (1975).

12 Riyazi, N. *et al.* Evidence for familial aggregation of hand, hip, and spine but not knee osteoarthritis in siblings with multiple joint involvement: the GARP study. *Annals of the rheumatic diseases* **64**, 438-443 (2005).

13 Ramos, Y. F. *et al.* Genes expressed in blood link osteoarthritis with apoptotic pathways. *Annals of the rheumatic diseases* **73**, 1844-1853 (2014).

14 Daniels, P. R. *et al.* Familial aggregation of hypertension treatment and control in the Genetic Epidemiology Network of Arteriopathy (GENOA) study. *Am J Med* **116**, 676-681 (2004).

15 Turner, S. T. *et al.* Genomic susceptibility Loci for brain atrophy, ventricular volume, and leukoaraiosis in hypertensive sibships. *Arch Neurol* **66**, 847-857 (2009).

16 Gillespie, C. F. *et al.* Trauma exposure and stress-related disorders in inner city primary care patients. *Gen Hosp Psychiatry* **31**, 505-514 (2009).

17 Heckbert, S. R. *et al.* Antihypertensive treatment with ACE inhibitors or beta-blockers and risk of incident atrial fibrillation in a general hypertensive population. *Am J Hypertens* **22**, 538-544 (2009).

18 Psaty, B. M. *et al.* The risk of myocardial infarction associated with antihypertensive drug therapies. *JAMA* **274**, 620-625 (1995).

19 Smith, N. L. *et al.* Esterified estrogens and conjugated equine estrogens and the risk of venous thrombosis. *JAMA* **292**, 1581-1587 (2004).

20 Ferrucci, L. *et al.* Subsystems contributing to the decline in ability to walk: bridging the gap between epidemiology and geriatric practice in the InCHIANTI study. *J Am Geriatr Soc* **48**, 1618-1625 (2000).

21 Debey-Pascher, S., Eggle, D. & Schultze, J. L. RNA stabilization of peripheral blood and profiling by bead chip analysis. *Methods Mol Biol* **496**, 175-210 (2009).

22 Broer, L. *et al.* GWAS of Longevity in CHARGE Consortium Confirms APOE and FOXO3 Candidacy. *J Gerontol A Biol Sci Med Sci* (2014).

23 Gibbs, J. R. *et al.* Abundant quantitative trait loci exist for DNA methylation and gene expression in human brain. *PLoS Genet* **6**, e1000952 (2010).

24 Pidsley, R. *et al.* A data-driven approach to preprocessing Illumina 450K methylation array data. *BMC Genomics* **14**, 293 (2013).

25 Holly, A. C. *et al.* Splicing factor 3B1 hypomethylation is associated with altered SF3B1 transcript expression in older humans. *Mech Ageing Dev* **135**, 50-56 (2014).
26 Holle, R., Happich, M., Lowel, H. & Wichmann, H. E. KORA--a research platform for population based health research. *Gesundheitswesen* **67 Suppl 1**, S19-25 (2005).
27 Rathmann, W. *et al.* Incidence of Type 2 diabetes in the elderly German population and the effect of clinical and lifestyle risk factors: KORA S4/F4 cohort study. *Diabet Med* **26**, 1212-1219 (2009).
28 Mehta, D. *et al.* Impact of common regulatory single-nucleotide variants on gene expression profiles in whole blood. *Eur J Hum Genet* **21**, 48-54 (2013).
29 Zeilinger, S. *et al.* Tobacco smoking leads to extensive genome-wide changes in DNA methylation. *PLoS One* **8**, e63812 (2013).
30 Du, P. *et al.* Comparison of Beta-value and M-value methods for quantifying methylation levels by microarray analysis. *BMC Bioinformatics* **11**, 587 (2010).
31 Touleimat, N. & Tost, J. Complete pipeline for Infinium((R)) Human Methylation 450K BeadChip data processing using subset quantile normalization for accurate DNA methylation estimation. *Epigenomics* **4**, 325-341 (2012).
32 Du, P., Kibbe, W. A. & Lin, S. M. lumi: a pipeline for processing Illumina microarray. *Bioinformatics* **24**, 1547-1548 (2008).
33 Bild, D. E. *et al.* Multi-ethnic study of atherosclerosis: objectives and design. *Am J Epidemiol* **156**, 871-881 (2002).
34 Hernandez, D. G. *et al.* Integration of GWAS SNPs and tissue specific expression profiling reveal discrete eQTLs for human traits in blood and brain. *Neurobiol Dis* **47**, 20-28 (2012).
35 Trabzuni, D. *et al.* Quality control parameters on a large dataset of regionally dissected human control brains for whole genome expression studies. *J Neurochem* **119**, 275-282 (2011).
36 Trabzuni, D. *et al.* MAPT expression and splicing is differentially regulated by brain region: relation to genotype and implication for tauopathies. *Human molecular genetics* **21**, 4094-4103 (2012).
37 Knowler, W. C. *et al.* The Family Investigation of Nephropathy and Diabetes (FIND): design and methods. *J Diabetes Complications* **19**, 1-9 (2005).
38 Ottoboni, L. *et al.* An RNA profile identifies two subsets of multiple sclerosis patients differing in disease activity. *Sci Transl Med* **4**, 153ra131 (2012).
39 Smyth, G. K. *Bioinformatics and Computational Biology Solutions Using R and Bioconductor. Chapter 23: Limma. .* (Springer New York, 2005).
40 Hofman, A. *et al.* The Rotterdam Study: 2014 objectives and design update. *European journal of epidemiology* **28**, 889-926 (2013).
41 Miller, S. A., Dykes, D. D. & Polesky, H. F. A simple salting out procedure for extracting DNA from human nucleated cells. *Nucleic Acids Res* **16**, 1215 (1988).
42 Davis S, D. P., Bilke S, Triche T, Jr. , Bootwalla M, *Methylumi: Handle Illumina methylation data.* , R package version 2.12.0. (2014).
43 Goring, H. H. *et al.* Discovery of expression QTLs using large-scale transcriptional profiling in human lymphocytes. *Nature Genetics* **39**, 1208-1216 (2007).
44 Almasy, L. & Blangero, J. Multipoint quantitative-trait linkage analysis in general pedigrees. *Am J Hum Genet* **62**, 1198-1211 (1998).
45 John, U. *et al.* Study of Health In Pomerania (SHIP): a health examination survey in an east German region: objectives and design. *Soz Praventivmed* **46**, 186-194 (2001).
46 Volzke, H. *et al.* Cohort profile: the study of health in Pomerania. *Int J Epidemiol* **40**, 294-307 (2011).
